# Safety of single-dose bedaquiline combined with rifampicin for leprosy post-exposure prophylaxis: A Phase 2 randomized non-inferiority trial in the Comoros Islands

**DOI:** 10.1371/journal.pmed.1004453

**Published:** 2024-10-21

**Authors:** Bouke Catherine de Jong, Said Nourdine, Auke Thomas Bergeman, Zahara Salim, Silahi Halifa Grillone, Sofie Marijke Braet, Mohamed Wirdane Abdou, Rian Snijders, Maya Ronse, Carolien Hoof, Achilleas Tsoumanis, Nimer Ortuño-Gutiérrez, Christian van der Werf, Alberto Piubello, Aboubacar Mzembaba, Younoussa Assoumani, Epco Hasker

**Affiliations:** 1 Department of Biomedical Sciences, Institute of Tropical Medicine, Antwerp, Belgium; 2 National Tuberculosis and Leprosy control Program, Moroni, Union of the Comoros; 3 Heart Centre, Department of Cardiology, Amsterdam UMC location AMC, University of Amsterdam, Amsterdam, the Netherlands; 4 Department of Public Health, Institute of Tropical Medicine, Antwerp, Belgium; 5 Department of Clinical Sciences, Institute of Tropical Medicine, Antwerp, Belgium; 6 Damien Foundation, Brussels, Belgium; University of Glasgow, UNITED KINGDOM OF GREAT BRITAIN AND NORTHERN IRELAND

## Abstract

**Background:**

To reduce leprosy risk in contacts of patients with leprosy by around 50%, the World Health Organization (WHO) recommends leprosy post-exposure prophylaxis (PEP) using single-dose rifampicin (SDR). Results from a cluster randomized trial in the Comoros and Madagascar suggest that PEP with a double dose of rifampicin led to a similar reduction in incident leprosy, prompting the need for stronger PEP. The objective of this Phase 2 trial was to assess safety of a bedaquiline-enhanced PEP regimen (intervention arm, bedaquiline 800 mg with rifampicin 600 mg, BE-PEP), relative to the WHO recommended PEP with rifampicin 600 mg alone (control arm, SDR-PEP).

**Methods and findings:**

From July 2022 to January 2023, consenting participants were screened for eligibility, including a heart rate-corrected QT interval (QTc) <450 ms and liver enzyme tests (ALT/AST) below 3× the upper limit of normal (ULN), before they were individually randomized 1:1 in an open-label design. Recruitment was sequential, by age group. Pediatric dosages were weight adjusted. Follow-up was done at day 1 post-dose (including ECG) and day 14 (including ALT/AST), with repeat of ALT/AST on the last follow-up at day 30 in case of elevation on day 14. The primary outcome was non-inferiority of BE-PEP based on a <10 ms difference in QTc 24 h after treatment administration, both unadjusted and adjusted for baseline QTc.

Of 408 screened participants, 313 were enrolled, starting with 187 adults, then 38 children aged 13 to 17 years, and finally 88 children aged 5 to 12 years, of whom 310 (99%) completed all visits. Across all ages, the mean QTc change on BE-PEP was from 393 ms to 396 ms, not significantly different from the change from 392 ms to 394 ms on SDR-PEP (difference between arms 1.8 ms, 95% CI −1.8, 5.3, *p* = 0.41). No individual’s QTc increased by >50 ms or exceeded 450 ms after PEP administration. Per protocol, all children were analyzed together, with no significant difference in mean QTc increase for BE-PEP compared to SDR-PEP, although non-inferiority of BE-PEP in children was not demonstrated in unadjusted analysis, as the upper limit of the 95% CI of 10.4 ms exceeded the predefined margin of 10 ms. Adjusting for baseline QTc, the regression coefficient and 95% CI (3.3; −1.4, 8.0) met the 10 ms non-inferiority margin. No significant differences in ALT or AST levels were noted between the intervention and control arms, although a limitation of the study was false elevation of ALT/AST during adult recruitment due to a technical error. In both study arms, one serious adverse event was reported, both considered unlikely related to the study drugs. Dizziness, nausea, headache, and diarrhea among adults, and headaches in children, were nonsignificantly more frequently observed in the BE-PEP group.

**Conclusions:**

In this study, we observed that safety of single-dose bedaquiline 800 mg in combination with rifampicin is comparable to rifampicin alone, although non-inferiority of QTc changes was demonstrated in children only after adjusting for the baseline QTc measurements. A Phase 3 cluster randomized efficacy trial is currently ongoing in the Comoros.

**Trial Registration:**

ClinicalTrials.gov NCT05406479.

## Abstract 2

Bouke C. de Jong and team assess the safety of a Bedaquiline enhanced post-exposure prophylaxis regimen in the Comoros Islands.

## Author summary

**Why was this study done?:**

- Worldwide, leprosy is diagnosed in 200,000 persons annually.
- Those living near a patient with leprosy can reduce their risk of developing leprosy by half if they take a single dose of the antibiotic rifampicin, known as “post-exposure prophylaxis,” or PEP.
- Dual antibiotic treatment given as PEP may reduce the risk further, and bedaquiline has been shown to have a high degree of effectiveness against leprosy in animal studies, although the safety of PEP containing bedaquiline had not previously been tested.

**What did the researchers do and find?:**

- In a village in the Comoros affected by leprosy, first adults and then children were invited to participate in a randomized study in which they could receive either a single dose of the regular PEP, or bedaquiline-enhanced PEP, in which regular rifampicin PEP was combined with bedaquiline.
- They had their heart rhythm and liver enzymes checked before and after taking PEP to screen for asymptomatic drug toxicity.
- There was no statistically significant difference in the heart rhythm or liver function between the 2 groups. While the analysis in children showed that the spread around the difference just exceeded the predetermined safety margin, the analysis controlled for baseline values did not show increased risk.

**What do these findings mean?:**

- The findings suggest that regular PEP can safely be combined with bedaquiline, although it is not yet known if this provides stronger protection against developing leprosy.
- Limitations in this study include a technical error in liver function testing of adult participants, which may have hidden a low-level elevation.

## Introduction

The steady rate of leprosy notifications, which has remained approximately 200,000 annually worldwide in the 21st century [1], prompted the World Health Organization (WHO) in 2018 to conditionally recommend a single dose of 600 mg (10 mg/kg) rifampicin as post-exposure prophylaxis (PEP) for contacts of patients with leprosy [2]. This strategy had demonstrated a 57% decrease in the individual risk of leprosy over 2 years in the COLEP trial in Bangladesh [3]. Despite initial concerns about large-scale use of single-dose rifampicin (SDR) potentially triggering resistance in *Mycobacterium leprae* (*M*. *leprae*) and *M*. *tuberculosis*, a consensus published by experts suggested this was unlikely [4].

In an early *M*. *leprae* drug resistance survey conducted in the Comoros, no rifampicin resistance conferring mutations in *rpoB* were found, even among patients who developed leprosy after receiving rifampicin PEP [5]. Given the greater efficacy of higher doses of rifampicin, with demonstrated safety [6,7], we tested 1,200 mg (20 mg/kg) rifampicin PEP in a cluster-randomized trial in Comoros and Madagascar, which showed a reduction in leprosy risk of 45% [8]. While the individual protective effect of double-dose rifampicin in the Comoros and Madagascar was similar to the 57% observed with SDR in Bangladesh, we hypothesize that the higher incidence in the Comoros may require a stronger PEP regimen [3,9]. We therefore investigated alternative PEP regimens to effectively reduce the risk of leprosy. Bedaquiline, a significant breakthrough in treating multidrug-resistant tuberculosis (TB), has shown robust activity against *M*. *leprae* in mice [10,11]. The thorough QT interval clinical studies conducted prior to the approval of bedaquiline for treatment of drug-resistant TB confirmed its safety at a single-dose of 800 mg in healthy participants, as referenced in the product information [12]. In the studies exploring varying dosages of bedaquiline in patients with TB [13], the most effective dosage regimen involved a loading dose of 700 mg of bedaquiline, followed by a 500 mg dose on the second day. This regimen demonstrated the most potent initial bactericidal activity [14]. Preliminary results from a study in Mali, using monotherapy with daily bedaquiline 100 mg for 8 weeks for the treatment of multibacillary patients with leprosy, followed by a full course of WHO recommended multidrug therapy (MDT), showed near complete conversion to negative mouse footpad cultures at 8 weeks, suggesting high efficacy of bedaquiline in leprosy treatment [15]. Given these findings and the ongoing Phase 2 study for leprosy treatment with bedaquiline in Brazil (clinicaltrials.gov/NCT03384641), we tested the safety of PEP augmented with bedaquiline in a Phase 2 randomized trial in the Comoros (clinicaltrials.gov/NCT05406479), preceding a Phase 3 cluster randomized trial, called BE-PEOPLE (clinicaltrials.gov/NCT05597280) [16].

The primary objective of the Phase 2 trial was to assess safety of the bedaquiline-enhanced PEP regimen (bedaquiline 800 mg with rifampicin 600 mg), relative to the WHO recommended PEP with rifampicin 600 mg alone, specifically the mean difference in QTc interval between the 2 arms 24 h after treatment administration. The aim of this safety non-inferiority trial in a leprosy endemic field setting in the Comoros was to inform the Data Safety Monitoring Board’s decision on whether or not the Phase 3 trial could be implemented, taking into account QTc data, observed (serious) adverse events ((S)AEs) and predetermined stopping criteria. Given the safety profile of bedaquiline, experience with bedaquiline in the treatment of rifampicin resistant TB, and potential interactions with rifampicin, the QTc was monitored the day after study drug administration, and liver enzymes alanine (ALT) and aspartate (AST) aminotransferases were monitored 2 weeks after study drug administration.

Secondary objectives included determination of the baseline frequency of elevations of serum levels of liver enzymes and QTc prolongations in the population, and to document for each of the 2 regimens post administration QTc levels, as well as any potentially frequent adverse events such as gastrointestinal (nausea, vomiting), nervous system-related (headache, dizziness), and cutaneous reactions.

## Methods

### Study site and population

Following discussions with community leaders, a village on the island of Anjouan, Comoros, was selected as the study site. This village reported 12 leprosy cases from 2019 to 2022 among its approximately 800 residents. For this Phase 2 randomized non-inferiority trial, with allocation ratio 1:1, a temporary study center was established in the village where all procedures were conducted by the team, including an on-site medical doctor (pulmonary- or cardiology specialist). As part of the preceding PEOPLE trial [8,17], and following informed consent, the entire village had been screened for signs and symptoms of leprosy without receiving PEP. The rest of the population, not enrolled in the BE-PEOPLE randomized trial, and aged 2 and older, were offered SDR PEP through the National Tuberculosis and Leprosy Control Programme, per WHO guidelines [18].

### Inclusion/Exclusion criteria

Inclusion criteria were a good health status, residence in the study village and willingness to participate. Participants were excluded when they had signs or symptoms of active leprosy or TB, suspected or confirmed pregnancy or breastfeeding, age under 5 years or body weight under 20 kg, a history of liver or kidney disease, recent use (in the last 2 years) or allergic reaction to bedaquiline or rifampicin, the use of medication not considered compatible with bedaquiline [“Safe list” in Annex] in the last 3 weeks, inability to swallow 100 mg bedaquiline tablets, and those with baseline QTc >450 ms or, ALT or AST >3× upper limit of normal (ULN).

To limit risk to the extent possible, during enrolment the study population was split into 3 age groups, adults (18 years and above), children aged 13 to 17 years and children aged 5 to 12 years. Groups of participants were enrolled and treated in this order and results for each group were presented to the DSMB before the next group was enrolled.

### Procedures

**Fig 1:**
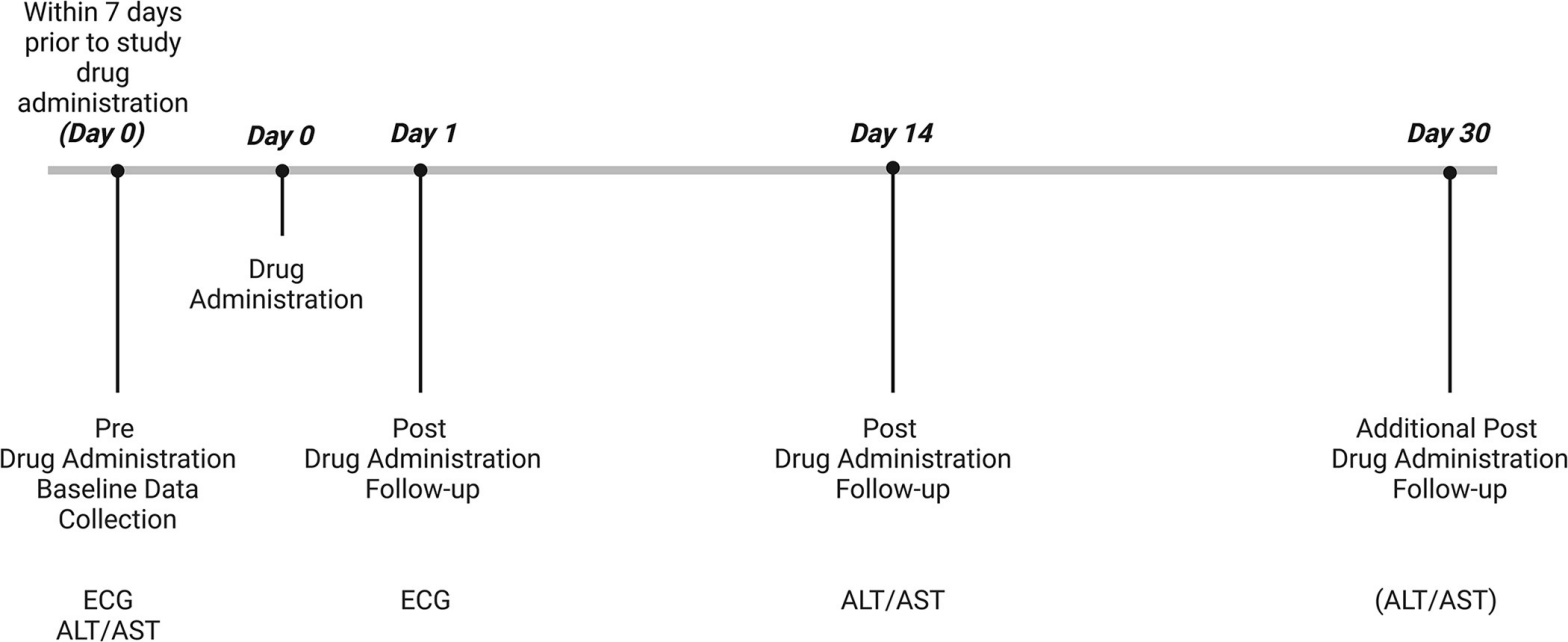
Study timeline. Created with Biorender.com.

After the informed consent procedure, participants had baseline data collected, including an electrocardiogram (ECG) and liver enzyme tests, which were reviewed for eligibility for randomization (Fig 1). Within 7 days of baseline data collection, eligible participants were individually randomized 1:1 in an open-label design to either the intervention group (1 single dose of 800 mg bedaquiline and 600 mg of rifampicin; BE-PEP) or the control group (1 single dose of 600 mg rifampicin alone; SDR-PEP), stratified by age-group.

Dosing was adjusted for weight as follows: 20 to 29 kg: bedaquiline: 400 mg, rifampicin: 300 mg; 30 to 45 kg: bedaquiline: 800 mg, rifampicin: 450 mg; and >45 kg: bedaquiline: 800 mg, rifampicin: 600 mg.

ECG: On completion of a 12-lead ECG (Cardiovit AT-1 G2, Schiller, Switzerland), QTc was measured using the tangent method, preferably in lead II. In the case of a biphasic T-wave, the largest deflection was used. U-waves were not included in the QT interval measurement. Correction for heart rate was performed using Fridericia’s formula (QTc = QT/RR^1/3^) and the preceding RR interval. The average of 3 QTc intervals was reported as the QTc on each ECG. In case of QTc prolongation >60 ms from Day 0 (= D0), or QTc >500 ms, participants would be urgently transferred to a specialist cardiology service for further examination, continuous rhythm monitoring, repeated QTc measurements, and measurement of potassium for correction of hypokalemia.

Hepatic function test were performed using the protocol dx.doi.org/10.17504/protocols.io.bp2l62e3kgqe/v1. The ULN values for ALT used for this study were 32 IU/L (women and children) and 39 IU/ml (men), and for AST 33 IU/L (women and children) and 41 IU/ml (men).

### Follow-up

Participant were invited to return to the study center on the day after study drug administration (= D1) to collect safety data, including QTc, and again 14 days after (= D14) to collect additional safety data, including venous blood for ALT/AST, and to follow up on any ongoing (S)AE’s. In case any SAE’s were ongoing during the visit on D14 additional visits were organized to follow up. A final visit to collect adverse events was organized on day 30 (= D30) at the participant’s house (Fig 1). In case of an ALT/AST elevation adverse event on visit D14 or absolute AST/ALT value increase of > = 35 between the prerecruitment visit and visit D14, liver enzyme tests were repeated again on visit D30.

### Randomization and blinding

The study was blinded to the investigators, including the referent qualified ECG reader, yet unblinded to the study personnel and patients, given the administration of the bedaquiline tablets, and the statistician, who prepared the randomization list and reports for the DSMB. All participants were informed about red discoloration of their urine due to rifampicin.

The randomization list was generated using SAS 9.4, using blocked randomization with block size 8. The statistician prepared the final randomization list, based on which envelopes were prepared for randomization during recruitment.

All ECGs were time stamped and uploaded in REDCap, and the QTc was re-measured by a referent qualified reader, blinded to treatment allocation and whether the recording was done before or after study drug ingestion, which values were used in the analysis.

### Outcomes and definitions

The primary outcome was the mean difference in QTc interval between the 2 arms 24 h after treatment administration.

Secondary outcomes were the baseline frequency of ALT and AST elevations and QTc prolongations in the population, and to document for each of the 2 regimens post administration any potentially frequent adverse events such as gastrointestinal (nausea, vomiting), nervous system-related (headache, dizziness), and cutaneous reactions.

Adverse events regarding ALT/AST values and QTc prolongations were graded using the Division of AIDS (DAIDS) scale, version 2.1. ALT and AST elevations were scored as grade 1 (mild) if 1.25 to <2.5× ULN, grade 2 (moderate) if 2.5 to <5.0× ULN, grade 3 (severe) if 5.0 to <10× ULN, and grade 4 (life threatening) if ≥10× ULN. Other AE’s were graded using the protocol descriptions for grading with mild (events require minimal or no treatment and do not interfere with the participant’s daily activities), moderate (events result in a low level of inconvenience or concern with the therapeutic measures, events may cause some interference with functioning), severe (events interrupt a participant’s usual daily activity and may require systemic drug therapy or other treatment, usually incapacitating), and life threatening events (participant at risk for death at the time of the event).

The description of the other AE’s followed terms from the Medical Dictionary for Regulatory Activities.

Prespecified criteria on stopping for the Phase 2 study were specified as follows:

1. Death of a participant considered related to the study drug.
2. One or more participants experiencing an SAE or Grade 4 AE or a persistent (upon repeat testing) Grade 4 laboratory abnormality that is determined to be related to the study drug.
3. Three or more participants experiencing a Grade 3 or greater AE of the same type (as per medical judgment) that is determined to be related to the study drug.
4. Three or more participants experiencing a persistent (upon repeat testing) Grade 3 laboratory abnormality related to the same laboratory parameter and considered to be related to the study drug.
5. Two or more participants experiencing QTc > 500 ms.
6. One or more participants having AST or ALT > 8× ULN, in absence of a causative explanation.

### Sample size and statistical analysis methods

The sample size was calculated to ensure a power of at least 80% in each of 2 age subgroups, i.e., adults and children (below 18 years of age). Based on an expected mean QTc of 384 ms with a standard deviation of 20 ms and a non-inferiority margin of +10 ms, at least 64 participants would be required in each group (intervention and control) for children and for adults. The choice for a relatively conservative non-inferiority margin of 10 ms, of limited clinical relevance, was to avoid obscuring individual differences, with the overall objective for the data safety and monitoring board (DSMB) to be able to assess safety. Taking into account the actual age distribution in the population 150 participants were recruited in each of the study groups, resulting in power of 99% for the total study population.

A prespecified plan for statistical analysis was followed, with the per protocol analysis as the primary results, according to the Good Clinical Practice guidelines for non-inferiority studies (ICH E9 guidelines). The intention to treat results are provided in S1 Table. In the per protocol analysis only participants who had received PEP as planned, had completed follow-up, and followed the protocol as planned were included. Both unadjusted and adjusted for baseline QTc measurements were used to assess the primary objective according to the pre-planned analytical plan. Safety analyses were performed to all participants who received at least 1 dose of the study medication.

The main objective was assessed by calculating the difference in QTc between the 2 arms 24 h after treatment administration and the corresponding one-sided 95% confidence interval, and examining if it contained the non-inferiority margin. The *p*-value tested the hypothesis that BE-PEP causes more QTc prolongation than SDR-PEP; a value below 0.025 indicates that this hypothesis is rejected. Additionally, linear regression models were fitted, using the QTc values 24 h after treatment administration as outcome and allocation arm and QTc values at baseline (D0) as predictors. The results were interpreted as average changes in QTc 24 h after administration for the BE-PEP groups compared to the SDR-PEP group.

All safety endpoints including serious adverse events in each age group were reviewed by an independent DSMB.

### Ethics and regulatory issues

The Phase 2 study specific informed consent procedure aimed for full comprehension of study procedures, including safety monitoring. Written informed consent was obtained. For children, the parent or guardian provided written informed consent, in addition to assent from children aged 12 to 17. For illiterate participants, an impartial witness observed and signed the consent procedure including the placement of a thumbprint.

The protocol was initially reviewed by the Federal Agency for Medicines and Health Products of Belgium (FAGG), through a formal Scientific and Technical Advice procedure. Following the implementation of this procedure, the protocol was approved by the Institute of Tropical Medicine’s Institutional Review Board, the Antwerp University Hospital’s ethics committee, and the National Ethics Committee of the Comoros [16]. The trial was sponsored by the Institute of Tropical Medicine and funded by Janssen Pharmaceuticals as part of an investigator-initiated collaboration and was registered in clinicaltrials.gov (NCT05406479).

## Results

**Fig 2:**
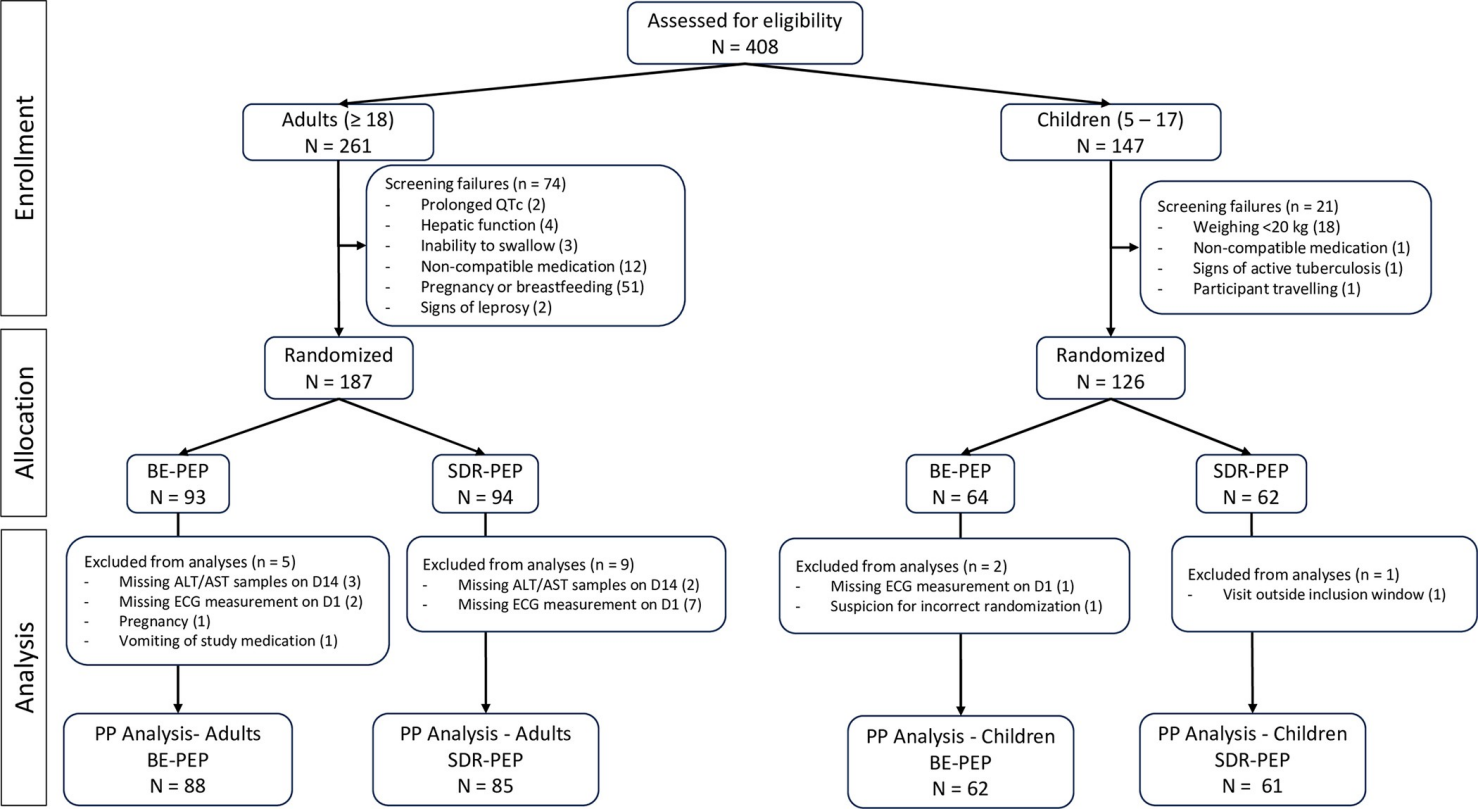
Participant accounting and inclusion in PP analysis. Children were recruited sequentially, first children aged 13–17 and then children aged 5–12. Data from children aged 13–17 (*n* = 36) and children aged 5–12 (*n* = 87) were analyzed together. BE-PEP, bedaquiline enhanced post-exposure prophylaxis; SDR-PEP, single-dose rifampicin PEP; ECG, electrocardiogram; ALT, alanine aminotransferase; AST, aspartate aminotransferase; PP, per protocol.

Recruitment took place from July 18, 2022 until December 27, 2022, with follow-up completed on January 26, 2023. Out of the 408 individuals screened for the study, 313 were eligible and agreed to participate, leading to their randomization into the study groups (Fig 2). The main reasons for exclusion from participation in the study were pregnancy or breastfeeding, which accounted for 69% of non-eligible adults (51 out of 74), and a weight of less than 20 kg as reason for exclusion in 18 out of the 20 excluded children. Two adults were excluded due to a prolonged QTc at baseline (451 ms and 480 ms). Given the low-level QTc elevations these persons were not referred for further assessment. Among randomized adults, more women were included (Table 1).

**Table 1:** Characteristics of the study population at randomization.

Age-group	Characteristic	Pooled *n* = 313 median (IQR)	BE-PEP *n* = 157 median (IQR)	SDR-PEP *n* = 156 median (IQR)
Adults (≥18)	Age	41 (32–53)	40 (31–53)	41 (32–55)
	BMI	25 (22–29)	25 (22–28)	25 (22–29)
	Height (cm)	158 (150–164)	158 (152–166)	157 (150–163)
	Sex (% Female)	109 (58)	57 (61)	52 (55)
	Weight (kg)	63 (56–72)	62 (56–72)	63 (57–70)
Children	Age	11 (8–14)	11 (9–14)	11 (8–14)
	BMI	17 (15–19)	17 (15–20)	16 (15–18)
	Height (cm)	141 (128–156)	141 (130–154)	141 (128–158)
	Sex (% Female)	58 (46)	27 (42)	31 (50)
	Weight (kg)	34 (25–46)	35 (26–47)	33 (25–45)

Participants in each treatment group and overall (“Pooled”) were described with respect to selected baseline characteristics. The description was in terms of medians and interquartile ranges for continuous characteristics and using counts and percentages for categorical characteristics. BMI, body mass index; BE-PEP, post-exposure prophylaxis (PEP) with bedaquiline 800 mg + rifampicin 600 mg; SDR-PEP, PEP with rifampicin 600 mg only.

Out of 313 enrolled and randomized, 310 completed all required visits, consisting of 184 adults, 38 children aged 13 to 17 years, and 88 children aged 5 to 12 years. In the per protocol analysis, 296 participants were included (Fig 2). One adult participant was excluded from per protocol analysis due to vomiting the administered study medication shortly after ingestion. One participant was excluded as he likely received investigational product belonging to the incorrect arm.

**Fig 3:**
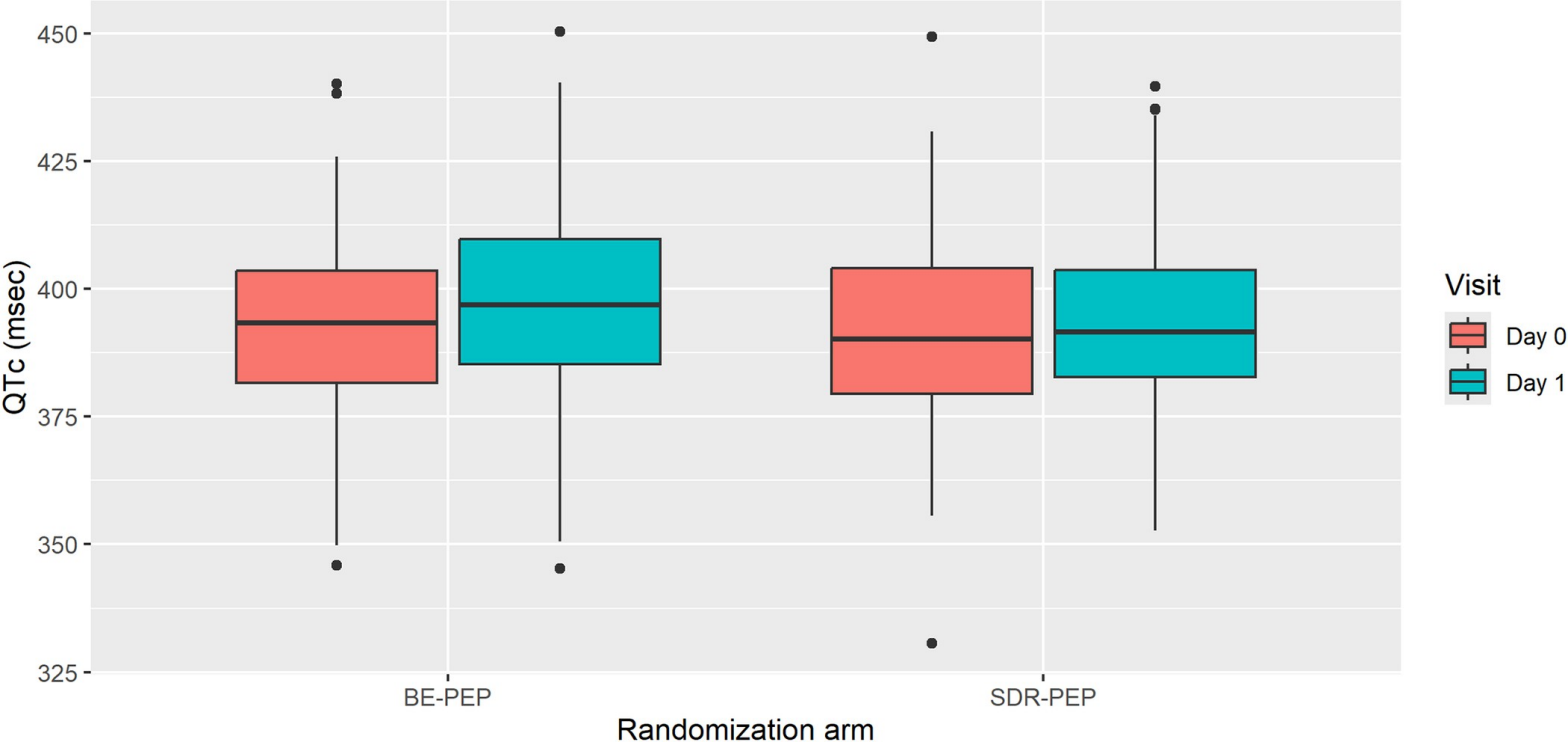
Box plots of QTc values on 12-lead ECG on day 0 (pre-drug administration) and day 1 (post-drug administration) by allocation arm for all ages. The bold black line depicts the median QTc measurement and the lower and upper sides of the box the interquartile range. Values over 1.5 times the interquartile range, over the 75th percentile or below the 25th percentile were presented as outlier points.

In the BE-PEP group, 150 participants (88 adults and 62 children under 18 (18 children aged 13 to 17 and 44 children aged 5 to 12)) had ECGs recorded both at baseline and after receiving BE-PEP, and were included in the per protocol analysis, versus 146 participants in the SDR-PEP group (Fig 2). Across all ages, the mean QTc changed from 393 ms before to 396 ms after BE-PEP, which was not different from the change from 392 ms before to 394 ms after SDR-PEP (difference between arms 1.8 ms, 95% CI −1.8–5.3, *p* = 0.41, Fig 3 and Table 2).

**Table 2:** ECG results (QTc) in ms by allocation arm and age-group in per protocol analysis.

Age-group	Day	BE-PEP Mean (95% CI; min, max)	SDR-PEP Mean (95% CI; min, max)	Difference (BE-PEP–SDR-PEP; 95% CI)	*P*-value for non-inferiority*
**All ages**	0	393 (390, 395; 346, 440)	392 (389, 395; 331, 449)	0.8 (−2.6, 4.2)	
*n* = 296	1	396 (393, 399; 345, 450)	394 (391, 397; 353, 440)	1.8 (−1.8, 5.3)	
*Regression analysis*					
Effect of BE-PEP on QTc (unadjusted for baseline QTc values)	Day 1	1.78(−2.45–6.01)	0 (Ref)		<0.001
Effect of BE-PEP on QTc (adjusted for baseline QTc values)	Day 1	1.78(−1.31–4.87)	0 (Ref)		<0.001
**Adults (≥18)**	0	395 (391, 399; 346, 440)	395 (391, 399;331, 449)	−0.3 (−6, 5.4)	
*n* = 173	1	397 (393, 401; 345, 440)	397 (393, 401;353, 440)	−0.2 (−6.1, 5.7)	
*Regression analysis*					
Effect of BE-PEP on QTc (unadjusted for baseline QTc values)	Day 1	−0.19(−6.11–5.73)	0 (Ref)		<0.001
Effect of BE-PEP on QTc (adjusted for baseline QTc values)	Day 1	0.78 (−3.36–4.93)	0 (Ref)		<0.001
**Children**	0	390 (386, 394; 362, 426)	387 (383, 391; 356, 426)	2.4 - (−2.9, 7.7)	
*n* = 123	1	395 (391, 399; 351, 450)	391 (387, 395; 353, 435)	4.5 (−1.3, 10.4)	
*Regression analysis*					
Effect of BE-PEP on QTc (unadjusted for baseline QTc values)	Day 1	4.51 (−1.33–10.36)	0 (Ref)		0.033
Effect of BE-PEP on QTc (adjusted for baseline QTc values)	Day 1	3.29 (−1.42–8.01)	0 (Ref)		0.003

*This p-value tests the (unilateral) hypothesis that BE-PEP causes more QTc prolongation than SDR-PEP; a value below 0.025 means that this hypothesis is rejected. BE-PEP = post-exposure prophylaxis (PEP) with bedaquiline 800 mg + rifampicin 600 mg; SDR-PEP = PEP with rifampicin 600 mg only, ms = milliseconds. Regr coeff = Regression coefficient, Ref = reference.

**Fig 4:**
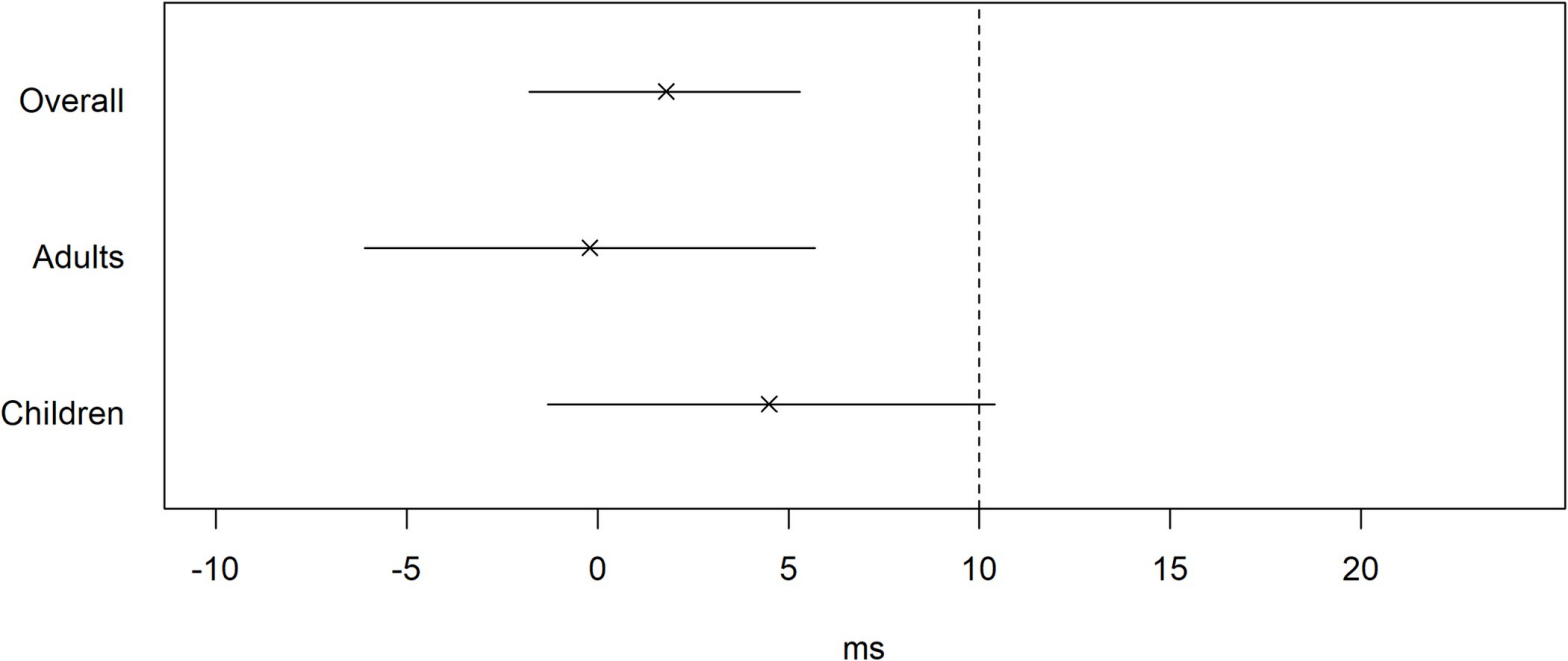
Estimated difference in ECG in milliseconds (BE-PEP (bedaquiline 800 mg + rifampicin 600 mg)—SDR-PEP (rifampicin 600 mg)) and 95% confidence intervals by age-group. The vertical dotted line indicates the 10 ms non-inferiority margin.

In adults, there was no difference in QTc after BE-PEP relative to SDR-PEP (−0.2 ms, 95% CI −6.1, 5.7, Table 2), meeting the non-inferiority margin of 10 ms (Fig 4). In children who received BE-PEP, the QTc increased from 390 ms to 395 ms, and in children who received SDR-PEP from 387 to 391 ms, with a difference between arms of 4.5 ms (95% CI −1.3, 10.4, Table 2). As the upper limit of the 95% CI of 10.4 ms exceeded the 10 ms non-inferiority margin, non-inferiority could not be established in children (Fig 4). Correcting for baseline QTc measurements, the average QTc measurements at day 1 in the BE-PEP group were 0.8 ms (95% CI −3.4, 4.9) higher among adults and 3.3 ms (95% CI −1.4, 8.0) higher among children than in the SDR-PEP group. Adjusting for the baseline QTc measurements, both results lie entirely below the 10 ms non-inferiority margin. The largest increase in QTc occurred in a child aged 13 to 17 in the BE-PEP group with a baseline QTc of 407 ms, who had a QTc of 450 ms post exposure. No individual’s QTc increased by >50 ms or exceeded 450 ms after PEP administration.

During the recruitment of adults, baseline levels of ALT and AST enzymes were found to be higher than anticipated (S1 and S2 Figs). Internal quality controls yielded acceptable results, but External Quality Assurance (Wales External Quality Assessment Services (WEQAS, https://www.weqas.com/), UK) identified inaccurate (too high) values in the normal range, while correct results were obtained for quality control samples in the elevated ranges. This was traced back to a technical error which was subsequently rectified. No significant difference in ALT or AST levels was noted between the control and intervention arms (S2 Table).

In the BE-PEP group, one participant was found to be pregnant at final follow-up on day 30, with last menstrual period estimated at 12 days before study drug administration. Severe hydramnios followed by a macerated stillbirth, with a knot in the umbilical cord, was considered unlikely to be related to bedaquiline nor rifampicin on review by an obstetrician and the DSMB. In the SDR-PEP group, one diabetic participant was admitted overnight for diabetic decompensation, presenting with trembling, nausea, and hyperglycemia 10 days after study drug ingestion, which was also considered unlikely to be related to rifampicin (Table 3). Certain AEs, including dizziness, nausea, headache, and diarrhea among adults, and headaches in children aged 13 to 17, were more frequently observed in the intervention group, although these differences were not statistically significant (Tables 3 and 4). The participant in the BE-PEP arm who immediately vomited study drugs after ingestion was excluded from per protocol analysis.

**Table 3:** Summary of safety analysis by age-group.

Age-group		BE-PEP *n* (%; 95% CI)	SDR-PEP *n* (%; 95% CI)	*p*-value*
**All ages**	Serious AE	1 (0.6; 0.1, 3.5)	1 (0.6; 0.1, 3.5)	1
	Drug-related AE	62 (39.5; 32.2, 47.3)	45 (28.8; 22.3, 36.4)	0.06
	Any AE	65 (41.4; 34, 49.2)	50 (32.1; 25.2, 39.7)	0.10
**Adults (≥18)**		*n* = 93	*n* = 94	
	Serious AE	1 (1.1; 0.2, 5.8)	1 (1.06; 0.19, 5.78)	1
	Drug-related AE	53 (57.0; 46.9, 66.6)	41 (43.6; 34.0, 53.7)	0.08
	Any AE	55 (59.1; 49.0, 68.6)	45 (47.9; 38.1, 57.9)	0.14
**Children**		*n* = 64	*n* = 62	
	Serious AE	0 (0; 0, 5.7)	0 (0; 0, 5.8)	1
	Drug-related AE	9 (14.1; 7.6, 24.6)	4 (6.5; 2.5, 15.4)	0.24
	Any AE	10 (15.6; 8.7, 26.4)	5 (8.1; 3.5, 17.5)	0.27

* Fisher’s exact test was used to compare the association between each type of safety event and allocation group.

AE, adverse event; *n*, number of participants; CI, confidence interval.

**Table 4:** Participant counts* with drug-related adverse events by preferred term and body system.

		BE-PEP *n* (%)	SDR-PEP *n* (%)
Adults (≥18)		*n* = 93	*n* = 94
	**Any drug-related AE**	**53 (57.0)**	**41 (43.6)**
	Cardiac disorders	3 (3.2)	1 (1.1)
	Palpitations	3 (3.2)	1 (1.1)
	Ear and labyrinth disorders	12 (12.9)	8 (8.5)
	Vertigo	12 (12.9)	8 (8.5)
	Gastrointestinal disorders	18 (19.4)	4 (4.3)
	Abdominal pain	1 (1.1)	0
	Diarrhoea	6 (6.5)	1 (1.1)
	Nausea	11 (11.8)	3 (3.2)
	Vomiting	1 (1.1)	1 (1.1)
	General disorders and administration site conditions	1 (1.1)	0
	Malaise	1 (1.1)	0
	Musculoskeletal and connective tissue disorders	1 (1.1)	0
	Arthralgia	1 (1.1)	0
	Nervous system disorders	11 (11.8)	4 (4.3)
	Headache	10 (10.8)	4 (4.3)
	Tremor	1 (1.1)	0
	Respiratory, thoracic, and mediastinal disorders	1 (1.1)	0
	Cough	1 (1.1)	0
	Investigations^#^	30 (32.3)	33 (35.1)
	Alanine aminotransferase increased	14 (15.1)	21 (22.3)
	Aspartate aminotransferase increased	22 (23.7)	21 (22.3)

**Children**		***n* = 64**	***n* = 62**
	**Any drug-related AE**	**9 (14.1)**	**4 (6.5)**
	Cardiac disorders	1 (5.0)	0
	Palpitations	1 (1.6)	0
	Ear and labyrinth disorders	2 (3.1)	1 (1.6)
	Vertigo	2 (3.1)	1 (1.6)
	Gastrointestinal disorders	2 (3.1)	1 (1.6)
	Abdominal pain	1 (1.6)	0
	Diarrhoea	1 (1.6)	0
	Nausea	0	1 (1.6)
	Vomiting	0	1 (1.6)
	Nervous system disorders	7 (10.9)	1 (1.6)
	Headache	7 (10.9)	1 (1.6)
	Investigations^#^	0	1 (1.6)
	Alanine aminotransferase increased	0	1 (1.6)

*Each person is counted only once, even if they might have had multiple AEs.

^#^ ALT or AST ≥ 2.5× upper limit of normal.

AE, adverse event; *n*, number of participants.

The determination of the baseline frequency of ALT and AST elevations in the population, a secondary objective, was complicated by the technical error in ALT/AST measurements leading to low-level elevations during recruitment of adults. Four of 261 adults were ineligible on the basis of hepatic function screening (Fig 2). Among children no baseline ALT/AST elevations were observed.

Of 261 screened adults, 2 were ineligible due to baseline QTc of 451 ms and 480 ms, i.e., 0.76% (95% CI 0.093%, 2.74%) with QTc >450 ms. These values were verified during the screening process by the referent qualified ECG reader. Given the low-level QTc elevations, these persons were not referred for further assessment. Among children no baseline QTc elevations >450 ms were observed.

## Discussion

In individuals who have been exposed to *M*. *leprae*, the single-dose administration of 800 mg bedaquiline and 600 mg rifampicin as PEP was found to be as safe as a single dose of 600 mg rifampicin alone in this open label randomized non-inferiority trial. Non-inferiority in children was however not demonstrated in unadjusted analysis, as the upper limit of the 95% CI of 10.4 ms exceeded the non-inferiority margin of 10 ms. Adjusting for the baseline QTc measurements the results in children were entirely below the 10 ms non-inferiority margin. We speculate that the observed unadjusted prolongation of the average QTc of 5 ms in children after BE-PEP, relative to the 4 ms prolongation after SDR-PEP, is unlikely to have clinical implications, and no clinically relevant prolongations nor clinical events occurred among participants. For the treatment of rifampicin-resistant TB, in which bedaquiline is combined with other QTc prolonging drugs like moxifloxacin and clofazimine for a treatment duration of 6 months or more, an increase in QTc >60 ms, and/or a QTc value >500 ms, is considered justification for intensive monitoring and treatment adjustment [19]. The transient side effects noted in the group who received the intervention are consistent with the bedaquiline product information, which identifies nausea and headache as possible adverse events. Given the global experience with bedaquiline for treatment of rifampicin-resistant TB, we expect these findings to be generalizable to other populations.

The findings of this study led to the positive advice of the DSMB, and subsequent ethical approval, to proceed with Phase 3 efficacy testing, currently comparing efficacy of the same 2 regimens in the highly leprosy endemic Comoros islands of Anjouan, where Phase 2 took place, and Mohéli. Given the low frequency of baseline QTc prolongations at the conservative cut-off of 450 ms (1.1% of adults, no children), and 3 ms prolongation observed after BE-PEP, in Phase 3 monitoring of QTc was not deemed necessary before giving PEP.

A highly effective PEP regimen would likely be a game-changer for leprosy control worldwide. In combination with active case finding in areas known to be leprosy endemic, which interrupts transmission by early case detection and treatment initiation, protection of contacts by highly effective PEP has the potential to lower the currently stable global incidence.

Our finding that a single bedaquiline 800 mg dose, in combination with rifampicin 600 mg, is safe in a healthy leprosy exposed population may have wider applicability. For treatment of TB, higher doses of bedaquiline may be needed to overcome resistance, although people with TB are usually considerably sicker than those with leprosy, and likely to be at greater risk of adverse events, so more data from this specific population are needed. Bedaquiline moreover has efficacy for other mycobacteria, including *M*. *avium*, for which a randomized clinical trial is recruiting (NCT04630145).

A limitation of this study was the inaccurately high test results in adults whose transaminases were in the lower ranges, before root cause analysis allowed to correct the testing procedure. As the External Quality Assessment displayed valid results for higher values, and no notable difference was seen between the intervention and control groups (S2 Table), it is improbable that a clinically relevant difference in liver toxicity among the adult participants has been overlooked. Mild elevations in liver enzyme tests in adults can however not be excluded, and the 4 adults who were excluded from enrollment based on baseline ALT/AST elevation may have been eligible had testing been accurate. Moreover, we cannot exclude that any QTc prolongation would have maximized beyond 24 h. The missing QTc values on D1 for 10 participants may furthermore have introduced bias in effect estimation, although excluding these from analysis did not affect the power of our hypothesis testing.

Furthermore, there is a concern that widespread use of rifampicin and bedaquiline as PEP may compromise their effectiveness for treatment of leprosy and TB patients. Selection for resistance after a single dose is considered unlikely. As a precaution, WHO guidelines recommend limiting rifampicin PEP to those who have not received any rifampicin in the last 2 years [18]. For rifampicin, a consensus statement was issued [4], and no mutations in *rpoB* were detected in *M*. *leprae* DNA from patients who developed leprosy after PEP to date [5]. For bedaquiline, with a longer half-life, this needs to be monitored. In the Phase 3 BE-PEOPLE trial all patients diagnosed with leprosy and TB on all 3 islands of the Union of the Comoros will be enrolled in a prospective cohort in which prior use of PEP is assessed. They will have baseline samples analyzed for the earliest indicators of drug resistance to rifampicin and bedaquiline using the Deeplex MycTB [20] and MycLep [5, 21] assays, modified to include *atpE*, the target of bedaquiline (Genoscreen, FR).

In conclusion, from a safety perspective a single administration of BE-PEP (800 mg bedaquiline + 600 mg rifampicin) is non-inferior to SDR-PEP (600 mg rifampicin alone) for individuals exposed to *M*. *leprae*. The efficacy of this regimen in reducing the incidence of leprosy is now being assessed in a Phase 3 cluster randomized trial (BE-PEOPLE; clinicaltrial.gov/NCT05597280).

## Supporting information

S1 CONSORT Checklist Checklist on compliance with CONSORT guidelines. (PDF)

S1 Safe list Guidance for managing concomitant medications. (DOCX)

S1 Protocol Study protocol; version in use at the time of the Phase 2 study. (PDF)

S1 Table ECG results (QTc) in ms by allocation arm and age-group in Intention to treat analysis. *This *p*-value tests the (unilateral) hypothesis that BE-PEP causes more QTc prolongation than SDR-PEP; a value below 0.025 means that this hypothesis is rejected. BE-PEP = post-exposure prophylaxis (PEP) with bedaquiline 800 mg + rifampicin 600 mg, SDR-PEP = PEP with rifampicin 600 mg only, ms = milliseconds. Regr coeff = Regression coefficient, Ref = reference.
(DOCX)

S2 Table ALT and AST by allocation arm, time point, and age-group. (DOCX)

S1 Fig Baseline distribution of ALT in screened individuals. (DOCX)

S2 Fig Baseline distribution of AST in screened individuals. (DOCX)

## Abbreviations:

ECG: electrocardiogram
DSMB: data safety and monitoring board
MDT: multidrug therapy
PEP: post-exposure prophylaxis; SAE, serious adverse event
SDR: single-dose rifampicin
TB: tuberculosis
ULN: upper limit of normal
WHO: World Health Organization
