# Supplementary material for: Safety of single-dose bedaquiline combined with rifampicin for leprosy post-exposure prophylaxis: A Phase 2 randomized non-inferiority trial in the Comoros Islands
Source: PLoS Med. 2024 Oct 21;21(10):e1004453. doi: 10.1371/journal.pmed.1004453 (PMC11534270; doi:10.1371/journal.pmed.1004453)
Supplement: S1 Safe list — (DOCX) [file pmed.1004453.s002.docx]

**How to manage concomitant medications :**

When using certain medications, individuals should not receive post-exposure prophylaxis (PEP) for bedaquiline. We have listed some of them here, and this list will be revised regularly.

Taking PEP with bedaquiline **is not permitted** for people who use:

- Any injectable drugs
- Any intravenous infusion
- Any antiretroviral drug (ending on or containing 'avir'), e.g. Efavirenz, Lopinavir, Ritonavir, Saquinavir, Atazanavir, Tenofovir disoproxil fumarate, Indinavir, Darunavir, Nelfinavir
- Diuretics: Furosemide (Lasilix), Hydrochlorothiazide (ESIDREX or HYZAAR)
- Treatment of opportunistic infections: pentamide, clarithromycin, azithromycin (Zithromax), azoles (e.g. Ketoconazole, Itraconazole, Levoketoconazole, Voriconazole).
- Anti-infective agents: erythromycin (Ery), halofantrine, Ciprofloxacin, (CIFLOX), Ofloxacin (OFLOCET)
- Anti-tuberculosis/anti-leprosy drugs: Moxifloxacin, gatifloxacin, levofloxacin, clofazimine (Lamprene), delamanid, capreomycin, amikacin, kanamycin
- Gastrointestinal medication: domperidone, metoclopramide (Primperan), ondansetron, loperamide >8mg/day
- Central nervous system drugs: citalopram, escitalopram, amitriptyline, chlorpromazine, haloperidol, thioridazine, methadone, Mifepristone, Clomipramine
- Cardiac medications: sotalol, amiodarone, digitalis therapy
- Antiepileptic drugs: Carbamazepine (Tegretol), Phenobarbital (GARDENAL), Phenytoin, Phosphenytoin
- Primidone
- Simvastatin (ZOCOR)

The list below contains drugs that are not contraindications to post-exposure prophylaxis with rifampicin and bedaquiline. In the event that a participant is using an oral medication that is neither on this list nor on the list of unauthorized medications, please consult Dr. Younoussa Assoumani before providing post-exposure prophylaxis.

The use of external products such as creams and ointments, eye drops, ear drops, or nose drops is not a contraindication to the use of post-exposure prophylaxis.

**List of permitted drugs :**

| **Generic name** | **Specialty name** | **Remarks** |
| --- | --- | --- |
| **I ORAL PRODUCTS** |  |  |
| Ascorbic acid | VITAMIN C | tablet |
| Folic Acid | SPECIAFOLDINE | tablet |
| Albendazole/mebandazole |  |  |
| Aminophylline | THEOPHYLLINE | tablet |
| Amlodipine | AMLOR | tablet |
| Amoxicillin + Clavulanic Acid | AUGMENTIN | oral suspension or tablet. |
| Amoxicillin | CLAMOXYL | oral suspension or tablet. |
| Ampicillin | TOTAPEN | capsule |
| Atenolol, | TENORMINE | tablet |
| Betamethasone 0.05 | CELESTENE | Drinkable tablet |
| Bisoprolol fumarte | CARDENSIEL | tablet |
| Bromazepam | LEXOMIL | tablet |
| Butylscopolamine | BUSCOPAN | tablet |
| Calcium Lactate | CALCIUM | tablet |
| Captopril tablet | LOPRIL |  |
| Carbocisteine | BRONCATHIOL | syrup |
| Cefixime | OROKEN | susp buv or tablet |
| Cefalexin | KEROFAL | tablet |
| Cetrizine dihychloride | ZYRTEC | gtte buv |
| Absorbent coal | CARBOPHOS | tablet |
| Tropatepine hydrochloride |  |  |
| Chlorpheniramine | POLARAMINE | tablet or syrup |
| Ciprofloxacin |  |  |
| Clomiphene Citrate | CLOMID |  |
| Cloxacillin | ORBENINE | tablet |
| Desloratadine | AERIUS | tablet or syrup |
| Dextromethorphan 10mg + Phenylnephrine 5 mg + Chlorphenamine | GRT sirp |  |
| Diazepam | VALIUM | tablet |
| Diclofenac Sodium | VOLTARENE | tablet |
| Doxycycline | VIBRAMYCIN | capsule |
| Fenofibrate | LIPANTHYL | capsule |
| Iron syrup | FUMAFER | syrup |
| Iron sulfate | FUMFER | tablet |
| Glibenclamide | DAONIL | tablet |
| Glicazide Retard | DIAMICRON | tablet |
| Griseofulvin | GRISEFULINE | tablet |
| Liquid Paraffin Oil |  |  |
| Aluminum hydroxide | ANTIACID | tablet |
| Hydroxyzine hydrochloride | ATARAX | tablet |
| Ibuprofen | ADVIL | syrup |
| Ibuprofen | ADVIL | tablet |
| Indomethacin | INDOCID | capsule |
| Irbesartan |  |  |
| Levamisole |  |  |
| Levodopa/Benserazide |  |  |
| Loratadine | CLARITYNE | tablet |
| Losartan Potassium | COZAAR | tablet |
| Metformin | GLUCOPHAGE | tablet |
| Methyl dopa | ALDOMET | tablet |
| Metronidazole | FLAGYL | tablet or oral suspension |
| Multivitamin | ALVITYL | tablet or syrup |
| Nifedipine LP | ADALATE | tablet |
| Omeprazole | MOPRAL | tablet |
| Paracetamol | DOLIPRANE | tablet or syrup |
| Paroxetine hydrochloride | DEROXAT | tablet |
| Prednisolone | SOLUPRED | tablet |
| Pyrimethamine and sulfadoxine | Fansidar |  |
| Ramipril | TRIATEC |  |
| Salbutamol | VENTOLINE | tablet or syrup |
| Oral rehydration salts, | ORS |  |
| Sildenafil citrate | VIAGRA |  |
| Spiramycin | ROVAMYCINE | tablet |
| Valsartan | DIOVAN | tablet |
| Spiramycin | ROVAMYCINE | tablet |
