# Supplementary material for: Safety of single-dose bedaquiline combined with rifampicin for leprosy post-exposure prophylaxis: A Phase 2 randomized non-inferiority trial in the Comoros Islands
Source: PLoS Med. 2024 Oct 21;21(10):e1004453. doi: 10.1371/journal.pmed.1004453 (PMC11534270; doi:10.1371/journal.pmed.1004453)
Supplement: S1 Protocol — (PDF) [file pmed.1004453.s003.pdf]

Clinicaltrials.Gov. NCT05406479 (Phase 2)/NCTXXXXXXXX (Phase 3)

Bedaquiline Enhanced Post ExpOsure Prophylaxis for Leprosy (BE-PEOPLE)

Initial Protocol
Version 4.0, 29-September-2022

Sponsor: Institute of Tropical Medicine
Nationalestraat 155
2000 Antwerpen
Belgium

Coordinating Investigator: Dr. Epco Hasker

**ClinicalTrials.Gov.:**	NCT05406479 (Phase 2) / NCTxxxxxxxx (Phase 3)
**Study Title:**	Bedaquiline Enhanced Post ExpOsure Prophylaxis for Leprosy (BE-PEOPLE)
**Study Acronym**	BE-PEOPLE
**Version and date:**	Version 4.0 29-September-2022
**Partnering institutions/sites:**	- Institute of Tropical Medicine (ITM), Antwerp, Belgium<br>- Damien Foundation (DF), Comoros and Belgium
**Study drugs:**	Bedaquiline and Rifampicin
**Funder:**	Janssen Pharmaceutica
**Sponsor:**	Institute of Tropical Medicine, Antwerp, Belgium
**Coordinating Investigator:**	Dr Epco Hasker<br>Institute of Tropical Medicine - Department of Public Health

Task division by collaborating partners:

- **Institute of Tropical Medicine**

Bouke de Jong	Trial coordination<br>Molecular testing samples Comoros	bdejong@itg.be, +32(0)32476590
Epco Hasker	Trial coordination	ehasker@itg.be, +32(0)32470726

- **National Tuberculosis and Leprosy Control Program of the Comoros**

Aboubacar Mzembaba	Supervise project activities in Comoros	a.mzembaba@gmail.com

- **Damien Foundation**

Younoussa Assoumani	Coordinate project activities in Comoros	yaoussoumani@gmail.com

- **Leidsch Universitair Medisch Centrum**

Annemieke Geluk	Process anti-PGL1 RDT strips	A.Geluk@lumc.nl

- **Amsterdam Universitair Medische Centra**

Christian van der Werf	Support ECG monitoring in phase 2 study	c.vanderwerf@amsterdamumc.nl

STATEMENT OF COMPLIANCE TEST

By signing this protocol, the Investigator(s) acknowledge(s) and agree(s):

This protocol contains the necessary information for conducting this clinical study. The Principal Investigator will conduct this study as detailed herein and will make every reasonable effort to complete the study within the time designated. The Principal Investigator commits to carry out the study in compliance with the protocol, amendments, applicable procedures and other study-related documents provided by the Sponsor, and in compliance with the Declaration of Helsinki, Good Clinical [Laboratory] Practice (GC[L]P), the ALLEA Code of Conduct for Research Integrity, and applicable regulatory requirements.

The protocol and all relevant study information, which is provided by the Sponsor, will be made available to the physicians, nurses and other personnel who participate in conducting this study. The Investigator will use this material for their training so that they are fully informed regarding the drugs and the conduct of the study.

The Sponsor of this study – the Institute of Tropical Medicine in Antwerp, Belgium (ITM) – will at any time have access to the source documents from which Case Report Form information may have been generated and will be permitted to perform trial-related monitoring and audits. All study material will be maintained according to regulatory requirements and until the Sponsor advises that retention is no longer necessary.

<u>DIRECTOR OF THE INSTITUTE OF TROPICAL MEDICINE:</u>

Title, Name: Dr. Marc-Alain Widdowson Date:

Signed:

<u>PRINCIPAL INVESTIGATOR:</u>

Title, Name: Dr. Epco Hasker Date:

Signed:

<u>PRINCIPAL INVESTIGATOR ON THE COMOROS:</u>

Title, Name: Dr. Younoussa Assoumani Date:

Signed:

Signing this document, I commit to carry out the trial in accordance with the protocol, Good Clinical Practice and applicable ethical and regulatory requirements. I also acknowledge the paragraph relevant to study confidentiality and authorize the Institute of Tropical Medicine, Antwerp, Belgium to record my data on a computerized system containing all the data pertinent to the study.

TABLE OF CONTENTS

STATEMENT OF COMPLIANCE TEST **3**
TABLE OF CONTENTS **5**
SYNOPSIS **7**
1.1 BACKGROUND 9
1.2 RATIONALE 11
2. STUDY OBJECTIVES AND OUTCOMES **12**
2.1 PHASE 2 STUDY 13
2.1.1 Primary: *13*
2.1.2 Secondary *13*
2.2 PHASE 3 STUDY 13
2.2.1 Primary *13*
2.2.2 Secondary *13*
3.1 GENERAL STUDY DESIGN 14
4. PARTICIPANTS, POPULATION & SELECTION **19**
4.1 STUDY SETTINGS 19
4.2 STUDY POPULATION AND SELECTION CRITERIA 19
4.3 SAMPLE SIZE 21
4.4 RANDOMIZATION 22
4.5 WITHDRAWAL AND TERMINATION OF THE STUDY 23
4.5.1 Reasons for Withdrawal from the study *23*
4.5.2 Reasons for Withdrawal from the study intervention *23*
4.5.3 Handling of Withdrawals *23*
4.5.4 Lost to follow-up *23*
4.5.5 Termination of Study *23*
5.1 GENERAL STUDY PROCEDURES 24
5.2 LABORATORY- AND SAFETY MONITORING PROCEDURES 27
5.2.1 Phase 2 pharmacovigilance: monitoring for hepatitis *27*
5.2.2 Sampling schedule for clinically confirmed leprosy patients *27*
5.2.3 Sampling of presumptive TB patients *28*
5.2.4 Specific tests *28*
5.2.5 Shipment and storage of samples *29*
6. STUDY INVESTIGATIONAL PRODUCT **30**
6.1 PURCHASING, PREPARATION AND ADMINISTRATION 30
6.2 PARTICIPANT COMPLIANCE MONITORING 30
6.3 IMP PREPARATION AND ADMINISTRATION 30
6.4 PRIOR AND CONCOMITANT THERAPY 30
6.5 PACKAGING 31
6.6 RECEPTION, STORAGE, DISPENSING AND RETURN 31
7. SAFETY ASSESSMENT **31**
7.1 RATIONALE FOR SAFETY AND EFFICACY OF STUDY DRUGS 31
7.1.1 Safety of Rifampicin 600mg *31*
7.1.2 Expected efficacy of bedaquiline 800mg against M. leprae *32*
7.1.3 Safety of Bedaquiline 800mg *32*

7.1.4 Rationale for combining bedaquiline 800mg and rifampicin 600mg ... *33*

7.2 Safety monitoring ... 34
7.2.1 Safety monitoring for QT prolongation in Phase 2 ... *34*

7.2.2 Safety monitoring for hepatitis in Phase 2 ... *34*

7.2.3 (S)AE monitoring ... *34*

7.2.4 Special Reporting Situation ... *37*

7.2.5 Product Quality Complaints (PQC) ... *38*

7.3 Surveillance for selection for mycobacterial drug resistance ... 39
7.4 Other risks ... 39
8. STUDY MANAGEMENT ... 40
9. STATISTICAL ANALYSIS ... 40
9.1 Baseline characteristics ... 40
9.2 Baseline characteristics ... **Error! Bookmark not defined.**
9.3. Phase 2 study ... 41
9.3.1. Primary analysis ... *41*

9.3.2. Secondary analysis ... *41*

9.3. Phase3 study ... 41
9.3.1. Primary analysis ... *41*

9.3.2 Secondary analyses ... *41*

9.3.3 Interim analyses ... *42*

10. MONITORING AND QUALITY ASSURANCE ... 42
11. DATA MANAGEMENT ... 43
11.1 Data collection, handling and retention ... 43
11.2 Data security and confidentiality ... 44
11.3 Data protection and security ... 44
11.3 Filing and archiving ... 45
12. ETHICAL ISSUES ... 45
12.1 Ethics and regulatory review ... 45
12.2 Protocol amendments ... 45
12.3 Informed consent ... 46
12.4 Confidentiality ... 47
12.5 Potential Risks ... 47
12.6 Benefits ... 48
12.7 Compensation for participation ... 48
12.8 Insurance ... 48
13. DISSEMINATION OF RESULTS, INTELLECTUAL PROPERTY ... 49
14. ARCHIVING ... 49
15. REFERENCES ... 50
16. LIST OF ABBREVIATIONS ... 54
17. ANNEX 1: FAGG OPINION TO THE PROTOCOL ... 55

SYNOPSIS

This study will evaluate a combination of bedaquiline and rifampicin as post exposure prophylaxis (PEP) for leprosy in Comoros. It will be a follow-up to the PEOPLE trial on PEP with rifampicin, which is ending in 2022. We call this new trial the 'Bedaquiline Enhanced Post ExpOsure Prophylaxis for Leprosy' or 'BE-PEOPLE' trial. There will be two main study arms, a comparator arm based on the current WHO recommendation of providing a single dose of rifampicin (10 mg/kg) to close contacts of leprosy patients and an intervention arm in which this regimen will be reinforced with bedaquiline, 400 or 800 mg depending on weight, to be repeated once after four weeks for household contacts. The main study will be preceded by a phase 2 safety study. Leprosy patients diagnosed over the course of the study will be enrolled in a sub-study, which will include genotypic testing for dug resistance.

Single dose rifampicin post exposure prophylaxis (SDR-PEP) for leprosy has been recommended by WHO since 2018 to reduce the risk of leprosy for close contacts of leprosy patients. [1] In the PEOPLE trial there are four study arms to which villages on two islands of Comoros (Anjouan and Mohéli) and in one district of Madagascar have been randomly allocated. These villages had been preselected based on leprosy incidence over the period 2013-2017. In all four study arms, annual door-to-door surveys are being conducted covering entire villages. All permanent residents are invited for screening for leprosy. Leprosy patients detected (further referred to as 'index patients') are offered free treatment according to the international and country guidelines. In arm 1 no PEP is provided, which is the current standard of care in Madagascar and Comoros. In arms 2, 3 and 4 SDR-PEP is offered to different categories of participants aged 2 years and above who have not received rifampicin in the preceding 2-year period. In arm 2 only household contacts of index leprosy cases are eligible, in arm 3 anyone living within 100 meters of an index case is eligible (or the entire village if >50% are eligible), in arm 4 household contacts as well as neighborhood contacts within 100 meter and testing positive to a serological test (anti-PGL I) are eligible. Based on studies in both tuberculosis and leprosy that showed high doses of rifampicin to be safe and more effective than the standard dose of 10 mg/kg, in the PEOPLE trial we opted for a dose of 20 mg/kg and refer to this regimen as 'single double dose rifampicin post exposure prophylaxis' or SDDR-PEP. [2-5] The BE-PEOPLE trial will continue only in Comoros, where the highest leprosy incidence was found so far during the PEOPLE trial.

The total enrolment to date in the PEOPLE trial in Comoros has been approximately 86,000. Out of 15,569 persons eligible for PEP in 2019, 12,101 (83.1%) actually took PEP. A preliminary analysis based on data for the second year (2020) shows an incidence of approximately 1.1 per 1,000 (96 new cases). In a Poisson model controlled for differences in baseline prevalence and with village nested in island as random effects to control for contextual confounding, we found incidence rate ratios of 1.23, 0.78 and 0.89 when comparing respectively arms 2, 3 and 4 to arm 1, the comparator arm. None of these associations was statistically significant ($p \geq 0.70$). At individual level the effect of PEP controlled for distance to nearest index case was a borderline statistically significant reduction in risk of leprosy, with an incidence rate ratio of 0.48 (95% confidence interval 0.21-1.13).

Based on the individual effect of SDDR-PEP and taking into account the fact that less than two out of three years of follow-up have passed, the associations at study arm level might still become more pronounced but we do already want to test a stronger PEP regimen making use of the established set up. The regimen we intend to test is a combination of rifampicin and bedaquiline, hereafter referred to as 'Bedaquiline enhanced post exposure prophylaxis' or BE-PEP. Rifampicin will be provided at a dose of 10 mg/kg, the WHO standard, i.e. 600 mg for an adult. Bedaquiline will be provided at a dose of 800 mg for an adult, double the daily dose used in treatment of rifampicin resistant tuberculosis.[6] Based on pharmacological considerations and pre-clinical studies we expect this regimen to have a synergistic action against *M. leprae*. BE-PEP will be administered as a single dose, repeated after four

weeks only for household contacts, as preliminary data from the PEOPLE trial show that their residual risk is still up to three times higher when compared to the rest of the population.

In our analysis of baseline data of the PEOPLE trial we found strong degrees of clustering of leprosy not just within households but also in neighborhoods, with statistically significant associations up to 100 meters from an index case.[7] For this reason for the BE-PEOPLE trial we opted for a blanket approach, comparable to arm 3 of the PEOPLE trial. PEP will be provided to anyone living within a 100 meter radius of an index case (or to the entire village if more than 50% of the population would be eligible). An additional argument for the blanket approach is the fact that, as mentioned above, even though at individual level SDDR-PEP appears to protect reasonably well, the overall incidence during the first year of follow-up in the study arm targeting only household contacts (arm 2) was slightly higher than the incidence in the comparator arm. As stated earlier, this difference was by no means statistically significant but it appears to confirm the findings of Bakker e*t al.* in a high incidence setting in Indonesia where PEP was only effective when blanket coverage was provided.[8] Blanket coverage within a 100 meter radius has now also been recommended in a recent WHO guideline. [9]

During the PEOPLE trial we have created teams of dedicated field workers and supervisors and gained precious experience with electronic data capturing and georeferencing of our study population. For these reasons it is crucial not to let the capacity that has been built dissipate, and to start a follow-up trial without delay. The PEOPLE trial will end in 2022, after a fourth round of door-to-door screening in all four study arms. Out of 48 villages in Comoros that are part of the PEOPLE trial we will retain 30, based on incidence to date and population size. Based on prevalence during the 2022 survey, these will be randomized within island and PEOPLE study arm to the two arms of the BE-PEOPLE trial, along with ten additional villages. In the PEOPLE study, in 2022 nobody will have received rifampicin so all will eligible again for rifampicin in 2023.

Given the fact that we are going to provide to healthy people a drug that has not been used before for this indication and which has only been conditionally approved for use in multi-drug resistant tuberculosis, we have first foreseen a phase 2 study in which BE-PEP will be provided to a limited number of contacts and in which safety will be closely monitored and evaluated by an independent data and safety monitoring board (DSMB). This will be done in a small village that is part arm 1 of the PEOPLE trial in which 8 new cases have been diagnosed since 2019 but no PEP has been provided. We will conduct door-to-door screening in this village in June 2022 and offer a single dose of BE-PEP to a random sample of 150 people screened aged 5 years and above not meeting the exclusion criteria (active TB or leprosy or previously treated leprosy, known liver function or cardiac abnormalities, not able to swallow 100 mg bedaquiline tablets). Participants will be followed up closely with active monitoring for adverse events, including measurement of the corrected QT interval and liver function before and after administration, as well as gastro-intestinal (nausea, vomiting), nervous system-related (headache, dizziness) and cutaneous reactions. The remainder of the population of this village aged two years and above will be offered single dose rifampicin as per WHO recommendations. In a randomly sampled subset of 150 individuals receiving rifampicin only we will perform the same stringent monitoring with ECG and liver function tests also applied in those receiving BE-PEP.

Assuming the phase 2 study will not reveal any drug related adverse events and following the advice of the DSMB and the involved ethics committees, we will proceed with a phase 3 study for which randomization will take place in December, 2022. There will be two study arms. Arm 1 will be the intervention arm in which we will provide BE-PEP to all persons residing within 100 meters of an index case, to be repeated after four weeks for household contacts. Arm 2 will be the comparator arm in which the WHO recommended standard PEP will be provided, i.e. 10 mg/kg of rifampicin in a single dose. In both arms we will target anyone living within 100 meters of an index case or the entire village if more than 50% are eligible. Provision of BE-PEP will start in 2023 and follow-up will continue until 2026. Our main study outcome will be the comparison of leprosy risk in individuals that received

standard WHO SDR-PEP versus individuals that received BE-PEP. In addition we will compare the overall leprosy incidence over the follow-up period between the two study arms.

As stated above, our primary outcome measure will be the incidence rate ratio of leprosy between those who received BE-PEP and those who received the standard SDR-PEP. From this analysis we will exclude those not eligible for BE-PEP, i.e. children below 5 years of age and/or with a weight below 20 kg. We will fit a Poisson model with village nested in island as random effect and controlled for distance to the nearest index case at basline. In addition we will compute incidence rate ratios between the entire BE-PEP and SDR-PEP arms over the period 2023-2026, also based on a Poisson model with village nested in island as random effect.

Throughout the BE-PEOPLE trial we will continue sampling leprosy patients identified (a skin biopsy from the edge of non-facial lesions), with the aim of using molecular assays for *M. leprae* as quality assurance mechanism. If sufficient DNA is available Deeplex-MycLep will be used for typing the strains, which will allow us to perform highly sensitive surveillance for (traces of) resistance to rifampicin and bedaquiline. As part of BE-PEOPLE, we will moreover enroll all microbiologically confirmed tuberculosis patients on all islands of Comoros (Moheli, Anjouan, and Grande Comore, where bedaquiline will not be introduced, maximum 100 patients/ year) and assess whether they ever received (BE-)PEP or leprosy treatment. We will genotype their sputum with Deeplex-MycTB XL, which includes all *M. tuberculosis* genes (potentially) involved in resistance to rifampicin and bedaquiline, to be able to detect the earliest traces of acquired resistance to these drugs, which is unexpected after single dose administration.

The overall goal of BE-PEOPLE is to validate a robust and safe leprosy PEP regimen, and its optimal administration, that prevents leprosy in the individual and interrupts transmission at the village level. If BE-PEP turns out to be safe and successful, we aim to adjust national guidelines in Comoros towards island wide implementation on Anjouan and Moheli, allowing to sustainably eliminate leprosy.

1. INTRODUCTION

1.1 Background

Leprosy is an ancient infectious disease transmitted from man to man, probably via the airborne route. After contact with the causative microorganism (*M. leprae*) and before the onset of skin and nerve lesions, an infected person may be asymptomatic for years. [10-12] Post exposure prophylaxis (PEP) provided during this incubation period might prevent progression from infection to disease.[13] In 1991, the World Health Assembly decided on a leprosy elimination strategy aiming at reducing the prevalence to below 1 per 10,000 worldwide, assuming that at such levels transmission will eventually cease.[14] Leprosy prevalence has since then been greatly reduced, from more than 5 million in the 1980s to 129,192 by the end of 2020. However much of the reduction in prevalence has been due to changes in case definition and in treatment duration, the latter having been reduced from lifelong to maximum two years and then again to maximum one year. The decline in leprosy incidence has been less impressive. After a steep decrease between 2000 and 2005, reported worldwide leprosy incidence has now plateaued above 200,000 annually, showing continued transmission of *M. leprae*.[15] One of the measures endorsed by WHO in 2018 to overcome the current stalemate is post exposure prophylaxis based on a single dose of rifampicin (SDR-PEP) provided to close contacts of leprosy patients.[16] PEP based on a single dose of rifampicin has been shown to reduce the risk of leprosy by 50-60% in a pivotal trial in Bangladesh.[17]

The current study protocol is a sequel to the PEOPLE trial on post exposure prophylaxis for leprosy in Comoros (48 villages) and Madagascar (16 villages), funded by EDTCP, which in turn built on the ComLep cohort of leprosy patients on Anjouan (Comoros), 2017-2019, funded by R2Stop.[18] PEOPLE consists of a cluster randomized trial, which will end towards the end of 2022, in which three different approaches to post exposure prophylaxis for leprosy (PEP) are compared to an intervention arm in which no prophylaxis is provided. We opted for single dose rifampicin post exposure prophylaxis, as recommended by WHO. [1] But based on studies in both tuberculosis and leprosy that showed high doses of rifampicin to be safe and more effective than the standard dose of 10 mg/kg, in the PEOPLE trial we opted for a dose of 20 mg/kg and refer to this regimen as 'single double dose rifampicin post exposure prophylaxis' or SDDR-PEP. [19] Arm 1 is the comparator arm in which no PEP is provided, in arm 2 SDDR-PEP is provided to household contacts of index cases. In arm 3 SDDR-PEP is provided not only to household contacts but to anyone living within a 100 meter perimeter of an index case, if this entails more than 50% of the village population the entire village is eligible. In arm 4 SDDR-PEP is given to household contacts but also to those living within a 100 meter perimeter of an index case and testing positive to anti-PGL-I, an antibody detection test for *M. leprae*. If more than 75% of the village population lives within 100 meters of an index case, eligibility for anti-PGL-I positive non-household contacts is extended to the entire village. Door-to-door screening is performed on an annual basis in all villages. The main outcome measure will be the incidence rate ratios of leprosy between the comparator arm and each of the intervention arms.

During the first year of follow up 8 new cases were detected in Madagascar on a population screened of 19,039, an incidence of 4.2 per 10,000, versus 80 on 62,266 screened in Comoros, equivalent to an incidence of 13.2 per 10,000. On the total population under follow-up in Comoros of 86,160, 96 cases were detected, equivalent to an incidence of 11.1 per 10,000. In Comoros post exposure prophylaxis had been provided from the start, in Madagascar only from the second year onwards. It is because of the higher incidence that we have decided to focus on Comoros for the new trial described in this protocol. We have seen some impact of SDDR-PEP in Comoros but we would like to seize the opportunity to also test another potentially more efficacious PEP regimen. So far, based on just one year of follow up, a Poisson model controlled for differences in baseline prevalence and with village nested in island as random effects to control for contextual confounding, shows incidence rate ratios of 1.23, 0.78 and 0.89 when comparing respectively arms 2, 3 and 4 to arm 1, the comparator arm. None of these associations was statistically significant ($p \geq 0.70$) and based on data available to date for the third project year we already have a clear indication that the differences between the intervention arms and the comparator arm are not increasing. At individual level the effect of PEP over the first year of follow-up, controlled for distance to nearest index case at baseline, was a borderline statistically significant reduction in risk of leprosy, with an incidence rate ratio of 0.48 (95% confidence interval 0.21-1.13). The individual effect is in line with the results of the pivotal COLEP trial that showed 57% reduction in incidence, albeit COLEP used 600mg of rifampicin and enrolled close contacts only.[17] Though the 2018 WHO guideline is based on the individual effect of SDR-PEP, in a more recent guideline WHO has also included a blanket approach, typically covering an area of 100 meters around an index case. [9]

The available set-up and experience gained so far during the PEOPLE trial present an ideal platform for a follow-up study. In this follow-up trial which we have called 'Bedaquiline Enhanced Post ExpOsure Prophylaxis for Leprosy' or 'BE-PEOPLE', we will test a reinforced regimen made up of rifampicin and bedaquiline which we will hereafter refer to as BE-PEP. In the PEOPLE study, in 2022 nobody will have received rifampicin so all will eligible again for rifampicin in 2023. The main objective of the study is to assess effectiveness of BE-PEP in comparison to the standard WHO recommended SDR-PEP regimen. We will assess not only the individual protective effect but also the effect on incidence at village level.

1.2 Rationale

As was explained above, based on preliminary data for the first year of follow-up of the PEOPLE trial and taken into account differences in baseline prevalence, in Comoros we find incidence rate ratios of 1.23, 0.78 and 0.89 when comparing respectively arms 2, 3 and 4 to arm 1, the comparator arm. None of these associations was anywhere near statistically significant ($p \geq 0.70$) and already we observe that during the second year of follow-up differences between intervention arms and comparator arm are not increasing. Though we do start seeing an effect at individual level, a marginally significant reduction in risk of 52%, we hypothesize that a more effective PEP regimen is required to not just prevent individual progression from asymptomatic infection to disease but to have an impact on transmission as well.

The setup of the ongoing PEOPLE trial provides an ideal context to test an alternative PEP regimen. A strong data collection system has been developed and extensively field tested, survey teams have been trained and quality assurance mechanisms established. Important lessons have been learnt to which our system has been adapted. Whereas in Madagascar we found a relatively low incidence to date, approximately 4.2 per 10,000 in the first year of follow-up, in Comoros the overall incidence over the first year of follow-up was still 11.1 per 10,000 per year, despite SDDR-PEP being provided in three of the four study arms. Moreover there are some larger villages in Comoros that were not included in the PEOPLE trial out of concerns for an imbalance between study arms upon randomization, in these villages we continue to see high leprosy incidence, in the order of 20-30 per 10,000 per year over the past five years.

The PEP regimen we will test is a combination of bedaquiline and rifampicin. It will be provided as a single dose, to be repeated once after four weeks for household contacts because the PEOPLE study has unequivocally shown that they are most at risk, even after risk reduction with SDDR-PEP.[7] Rifampicin has been endorsed for post exposure prophylaxis of leprosy since 2018.[1] Bedaquiline, the first new drug to be developed against *M. tuberculosis* in 40 years, received conditional FDA approval in 2012 for treatment of multi-drug resistant tuberculosis. Bedaquiline targets the subunit c of the ATP synthase in the respiratory chain, and has become a 'game changer' in the treatment of tuberculosis (TB) patients with advanced resistance. Bedaquiline is typically given once daily for 2 weeks, followed by 3x weekly dosing for 6 months or longer. It has a very long half-life once steady state is established. In the clinical studies preceding bedaquiline approval, safety was established for a single supratherapeutic dose of 800 mg (see 7.1.4 below). In bedaquiline dose ranging studies in TB patients, the highest dose used consisted of a loading dose of 700mg bedaquiline followed by a dose of 500mg on day 2, which showed the strongest early bactericidal activity.[20] In vitro *M. tuberculosis* studies show that bedaquiline as well as rifampicin are active against both replicating and non-replicating bacteria, whereas moxifloxacin and isoniazid only kill replicating bacteria.[21] The combination of rifabutin, with the same mode of action as rifampicin, plus bedaquiline produced sustained intracellular mycobactericidal activity that was greater than the sum of their individual effects.[22] While bedaquiline is a substrate the cytochrome P450 isoenzyme CYP3A4, of which rifampicin is a strong inducer, a single dose as used for PEP is not expected to lead to drug-drug interactions [23].

To assess transmission of leprosy, the value of door-to-door surveys is limited because of the long incubation period, moreover from a cost perspective they are not sustainable. A possible alternative could be serological surveys among children, as is done for lymphatic filariasis.[24] For leprosy a serological test, anti-PGL-I, has been available for years but to date longitudinal data required for validation as a surveillance tool are lacking.[25] The available data from the PEOPLE trial, including age specific sero prevalence rates as well as leprosy prevalence rates in villages that belong to arm 4, provide a unique opportunity. At the end of the BE-PEOPLE trial in 2026 we will therefore conduct another anti-PGL-I sero survey in the original arm 4 villages of PEOPLE. We expect that as a result of nine years of active case finding combined with different forms of PEP, transmission of *M. leprae* in

these villages will have been substantially reduced. We want to use the 2026 PGL-I survey as a proof of principle to assess whether such surveys can be used as instruments to measure transmission. We expect that, especially in young children, anti-PGL-I positivity will have strongly and significantly declined.

While the concern that a single dose of rifampicin selects for resistance to rifampicin in leprosy and/or tuberculosis has been refuted on theoretical grounds, this has not been formally tested. Unpublished findings from the PEOPLE trial (manuscript in preparation) reveal the complete absence of (even subpopulations of) resistant *M. leprae* bacilli in Comoros, also in two patients who developed leprosy after having received rifampicin based PEP. To ensure continued monitoring for drug resistance in the BE-PEOPLE trial, we will continue to enroll in a sub-study all incident cases of leprosy from all villages of Grande Comore, Anjouan and Mohéli. From these patients we will collect skin biopsies from non-facial lesions to be tested with with qPCR and Deeplex-MycLep if sufficient DNA is available. Apart from testing just for rifampicin and fluoroquinolone resistance as we are currently doing, in BE-PEOPLE we will extend the genotyping of *M. leprae* with bedaquiline resistance conferring genes.

To date, Xpert MTB/RIF, implemented on Anjouan since 2018, has never detected a patient with rifampicin resistant tuberculosis. To further monitor resistance in tuberculosis in the wake of introduction of BE-PEP we will also enroll tuberculosis patients to test whether any rifampicin or bedaquiline resistant *M. tuberculosis* bacilli are detectable. This will be done on all three islands of Comoros, including Grande Comore where no BE-PEP will be provided.

To summarize, preliminary results of the PEOPLE trial indicate that to interrupt transmission of leprosy in all probability requires a stronger prophylactic regimen than the current SDDR-PEP, in particular in high endemicity environments such as Comoros. A combination of bedaquiline and rifampicin is expected to have a synergistic effect and could still be provided as a single dose, even if repeated once after four weeks for household contacts.[22] To achieve maximal impact of a single administration, safety and pre-clinical data support bedaquiline at 800mg. The available set up in Comoros, established for the PEOPLE trial, presents an ideal backdrop for a follow-up trial. In addition we will assess the potential of PGL-I sero surveys as a tool to measure transmission of *M. leprae* and we will closely monitor for any potential emergence of resistance against bedaquiline or rifampicin both in leprosy and in TB patients.

2. Study Objectives and Outcomes

The study is made up of two parts, a phase 2 study to reconfirm the safety of the trial regimen, i.e. the combination of bedaquiline and rifampicin administered as a single dose, and a phase 3 study in which we will evaluate effectiveness of this regimen in preventing leprosy, compared to the standard WHO regimen consisting of a single dose of rifampicin only. Implementation of the phase 3 study will be conditional upon the safety results of the phase 2 study. In addition will enroll leprosy patients diagnosed, as well as tuberculosis patients, to monitor resistance against the study drugs. We will also conduct a PGL-I sero survey in part of the study population for whom earlier PGL-I data are available to assess the potential of such surveys as a tool to monitor transmission of *M. Leprae*. These sub-studies are an integral part of BE-PEOPLE because we need to be able to monitor whether resistance against the drugs used, bedaquiline and rifampicin, is emerging, from the earliest possible stages. Also we need to be able to assess whether post exposure prophylaxis has an impact not only on progression from infection to disease but on transmission as well; this requires a tool that needs to be validated.

2.1 Phase 2 study

2.1.1 Primary:

To confirm safety of the study regimens, single dose bedaquiline plus rifampicin and single dose rifampicin, in a field setting in Comoros

Outcome: Advice by DSMB on whether or not phase 3 trial can be implemented as foreseen in this protocol, taken into account QTc data, observed (S)AEs and predetermined stopping criteria.

2.1.2 Secondary

1. To determine the baseline frequency of ALT and AST elevations and QTc prolongations in the population.
2. To document for each of the two regimens post administration QTc levels, as well as any potentially frequent adverse events such as gastro-intestinal (nausea, vomiting), nervous system-related (headache, dizziness) and cutaneous reactions.

2.2 Phase 3 study

2.2.1 Primary

To evaluate effectiveness of PEP based on a combination of rifampicin and bedaquiline (BE-PEP), in preventing leprosy among contacts of incident cases.

Outcome: The incidence rate ratio of leprosy between contacts who received the trial regimen (BE-PEP: rifampicin 600 mg plus bedaquiline 800mg, repeated once after four weeks for household contacts) and those who received the WHO standard prophylactic regimen (SDR-PEP: rifampicin 600 mg, single dose).

2.2.2 Secondary

1. To assess effectiveness of the BE-PEP regimen at village level.

   Outcome: The incidence rate ratio between villages that received BE-PEP and villages that received SDR-PEP.

2. To quantify frequency of potential adverse events such as gastro-intestinal (nausea, vomiting), nervous system-related (headache, dizziness) and cutaneous reactions.

   Outcome: The proportion of participants treated with BE-PEP that report adverse events, with a breakdown by type of event.

3. To assess anti-PGL-I sero surveys as a tool to monitor leprosy transmission

   Outcome: Age specific anti-PGL-I sero prevalence rates in villages belonging to arm 4 of the original PEOPLE trial at the time of the first survey round in 2019 and the final round of BE-PEOPLE in 2026

4. To monitor rifampicin and bedaquiline resistance among leprosy and tuberculosis patients

   Outcome: We will quantify the prevalence of rifampicin and/or bedaquiline resistant strains of *M. leprae* and *M. tuberculosis* on each of the study islands making use of molecular markers.

5. To assess cost-effectiveness of the BE-PEP regimen compared to SDR-PEP.

   Outcome: Cost per case averted between BE-PEP arm and SDR-PEP.

3. Study Design

3.1 General study design

We will conduct a phase 3 intervention trial comparing the effectiveness of two different regimens of post exposure prophylaxis for leprosy. Because we will be using a new drug (bedaquiline) that has not been used before for this indication, we have planned a phase 2 safety study to start in May, 2022. For this study we will select a small village (Gégé, Anjouan) that is part of the comparator arm of the PEOPLE trial and in which to date eight leprosy cases were diagnosed but that will not be included in the BE-PEOPLE trial because of its small population size (n=908). In this village, in May 2022, we will conduct the final round of door-to-door screening for leprosy in the framework of the PEOPLE trial. All cases identified will be treated in accordance with the national standards. Upon completion of screening, a random sample of 150 participants not meeting the exclusion criteria will be offered BE-PEP with strict monitoring for adverse events, in particular ECG and liver function will be monitored pre- and post-drug administration. The remainder will receive single dose rifampicin (SDR-PEP), as per WHO recommendation. Of those a random sample of 150 will also be subjected to the same procedures for monitoring adverse events as those receiving BE-PEP. Results will be presented to an independent data safety management board and the regulatory authority in the Comoros who will then decide whether or not the main phase 3 trial can go ahead. The full BE-PEOPLE study is expected to run from January, 2022 till December 31st, 2026. A Gantt chart of the main project activities is presented in figure 2 below.

The BE-PEOPLE study is a follow-up to the ongoing PEOPLE study, in Comoros only. Baseline screening will take place in 2022, partially overlapping with the final screening round of PEOPLE. We will then re-randomize 34 out of 48 villages that were already part of PEOPLE as well as 10 new clusters to two new study arms: arm 1 in which BE-PEP i.e. bedaquiline (400 or 800 mg depending on weight band) combined with rifampicin (10 mg/kg) will be provided as post-exposure prophylaxis, and arm 2 in which only SDR-PEP (rifampicin 10 mg/kg) will be provided. Anyone living within 100 meters of an index case and not meeting the exclusion criteria will be eligible for PEP. In case 50% of the village population or more will be eligible, we will provide PEP to the entire village population, conditional on meeting age and weight criteria. Any individual who received PEP will only become eligible again two years later, provided there has been continued exposure.

Eligibility in 2023 will be based on any index case detected in the preceding five-year period, i.e. since January 1st, 2018. Lists of eligible persons will be made up at the end of the baseline screening in 2022 and will be updated if further cases are detected or new participants enrolled during the 2023 screening round. From 2023 till 2025 there will be annual screening rounds, followed up with PEP intervention to those eligible, always respecting the two year interval (at least 20 months) between two PEP provisions. Because there will be no distribution of PEP during the final PEOPLE survey round in 2022, all participants will in principle be eligible again for PEP when the BE_PEOPLE intervention starts in 2023.

The final screening round will take place in 2026 and will in a subset of villages (those that belonged to arm 4 of PEOPLE) be followed by a serological survey (PGL-I) aimed at measuring prevalence of infection with *M. leprae*.

As we did in the PEOPLE trial, we will continue to enroll all leprosy patients who are detected island-wide on Grande Comore, Anjouan and Mohéli in a sub-study for which a separate informed consent will be asked. This also includes patients from outside BE-PEOPLE villages. For each consenting leprosy patient identified a skin biopsy is taken from the edge of a lesion, except if there are only facial lesions. These samples are subjected to qPCR and, if positive with a sufficiently high bacterial load, DNA is tested for mutations associated with resistance against bedaquiline, rifampicin, dapsone and

fluoroquinolones. [26] This sub-study is an integral part of BE-PEOPLE because without it we will not be able to monitor resistance to the study drugs. So far out of 1030 patients sampled in the PEOPLE trial, 755 (73.3%) were confirmed qPCR positive whereas none of the negative control samples that are concurrently collected in the field tested positive, indicative of the high quality of diagnostic procedures in the field. Out of 260 biopsy extracts that had sufficient DNA for processing with Deeplex Myc-Lep, none showed any indications of drug resistance, not even minority populations (S. Braet, manuscript in preparation). This component will continue as part of the quality assurance of the BE-PEOPLE trial as well as for monitoring emergence of drug resistance.

We will also enroll all tuberculosis patients from all villages on all three islands of Comoros, for surveillance of bedaquiline and/or rifampicin resistance on sputum samples and possibly tongue swabs. This sub-study too is an integral part of BE-PEOPLE because when distributing rifampicin and bedaquiline on large scale it is imperative to monitor for even the earliest signs of possible drug resistance emerging. The reason for including the third island, Grande Comore, where no bedaquiline will be administered, is to identify any baseline (minority) bacterial populations with mutations in gene *Rv0678*, implicated in low level bedaquiline resistance, as control group. Table 1 below provides and overview of the main activities over the project period.

**Year**	**Activity**				
2022	Phase 2 study	Baseline screening + randomization	Sampling leprosy patients	Sampling TB patients	
2023	Phase 3 study	Screening Arm 1 & 2 + PEP intervention	Sampling leprosy patients	Sampling TB patients	
2024	Phase 3 study	Screening Arm 1 & 2 + PEP intervention	Sampling leprosy patients	Sampling TB patients	
2025	Phase 3 study	Screening Arm 1 & 2 + PEP intervention	Sampling leprosy patients	Sampling TB patients	
2026	Phase 3 study	Final screening Arm 1 & 2	Sampling leprosy patients		PGL-I survey

Table 1: Time table BE-PEOPLE

Participants in the new villages will be asked for informed consent for baseline screening in 2022. Informed consent for the intervention trial will be asked during the first intervention round in 2023, when we know to which study-arm villages have been randomized. Informed consent for the leprosy patient cohort will remain separate, the same applies to the TB patients being enrolled. For the PGL-I survey in 2026, separate informed consent will be asked ahead of the survey.

Figure 1 below shows how villages belonging to the various arms of PEOPLE will merge into BE-PEOPLE. We have dropped some villages that had a low incidence so far during the PEOPLE trial or a small population size. To arrive at the required sample size we will also include nine new villages, eight on Anjouan and one on Mohéli, the latter to be split into two clusters. Villages will be arranged by PEOPLE study arm and by island in order of decreasing prevalence during the 2022 baseline survey and regrouped into pairs. Within each pair they will be randomized to arm 1 or arm 2 of BE-PEOPLE.

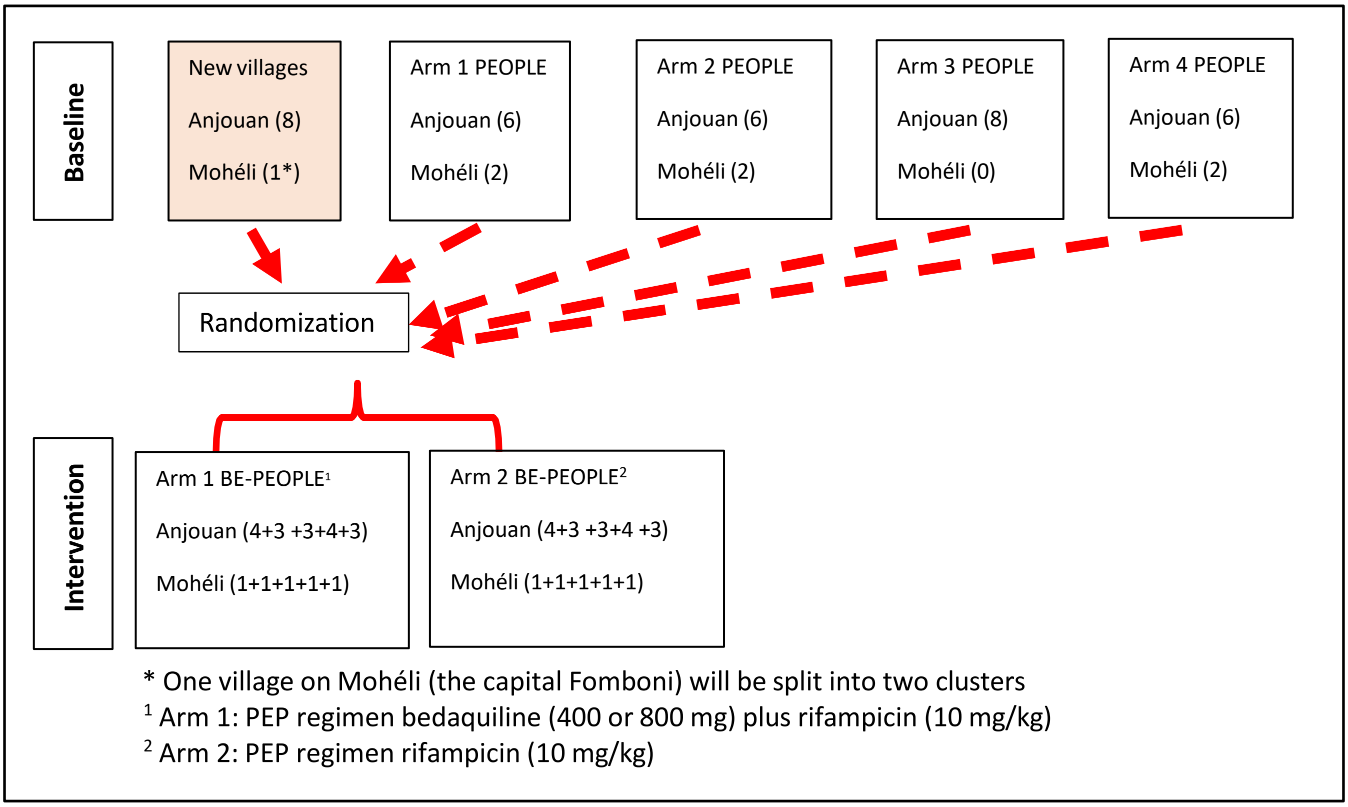

Figure 1: Inclusion and randomization BE-PEOPLE (Phase 3) trial

The main outcome measure of BE-PEOPLE will be the incidence rate ratio of leprosy between individuals in arm 1 who received BE-PEP and individuals in arm 2 who received SDR-PEP, controlled for potential confounding by distance to nearest index case at baseline, as well as for contextual confounding through the use of a random effects model (village nested within island). To avoid a potential selection bias, those who received SDR-PEP but would not have been eligible for BE-PEP, i.e. children below the age of 5 or with a weight below 20 kg, will be excluded from this analysis. Details are described in chapter 9, 'Data analysis'.

We will make optimal use of the setup created in the PEOPLE project with five survey teams on the island of Anjouan and two on the island of Mohéli that have recorded and georeferenced all study participants to date in the PEOPLE trial (> 85,000). In addition, on both islands there are central teams that supervise activities in the field, confirm leprosy suspects identified, sample leprosy patients for molecular confirmation, and supervise their treatment.

Immediately following the final round of door-to-door screening of BE-PEOPLE in 2026, a PGL-I survey will be conducted covering eight villages that originally were part of arm 4 of the PEOPLE trial (n = 17,000), with the aim of comparing age specific sero prevalence rates to those at baseline of PEOPLE, in 2019. The purpose of this comparison is to assess whether anti-PGL-I surveys have potential as a tool to monitor leprosy transmission. Earlier studies in Indonesia and Brazil have shown mixed results. [27, 28]

Detailed records on costs will be maintained allowing an assessment of cost effectiveness of BE-PEP compared to SDR-PEP.

Figure 2 below shows a Gantt chart of all activities planned for the period of 2022 till 2028.

Figure 2: Gannt chart of the BE-PEOPLE trial

Activity		2022				2023				2024				2025				2026			
	Q4	Q1	Q2	Q3	Q4	Q1	Q2	Q3	Q4	Q1	Q2	Q3	Q4	Q1	Q2	Q3	Q4	Q1	Q2	Q3	Q4
1. Obtaining ethics and administrative approval for study protocol	x																				
2. Finalizing study procedures	x	x																			
3. Conduct Phase 2 study			x																		
4. Go no-go decision for phase 3 based on advice of DSMB				x																	
5. Obtain green light from authorities in Comoros for phase 3				x	x																
6. Baseline survey BE-PEOPLE (overlaps with final survey of PEOPLE in 32 villages)		x	x	x	x																
7. Randomization BE-PEOPLE arm 1 and 2					x																
8. First Leprosy screening round BE-PEOPLE, Provide SDR-PEP and BE-PEP						x	x	x	x												
9. Second Leprosy screening round BE-PEOPLE, Provide SDR-PEP and BE-PEP										x	x	x	x								
10. Third Leprosy screening round BE-PEOPLE, Provide SDR-PEP and BE-PEP														x	x	x	x				

Activity		2022				2023				2024				2025				2026			
	Q4	Q1	Q2	Q3	Q4	Q1	Q2	Q3	Q4	Q1	Q2	Q3	Q4	Q1	Q2	Q3	Q4	Q1	Q2	Q3	Q4
11. Final Leprosy screening round BE-PEOPLE																		X	X	X	X
12. Continue sampling of incident leprosy patients	X	X	X	X	X	X	X	X	X	X	X	X	X	X	X	X	X	X	X	X	X
13. Sampling incident tuberculosis patients		X	X	X	X	X	X	X	X	X	X	X	X	X	X	X	X	X	X		
14. Collect costing data						X	X	X	X					X	X	X	X	X	X		
15. Analyze costing data										X	X									X	X
16. Anti-PGL-I testing of former arm 4 villages																		X	X	X	X
17. Interim analyses										X				X							
18. Cost effectiveness analysis																					X
19. Final analysis on effectiveness of E-PEP																					X

4. PARTICIPANTS, POPULATION & SELECTION

4.1 Study settings

The study takes place in Comoros, an island nation in the Indian ocean, just north of Madagascar. We will build on the existing setup of the PEOPLE trial on the islands of Anjouan and Mohéli. In Comoros the national leprosy and tuberculosis control program (NLTCP) provides free diagnostic and treatment services for both diseases, in accordance with international standards. The NLTCP is supported by Damien Foundation, an international NGO with headquarters in Brussels, Belgium. In Comoros the leprosy burden is concentrated on two of the three islands, Anjouan and Mohéli. On the main island, Grande Comore, only sporadic leprosy cases are reported, with higher notifications of tuberculosis. Rifampicin resistant tuberculosis is exceedingly rare, and has not yet been observed since introduction of universal Xpert MTB/RIF testing in 2017. Patients with skin conditions can present at public health centers where they are examined by general health services staff who have been trained in diagnosing and treating leprosy. They are supervised by staff from the NLTCP, to whom they can also refer patients for consultation. Specific treatment for leprosy is provided free of charge, either directly or through the primary healthcare system. In addition outreach campaigns at village level are organized in which persons with skin conditions are invited to present themselves for screening. Free treatment is provided for minor skin conditions and any leprosy patient diagnosed receives full treatment. Such outreach campaigns, also called ‘mini-campaigns’ have been introduced in Comoros since 2008. For the BE-PEOPLE trial, door-to-door screening activities will be carried out by teams that were established in the framework of the PEOPLE study, under the direct supervision of the NLTCP and under responsibility of the country PI. The teams will receive the necessary protocol and (refresher) GCP training prior to the start of the BE-PEOPLE study. They will also receive training on aspects of concomitant medication. Any leprosy patients identified will be treated according to the national guidelines of Comoros, which are in accordance with WHO guidelines.

4.2 Study population and selection criteria

For the phase 2 study we have selected the village of Gégé, a leprosy endemic village on the island of Anjouan at one hours’ driving distance of the main hospital in the capital. The village has an estimated population of approximately 900 and is currently part of arm 1 op PEOPLE, the comparator arm. Therefore no PEP has been provided yet although 8 leprosy cases were diagnosed during the PEOPLE trial so far. The village will be screened once more in the framework of the PEOPLE trial, in May, 2022. After this final screening, BE-PEP and SDR-PEP will be provided for the phase 2 study of E-PEOPLE. The village will not be part of the phase 3 study of BE-PEOPLE. A random sample of 150 eligible participants will receive BE-PEP, the remainder will receive SDR-PEP. Those receiving BE-PEP as well as a random sample of 150 among those receiving only SDR-PEP will be strictly monitored for adverse events. As an additional safety measure BE-PEP administration will start with adults 18 years and older, followed by children aged 13-17, and finally children aged 5-12. Provision of BE-PEP to children aged 13-17 will start once post BE-PEP QTc measurements for adults are available and have shown no indication for concern, similarly distribution to children aged 5-12 will start once post BE-PEP QTc measurements for children aged 13-17 are available and show no reasons for concern.

Inclusion and exclusion criteria for the phase 2 study will be the following:

<u>Inclusion criteria:</u>

1. Being a permanent resident of the study village, in good state of health
2. Able and willing to provide informed consent
3. Age 5 years or above and weight of 20 kg or above.

Exclusion criteria

1. Signs of active leprosy
2. Signs of active pulmonary tuberculosis (cough ≥2 weeks duration)
3. Signs of active extra-pulmonary tuberculosis (bluish-red nodules that cover the lymph nodes, bones or joints, or cervical glands with discharge)
4. History of liver- or kidney disease
5. Allergy to rifampicin or bedaquiline
6. Having received rifampicin or bedaquiline (if applicable) in the last 2-year period
7. Not able to swallow bedaquiline 100 mg tablets
8. Self-reported (suspected) pregnancy or breastfeeding
9. Concurrent (within the last three week period before D0) use of medications not included in the safe list (for bedaquiline only)
10. QT-prolongation of ≥450 msec in baseline ECG within the last week.
11. Jaundice, or self-reported liver function abnormalities or hepatitis
12. Value of baseline ALT or AST >3x ULN within the last week. In case only ALT is available, this would suffice for enrollment.

Children between 2 and 5 years of age or weighing between 10 and 20 kg will be offered SDR-PEP but not BE-PEP.

For the phase 3 study we will include 34 villages already part of the PEOPLE trial plus 9 additional villages/towns, they will be regrouped into 44 clusters be randomized to arms 1 or 2. New villages/clusters selected include the town of Fomboni on Mohéli and the villages of Adda Daoueni, Domoni, Koni Djodjo, Koni Ngani, Dzindri, Ouani, Mirontsi and Tsembéhou on Anjouan. Fomboni will be split into two clusters, of the other villages we will retain either the entire village or the part with the highest leprosy prevalence. As will be explained in more detail in the section on sample size, we aim to include a total study population of approximately 124,000 for the new trial, approximately 30% of the total population on Anjouan and Moheli.

Inclusion and exclusion criteria for the phase 3 study will be the following:

Inclusion criteria:

1. Living in one of the study clusters (34 on Anjouan, 10 on Mohéli), in good state of health
2. Aged 2 years and above, as leprosy is very rare among infants and young toddlers. Children age 2-4 years or weighing less than 20 kg will not be given bedaquiline. If eligible they will receive only rifampicin.
3. Able and willing to provide informed consent for leprosy and tuberculosis screening, and PEP administration (as applicable in the different arms)

Exclusion criteria

1. Signs of active leprosy
2. Signs of active pulmonary tuberculosis (cough ≥2 weeks duration)
3. Signs of active extra-pulmonary tuberculosis (bluish-red nodules that cover the lymph nodes, bones or joints, or cervical glands with discharge)
4. Having received rifampicin or bedaquiline (if applicable) in the last 2-year period
5. Self-reported (suspected) pregnancy or breastfeeding
6. Concurrent (within the last three week period before D0) use of medications not included in the safe list (for bedaquiline only)

All leprosy patients diagnosed during the study period, even those from non-study villages, will also be enrolled in a sub-study in which skin biopsies will be collected from non-facial lesions. qPCR on these samples will allow to verify the accuracy of the clinical diagnosis and further processing with Deeplex Myc-Lep V3 of qPCR positive samples will allow to monitor drug resistance. As drug resistance may also affect treatment for tuberculosis, we will also sample all GeneXpert positive tuberculosis patients identified on Anjouan, Mohéli, and Grande Comore during the study period for drug resistance testing with Deeplex Myc-TB XL, and verify whether they received rifampicin and/or bedaquiline in the past.

Leprosy patients will be enrolled from all villages of Anjouan and Mohéli, including those not in the BE-PEOPLE trial and including patients diagnosed in BE-PEOPLE villages outside the surveys of the trial. These patients will be sampled and subjected to the Deeplex Myc-Lep V3 (or WGS if needed), in order to survey drug resistance and to assess the impact of the intervention on transmission of *M. leprae*. TB patients will be enrolled from all three islands of Comoros, from any village. We will only include those that are bacteriologically confirmed by GeneXpert, and subject them to Deeplex Myc-TB XL.

4.3 Sample size

The sample size for the phase 2 trial is based on non-inferiority of the BE-PEP regimen compared to the SDR-PEP regimen in terms of adverse events, in particular QTc prolongation. We assume that in the SDR group the mean QTc will be equal to that of a reference population, i.e. 384 ms (SD 20 ms) based on the Fridericia correction method. [29]

The sample size calculations were performed by simulation, in a scenario where there is truly no difference between the standard and experimental treatment. A sample size of 64 patients are required per arm to achieve 80% power in the comparison that the upper limit of a one-sided 95% confidence interval will be below the non-inferiority limit of +10 ms.

For this part of the trial we have selected a village with a total recorded population of 908, 51% of whom are aged 18 years or above. As explained in chapter 4.2 above, as a matter of precaution we will first enroll adults and preliminarily assess their outcomes before we will start enrolling minors. The numbers of adults to be enrolled will be proportional to the observed age distribution at the time of the 2022 survey. To arrive at an accurate estimate of mean QTc for adults as well as for minors, we will recruit 150 participants per study arm, approximately 75 adults and 75 minors.

Overall, all ages combined, our intended sample size of 150 in each arm, which will provide 99% power to show that the upper limit of a one-sided 95% confidence interval will be below the non-inferiority limit of +10ms, assuming that in the larger population QTc does not differ between those receiving SDR-PEP and those receiving BE-PEP. Sample size calculations were done in R version 4.0.2.[30]

To calculate the sample size required for the phase 3 trial, with the main objective of assessing the impact of BE-PEP versus SDR-PEP on leprosy risk at individual level, we used the methodology described by Hayes and Bennet for cluster randomized trials.(10) Our aim is to show a 50% reduction in risk of leprosy over a 3-year period for those that received BE-PEP (arm 1) compared to those that received SDR-PEP (arm 2). We assume that in the latter arm the risk over a 3-year period will be 0.0033, i.e. 1.1 per 1,000 per year. The mean population size of the villages targeted is 2,819, however children below 5 years of age or weighing below 20 kg are not eligible for BE-PEP and make up 27% of the population. Even though part of them will be eligible for SDR-PEP, we will exclude them from this analysis because the risk of leprosy varies with age. We will therefore only compare individuals aged 5 years or above and weighing 20 kg or above. We will also take into account the participation rate, i.e. the proportion of eligible participants that agreed to take PEP, which in the PEOPLE trial was 83%. Thus on a population of 2,850, only 1,708 will effectively be available for analysis. For the PEOPLE trial we calculated a coefficient of variation between clusters (κm) of 0.34, in the BE-PEOPLE trial we assume a

slightly higher Km, 0.4. To achieve a power (1-β) of 90% with an average cluster size of 1,708 and $\alpha = 0.05$, 22 clusters per study arm would be required, i.e. 75,152 subjects for the two arms combined. To enroll 75,152 eligible subjects we need to target a population of approximately 124,000 in total. We therefore selected 43 villages with a total population of 124,035, of which one will be divided into two clusters.

Of the 43 villages selected, 34 are already enrolled in the PEOPLE trial with a total population of approximately 73,000. In addition we have selected 9 new villages with an estimated available population of 51,000. For the new villages we have assumed a coverage of 50% only as they are large villages and we will exclude the neighborhoods from which leprosy patients are seldom reported.

For the secondary objective of assessing the effect of BE-PEP at village level, the entire population examined will be considered in the analysis. If incidence at village level is 1.1 per 1,000 per year, as observed in the PEOPLE trial, and a 50% reduction is achieved with BE-PEP, the available sample size would provide a power of approximately 90% over a three-year follow-up period.

For the surveillance for resistance in tuberculosis patients, with the Deeplex Myc-TB XL, we will include all microbiologically confirmed TB patients on the three islands of Comoros. Annually 50-60 such patients are being reported in recent years. Elsewhere, minority bacterial populations with mutations in *M. tuberculosis* gene *Rv0678*, an efflux pump regulator, which can give low level bedaquiline resistance, have sporadically been found in patients and populations never exposed to bedaquiline or clofazimine. We therefore will commence this surveillance in 2022, preceding bedaquiline introduction in the country, and throughout BE-PEOPLE also include as controls TB patients identified in Grande Comore, where bedaquiline is not used.

4.4 Randomization

For the phase 2 safety study we will first complete the final survey round of the PEOPLE study and then enumerate all those aged 5 years or above, weighing 20 kg or above and not currently suffering from leprosy. They will be divided into three age categories, 5-12 years, 13-17 years, and 18 years and above. We will then determine the overall proportions of the three age groups, the proportion multiplied by 150 will be the number of participants to be recruited per study arm for each age group. Next we will prepare sealed envelopes containing a note indicating either BE-PEP or SDR-PEP as PEP regimen, 300 in total. They will be divided into three groups with equal numbers per PEP regimen, one group of envelopes for each of the three age categories.

The next step will be to enroll the required numbers of adults (age 18 years and above) by visiting the households one by one and inviting eligible subjects to be enrolled. Each time a person agrees, an envelope will be drawn and either SDR-PEP or BE-PEP will be provided as indicated on the note inside the envelope. This process will continue until all envelopes for adults have been allocated. Provided no concerning adverse events are observed, the procedure will be repeated for the 13-17 years old. Again if no concerning adverse events are observed, the 5-12 years old will be enrolled following the same procedure. The remainder of the village population aged 2 years and above and not meeting other exclusion criteria will then be offered SDR-PEP.

Randomization for study arms 1 and 2 of the phase 3 study, will take place in December, 2022, upon completion of the baseline survey. We will then arrange all villages by island and by group (former arm 1, 2, 3 or 4 or new villages), in order of decreasing prevalence. As was explained earlier, Fomboni, the capital of Mohéli, will be split into two clusters. Thus we will arrive at 22 pairs of clusters. Blocked randomization will be used to allocate clusters into the two study arms. The randomization schedules will be prepared by an independent sponsor biostatistician. The randomization list will be prepared using SAS 9.4 (SAS Institute, Cary NC).

4.5 Withdrawal and termination of the study

4.5.1 Reasons for Withdrawal from the study

Participants may be withdrawn from the study if they withdraw consent.

4.5.2 Reasons for Withdrawal from the study intervention

Participants may be withdrawn from the study intervention if, in the opinion of the investigator, it is medically necessary, or if it is the wish of the subject. Examples of this are

- When a patient is found to be pregnant
- Treatment limiting adverse events (e.g. kidney or liver failure, or severe vomiting)
- Need for medication that interferes with study medication
- Changes that make patients no longer eligible for the study intervention according to inclusion criteria

Patients that are withdrawn from the study intervention will still be followed up as planned, since they will be analyzed in the ITT population.

4.5.3 Handling of Withdrawals

For participants that withdraw consent, no further evaluations will be performed, and no efforts to collect information will be carried out. These participants will be referred to routine care for further treatment if required.

For participants withdrawn from the study intervention, thorough efforts will be made to document study outcomes and safety data (three attempts to contact the participant in his/her home unless it is clear that the participant will not return or refuses further contacts). Participants will be scheduled for their regular follow-up visits, will undergo all study procedures and will be analyzed in the ITT analysis until the end of the study. Data collected up to the time of withdrawal will remain in the study data base and be used for analysis whether or not the participant continues with the follow-up visits.

Participants withdrawn from the study intervention for medical reasons will be referred to adequate health facilities for further care if necessary. The reason for withdrawal will be documented on the CRF.

4.5.4 Lost to follow-up

Patients will be considered lost to follow-up if not present during the final survey round in 2026, after three additional attempts have been made to contact them. In this case follow-up time will be calculated based on the last available follow-up.

4.5.5 Termination of Study

The end of the study is defined as the date of the last visit (or last contact) of the last patient in the study.

If the study is prematurely terminated or suspended, the Sponsor will promptly inform the investigators/institutions, and the regulatory authority(ies) of the termination or suspension and the reason(s) for the termination or suspension. The ECs will also be informed promptly and provided the reason(s) for the termination or suspension by the Sponsor or by the investigator/institution, as specified by the applicable regulatory requirement(s).

5. Study Procedures

5.1 General Study Procedures

The study has been planned in two phases. The main, phase 3 study will be preceded by a phase 2 study in the village of Gégé on Anjouan. In this village we will conduct door-to-door screening for leprosy in May, 2022, coinciding with the final screening round of the PEOPLE study. We will then ask for informed consent for inclusion in the phase 2 study of BE-PEOPLE. To those who consent we will provide post exposure prophylaxis with BE-PEP or SDR-PEP with intensified safety monitoring, as was described in chapter 4.4 above. Safety monitoring will include an ECG taken less than 1 week before treatment and approximately 24 hours after treatment, as well as liver function tests less than 1 week before and 14 days after treatment. In case of an ALT/AST elevation adverse event on visit D14 or absolute AST/ALT value increase of >=35 between the prerecruitment visit and visit D14, liver function tests will also be included on visit D30. The team, including a medical doctor will be present during the PEP administration round (=D0), as well as the day after (=D1) to collect safety data (including QTc). The team, again including a medical doctor, will investigate the participant at the study center 14 days after (=D14) to collect additional safety data (ALT + AST) and to follow up on any ongoing (S)AE's. In case any SAE's are ongoing during the visit on D14 additional visits will be organized to follow up. A final visit to collect adverse events will be organized on D30 at the participant's house. In case liver function tests are necessary on visit D30, the participant will be invited to the study house for visit D30.

In case of any serious adverse events transport to the hospital in the island capital Mutsamudu will be ensured, where cardiological care is provided including monitoring, electrolyte correction, and cardiac resuscitation. A project vehicle will be on standby in the village for this purpose. The results of this phase 2 safety study will be presented to the data safety monitoring board (DSMB), involved ethical committees, and local regulatory authority who will provide an opinion on whether or not the green light can be given for the main phase 3 study.

Ahead of the main phase 3 study and partially overlapping with the phase 2 study we will conduct the baseline screening which will cover 34 villages already part of the PEOPLE study, plus nine additional villages. Conditional on a positive advice by the DSMB and approval by the local regulatory authorities, randomization will take place in December, 2022, for the main study to start in January, 2023.

During the main study, each village will be screened door-to-door annually, from 2023 till 2026. Immediately following screening in each village PEP will be provided to those eligible. Prior to the start of the study the village elders will be informed on procedures and purpose of the study, followed by community sensibilization. During the screening for leprosy, residents of the selected villages will be checked for eligibility, informed on all study procedures, and requested to provide informed consent. Those previously enrolled in the PEOPLE trial will be asked informed consent for enrolment in the new trial in 2023, once we know to which study arm they have been randomized. Participants in the new villages will be asked informed consent twice, first for the baseline screening in 2022 and again in 2023, when we know to which study arm they have been randomized.

After informed consent has been obtained, the health worker will perform a physical examination to look for signs of leprosy. Diagnosis of leprosy will be made based on the so-called cardinal signs, i.e. a patch with loss of sensation, enlarged peripheral nerves and/or a slit skin smear positive for acid fast bacilli. Results of qPCR testing will not be used as diagnostic criteria, as the test is at best 85% sensitive, less so in paucibacillary patients. For patients with doubtful clinical signs, the diagnostic team does not classify them as having leprosy, yet revisits them in subsequent months for repeat diagnostic investigation, per routine care. The health worker will also collect the necessary additional personal information from every individual enrolled.

In case the household is eligible for receiving PEP (i.e. an incident leprosy case is found in the household or in the surroundings), the correct dose of bedaquiline and/or rifampicin will be distributed according to the schedule below:

- <10 kg or age <2 years : No PEP
- 10-20 kg and age 2-4 years : rifampicin:150 mg
- >=20-30 kg and age ≥5 years : bedaquiline*: 400 mg, rifampicin: 300 mg
- >30-45 kg : bedaquiline*: 800 mg, rifampicin: 450 mg
- >45 kg : bedaquiline*: 800 mg, rifampicin: 600 mg

*Arm 1 only

The health worker will offer a snack and a drink to assist with the ingestion of the pills (=D0) and will witness the intake of the medication by the participant and enter the result in a designated app. All participants enrolled on PEP, will be re-visited by a health worker the next day (=D0+1d) to ask whether the PEP has not been vomited up and whether there have been any (serious) adverse events ([S]AE). In case of ongoing SAE's at D1, the team will conduct follow-up. In case PEP has been vomited, this will be recorded but the dose will not be repeated.

The nature of the trial, requiring intake of different PEP regimens in different arms, makes blinding impossible. We will however use additional approaches to validate study results and detect possible observer bias, such as quantitative PCR (Qpcr) testing of skin biopsy samples from leprosy patients diagnosed in both study arms.
If a screened person is diagnosed with leprosy, he/she will be requested to sign an additional consent form for the collection of samples. Irrespective of further participation in the study, this person will be treated for leprosy as per the standards of the National Leprosy Control Program.

Tables 2 and 3 below provide an overview of assessments during phase 2 and 3 respectively.

TABLE 2: SCHEDULE OF ASSESSMENTS PHASE 2

		Screening	***Enrollment***	***Day 1 (V1)***	***Day 14 (V2)***	***Day 30 (V3)***
Timing (window)			D0	D1 (+0 d.)	D14 (+-2 d.)	D30 (+3 d.)
Medical history[1]		X				
Inclusion/exclusion criteria		X				
Randomization			X			
Informed Consent		X				
Clinical examination		X	X			
Blood sample for ALT/AST		X			X	(X)[4]
ECG		X		X		
IP Administration[3]			X			
Concomitant medication[4]		X	X	X	X	
Adverse Event collection			X	X	X	X

1 Medical history deemed medically relevant for the study will be collected before signing informed consent to participate in the phase 2 trial.

2 All PEP treatment will be delivered by the health worker and intake will be directly observed.

3 Concomitant medication should be recorded from 3 weeks before D0 (day of IP administration) until 3 weeks after IP administration. After that concomitant medication should only be reported if given for an SAE.

4 In case of an ALT/AST elevation adverse events on visit day 14 or absolute AST/ALT value increase of >=35 between the prerecruitment visit and visit day 14, liver function tests will also be included on visit day 30. In this case the participant will be invited to the study house to conduct visit D30.

TABLE 3: SCHEDULE OF ASSESSMENTS PHASE 3

	Leprosy Screening (V0)	*Screening*	*Enrollment (V1)*[7]	*Safety FU V1*	*Year 2 (V2)*	*Safety FU V2*	*Year 3 (V3)*	*Safety FU V3*	*Year 4 (V4)*
Timing (window)		*V1 D0*	*V1 D0*	*V1D0 +1 (+- 0d)*	*V1 D0 + 365 (+-60)*	*V2D0 +1 (+-0d)*	*V2 D0 + 365 (+-60)*	*V3D0 +1 (+-0d)*	*V3 D0 + 365 (+-60)*
Medical history[1]	X	X			X		X		X
Inclusion/exclusion criteria		X							
Informed Consent	X[2]	X							
Clinical examination	X	X		X	X	X	X	X	X
IP Administration[3,4,5]			X		X		X		
Concomitant medication[6]	X		X	X	X	X	X	X	
Adverse Event collection			X	X	X	X	X	X	

1 Medical history deemed medically relevant for the study will be collected before signing informed consent to participate in the phase 3 trial.
2 For new villages (not included in the PEOPLE study) a baseline screening ICF is collected during this visit. For villages enrolled in the PEOPLE study, informed consent was collected in this study to collect data and to share data with future research such as the BE-PEOPLE study.
3 Conditional on eligibility and exposure to index cases
4 All PEP treatment will be delivered by the health worker and intake will be directly observed.
5 In arm 1, household contacts of an index case will receive an extra dose of BE-PEP after 4 weeks (date of IP dosing (V1D0 / V2DO/ V3D0) + 28 days (+- 2 days)
6 Concomitant medication should be recorded from 3 weeks before D0 (day of IP administration) until 30 days after D0, after that concomitant medication should only be reported if given for an SAE.
7 The screening and enrollment will be performed on the same day.

5.2 Laboratory- and safety monitoring procedures

5.2.1 Phase 2 pharmacovigilance: monitoring for hepatitis

In the Phase 2 study, participants will have venous blood drawn within 1 week prior to PEP administration for baseline ALT/AST (GPT) determination on serum in a dedicated and calibrated biochemistry analyzer at the central laboratory in the hospital Hombo or Bambao. Those who at baseline have an ALT or AST value > 3 times the upper limit of normal (ULN) will not be offered PEP. The venous blood draw for ALT/AST determination will be repeated 14 days after PEP administration. In case of an ALT/AST elevation adverse events on visit D14 or absolute AST/ALT value increase of >=35 between the prerecruitment visit and visit D14, liver function tests will also be included on visit D30. Any surplus remaining serum may be used for exploratory analyses of leprosy related biomarkers at the LUMC.

5.2.2 Sampling schedule for clinically confirmed leprosy patients

The local leprosy control teams on each island will be responsible for identifying new leprosy cases and collecting the samples. All samples will be identified by a patient-specific barcode only. Both a nasal swab and skin biopsy sample from the lesion(s) (except from lesions in the face) are being collected

from all newly diagnosed patients. In addition, for those patients who have MB leprosy, skin slit smears (SSS) are taken. Fingerstick blood samples will be collected from all newly diagnosed patients.

Slit skin smears (SSS) will be collected from all consenting MB participants. The SSS will be taken from the two earlobes and one additional site presenting an active lesion. For this, skin will be pressurized and a small incision will be made in the skin and scraped, followed by spreading the smear over the surface of the glass slide. After drying the slides, they will be fixated by heating on site. The SSSs will be colored by the ZN staining in the local laboratories by the designated local leprosy control teams at the end of each day of sampling. The subsequent microscopy reading of these slides will be done in the local laboratories. Quality control of microscopy reagents is conducted at the central laboratory in Grande Comore. Rereading of slides is performed as quality assurance.

Skin biopsy (SB) samples will be obtained from all consenting leprosy cases by using a 4 mm punch and the removed tissue placed in a sterile 1.5 mL tube and stored in disolol for DNA extraction (not for histopathology). Local anesthesia with 2% lidocaine will be administered subcutaneously prior to punching the biopsy.

Nasal swabs (NS) will be obtained from all consenting leprosy cases by gently rubbing a perinasal swab, in one side of each nostril over the lateral conchae. After collection, the swab will be immersed (and broken off at the breakpoint) in a sterile and labelled tube containing disolol.

Tongue swabs (TS) will be obtained from all consenting leprosy cases by gently rubbing 2/3rd of the tongue for 15 seconds. After collection, the swab will be immersed (and broken off at the breakpoint) in a sterile and labelled tube containing disolol.

Blood samples (BS) blood drops are obtained from all consenting leprosy cases by finger prick. The blood is captured in a capillary and subsequently put in a tube containing buffer.

5.2.3 Sampling of presumptive TB patients

Tongue swabs (TS) will be obtained from all consenting presumptive cases by gently rubbing 2/3rd of the tongue for 15 seconds. After collection, the swabs will be put (and broken off at the breakpoint) in a sterile and labelled tube.

Sputum will be collected from the presumptive TB patients to confirm TB, which is part of routine diagnosis.

5.2.4 Specific tests

The molecular tests (RLEP qPCR, RLEP LAMP assay, Deeplex Myc-Lep, WGS,...) on all the samples (except for the fingerstick blood samples) will be executed by ITM. In 2022, ITM will help implement DNA extraction and the RLEP LAMP assay in the main laboratories on Anjouan and Mohéli, towards enhancing local capacity for molecular confirmation of leprosy. After validation against RLEP qPCR results, the confirmation of leprosy patients in the trial may be performed in Comoros. These molecular tests are very important to assess the impact of the intervention on transmission of *M. leprae* and to closely survey the possible emergence of drug resistance.

During the final survey round in former arm 4 villages in 2026, analysis of the Anti-PGL I LFA (fingerstick blood sample) will be done by the local leprosy control team with a portable reader, with quality assurance performed by the University of Leiden.

Slit skin smears

The SSSs will be colored by the ZN staining in the local laboratories by the designated local leprosy control teams at the end of each day of sampling. The subsequent microscopy reading of these slides will be done in the local laboratories.

Fingerstick blood

The fingerstick blood collected in a buffer, will be brought to the local laboratory at the end of each day of sampling and run on the LFA strips (provided by the University of Leiden). The strips must dry up and are read by the portable reader (provided by the University of Leiden).

Skin biopsies and SSS

a. *DNA extraction*:

DNA will be extracted with the Maxwell LEV extraction procedure from biopsies, swabs and slit skin smears.

b. *Diagnostic RLEP qPCR/LAMP*:

The RLEP qPCR is a highly sensitive and specific diagnostic qPCR, which targets the RLEP repetitive element, which is specific for *M. leprae*. This test targets 35 out of the 37 RLEP repetitive elements occurring in the genome of *M. leprae*. After DNA extraction, all the samples will be processed by this RLEP qPCR. If the RLEP LAMP assay is validated against RLEP qPCR, it can be used for confirmation.

c. *Genotyping:*

Genotyping will be performed on DNA extracts that are *M. leprae* positive as defined by a positive RLEP diagnostic test. We expect that samples from all MB patients and a subset of PB patients will have sufficient DNA for further genotyping. Genotyping strains circulating in the country is essential for measuring the impact of the intervention of transmission and to survey the possible emergence of drug resistance. Whole genome sequencing may be done to obtain higher resolution on identification of transmission chains and to assess for mutations (outside the targeted regions of Deeplex Myc-Lep) that could possibly be linked with drug resistance.

Genotyping will be done by a novel sequencing technique called the Deeplex MycLep assay, which consists of a single hi-plex amplification of 18 phylogenetic SNPs, 11 VNTR markers, and drug resistance conferring gene regions followed by next generation sequencing analysis. This assay is being validated in the PEOPLE study. We will ask Genoscreen to expand the multiplex PCR with the *M. leprae atpE* target to be able to perform surveillance for bedaquiline resistance, resulting in a next version of the Deeplex MycLep assay . *M. leprae* does not possess the efflux pump involved in *M. tuberculosis* cross resistance to bedaquiline and CFZ. ITM will perform amplification and targeted deep sequencing of the resulting amplicons, with quality control done at Genoscreen.

Presumptive tuberculosis patients will be managed according to the national guidelines, not part of this study protocol. Sputum samples and possibly tongue swabs will be collected from patients with presumptive TB, and will be stored in ethanol after smear microscopy (sputum) and Xpert MTB/RIF (sputum and possibly tongue swabs). DNA will be extracted for Deeplex MycTB XL at ITM from those with sufficient bacillary burden. WGS may be done to assess additional drug resistance targets of TB strains

5.2.5 Shipment and storage of samples

The samples from Comoros will be shipped to the ITM for further analysis. Fingerstick blood samples collected during the PGL-I survey in 2026 will be shipped to LUMC. MTAs will be written accordingly and signed by all parties concerned.

Samples will be stored for 20 years, as indicated in section 12.3 below this will be mentioned in the informed consent form.

6. Study Investigational Product

6.1 Purchasing, preparation and administration

Bedaquiline in the form of SIRTURO 100mg tablets will be donated by Janssen Pharmaceutica NV and shipped to Comoros by Damien Foundation. During the phase 3 trial 20 mg dispersible tablets will also be made available.

Rifampicin in the form of 150 and 300 mg capsules has been selected from McLeods, a WHO Prequalified manufacturer. The product will be shipped by the supplier directly to the national leprosy control program of the Comoros.

No medication for treatment of skin diseases will be purchased for study purposes. All treatment is part of the routine leprosy control programs in the Comoros.

6.2 Participant compliance monitoring

All PEP treatment will be delivered by the health worker and intake will be directly observed. The health worker will indicate in the specific form of the electronic study database in REDCap that the participant has taken the medication, including the dose.

The site staff will keep a study-specific inventory of all medication that is received for the study and all medication that has been distributed. IP Accountability at participant level will be documented by entering the IP dose in redcap.

6.3 IMP preparation and administration

The administration of tablets of bedaquiline and of rifampicin capsules will be described in an IMP preparation and administration SOP.

6.4 Prior and concomitant therapy

Rifampicin is known to interact with other drugs, anti-retrovirals (ARV) in particular.[31] It is an inducer of various genes controlling drug metabolism and transport, such as cytochrome P450 isoenzymes and the drug efflux pump p-glycoprotein. As a result plasma concentrations of concomitantly administered drugs may be reduced. In this study only one single dose of Rifampicin will be provided. As Rifampicin has a serum half-life of less than 5 hours, the effect of such interactions would be expected to be negligible, regardless of the dosing. [32]

Even though due to the single dose administration of the drugs, no drug-drug interactions or QT prolongation are expected, the concurrent use of additional QT prolonging drugs with single dose bedaquiline is not recommended.[33] Study participants will be asked whether they take any other medications. These will be checked against a list of permitted medications to be used together with bedaquiline. If drugs are not on the permitted list, and not on the list of prohibited drugs, the ITM team will review the specific drug for interactions with bedaquiline and add to the permitted or prohibited list. The participant whose medication was permissible may subsequently still receive BE-PEP.

6.5 Packaging

The commercial formulation of rifampicin (capsules of 150 mg and 300 mg) procured for the study will be used. Bedaquiline tablets of 100mg are provided in blister packs protected against humidity and light.

6.6 Reception, storage, dispensing and return

All study drugs will be stored in the central air-conditioned and temperature-controlled stock of the national leprosy control program, this for all islands. For Bedaquilline the temperature cannot surpass 40 °C, for rifampicine the temperature cannot surpass 40 °C. If the temperature surpasses 40 °C for rifampicin or bedaquilline, the sponsor should be contacted immediately. The medication cannot be used before the sponsor's approval. If medication is shipped between islands in the Comoros, a temperature monitor device should always accompany the shipment. The temperature monitoring log should be read out right before medication is inventorised.

The mobile teams will be supplied with sufficient medication during their screening visits to the different villages. This medication will be kept at ambient temperatures. Medication that has been provided with a mobile team and that is returned to the central stock, will no longer be used as the conditions in which it was stored cannot be guaranteed. An inventory will be kept of all incoming and outgoing medication. The inventory should also be documented for all medication that was returned after field visits, as this medication should be destroyed. Expired study drugs and study drugs that returns from the field will be separated from usable inventory and quarantined to prevent accidental assignment and/or dosing to subjects.

The final fate of unused bedaquiline will be agreed at the end of the study. In case of destruction, this will be documented on a dedicated destruction log.

Left-over, non-expired, Rifampicin medication will be used in routine care. Unused expired rifampicin can be destroyed during the conduction of the study, but only after the monitor was on site to give his/her approval.

7. Safety Assessment

7.1 Rationale for safety and efficacy of study drugs

7.1.1 Safety of Rifampicin 600mg

The regimen used for prophylaxis in arm 2, single dose Rifampicin, has been extensively tested in a number of leprosy PEP trials and is the current choice when PEP is implemented programmatically. In 2018 WHO issued a guideline for the diagnosis, treatment and prevention of leprosy which states that: 'Single-dose rifampicin (SDR) may be used as leprosy preventive treatment for contacts of leprosy patients (adults and children aged 2 years and above), after excluding leprosy and tuberculosis (TB) disease, and in the absence of other contraindications'.[34]

Indeed, in the first two years of the PEOPLE trial in which rifampicin was used at a dose of 1200 mg, no adverse events were identified related to the study medication. In BE-PEOPLE the WHO recommended dose of 600mg will be used as comparator arm, in order to test whether bedaquiline enhanced WHO recommended PEP is more effective at preventing leprosy.

7.1.2 Expected efficacy of bedaquiline 800mg against *M. leprae*

In *M. leprae* infected mice, a single 25 mg/kg dose of bedaquiline killed more than 95% of viable *M. leprae* bacilli and maximum effect (Emax) was reached after such a single bedaquiline dose of 25 mg/kg. The equivalent efficacy of daily and monthly bedaquiline dosing in the mouse footpad model indicates that bacterial killing is driven by total area under the time-concentration curve (AUC) and not by Cmax. The minimal effective dose for TB is 6.5 mg/kg when given 5 times per week and the lowest fully bactericidal dose is 12.5 mg/kg. The minimum effective dose to kill *M. leprae* (1 mg/kg) is thus 6 times lower than the minimum effective dose to kill *M. tuberculosis* in mice. In mice, a 25-mg dose is associated with an AUC of 20 µg.h/mL. In order to take into account an expected equal contribution of the M2 metabolite, the target AUC of 40 µg.h/mL bedaquiline was selected (i.e., similar exposure as obtained in the mouse model at 25 mg/kg once monthly). In a thorough QT/QTc study in healthy human volunteers, a dose of 800 mg, administered under well controlled feeding conditions, resulted in an AUC24h of 71.7 µg.h/mL (N=44, primarily non-black). However, in a programmatic setting, exposure may be lower: in the phase 2 studies in MDR-TB patients (C208 and C209), it was concluded that bioavailability in these patients was 50% lower. Additionally, a 34% lower exposure is expected in black patients. Based on these data, 800 mg bedaquiline is needed to obtain the target AUC of 40 µg.h/mL.

Janssen has completed recruitment in a proof-of-concept study of bedaquiline treatment of active leprosy in 11 patients, 4 of whom have completed 8 weeks of bedaquiline monotherapy. The preliminary safety data indicates that 8 weeks of bedaquiline monotherapy was safe and well tolerated in MB leprosy patients at the dose studied (200 mg once daily for 2 weeks followed by 100mg three times a week for the following 6 weeks). The outcome results are pending.

In September 2021, Janssen submitted a request for scientific advice to EMA to seek advice on the trial design for a phase 3 trial for bedaquiline based as part of a multi-drug therapy for the treatment of multibacillary leprosy. The proposed dosing of bedaquiline in a 6 month regimen (including clofazimine and rifampin) is 200mg qd for 2 weeks followed by 800mg q4w for the remaining 5 months.

7.1.3 Safety of Bedaquiline 800mg

In tuberculosis treatment bedaquiline is used typically at 400 mg once daily for two weeks followed by 200 mg three times a week for 24 weeks. Bedaquiline can cause QT prolongation, leading to cardiac arrhythmia and/or death. In data submitted to the FDA, a higher mortality was observed in TB patients who received bedaquiline than among those treated with other drugs (11.4 versus 2.5%). In recent larger clinical studies, no safety concerns arose with use of bedaquiline, and RR-TB patients treated with bedaquiline experienced a mortality benefit.[35, 36] The availability of safety data in healthy volunteers at single dose of 800mg, combined with the efficacy data of an equivalent dose in mice against *M. leprae* described above, supports the choice for this dose of bedaquiline.

Janssen is conducting a paediatric trial of bedaquiline for rifampicin resistant tuberculosis. The adolescent- and age 5-12 year arms have completed recruitment but the younger age groups are still ongoing. The recommended doses in children 5-<12 yrs of age and weighing >=15 kg are weight dependent. Based on a collective analysis of the 2 completed study arms and applying Population pharmacokinetics based predictions to the larger population, the recommended dose in children 5-12 years old and adolescents, weighing >= 30 kg is the same as in MDR-TB adults, reflecting similar clearance as in adults, whilst the recommended dose in children 5-12 years old and adolescents, weighing >= 20 to < 30 kg is half the adult MDR-TB regimen, reflecting approximately 50% reduction in clearance. These recommended dosages lead to similar exposures in the respective pediatric age and weight ranges as observed in adults following the adult dosing regimen. Based on the non-compartmental analysis in the pediatric trial, both in adolescent patients with MDR-TB (after multiple-dose administration of bedaquiline at a dosage of 400 mg/day for the first 2 weeks followed by a

dosage of 200 mg 3 times per week) and in children (5 to <12 years) with MDR-TB (after multiple-dose administration of bedaquiline at a dosage of 200 mg/day for the first 2 weeks followed by a dosage of 100 mg 3 times per week) the M2 PK was consistent with the multiple-dose PK of M2 in adults with MDR-TB.

The risk of QT prolongation by bedaquiline is mainly driven by the M2 Cmax.[37] Based on exposure-QT M&S, the maximal effect of M2 (Emax,M2) on QT prolongation is 28.6 ms, and the EC50 (effective concentration to reach Emax,M2) is 855 ng/mL. Assuming 50% M2 accumulation after two doses of 800 mg, 4 weeks apart, and bioavailability similar to that observed bioavailability in healthy volunteer phase 1 studies, the predicted (worst-case) M2 Cmax after 2 doses of 800 mg, 4 weeks apart, is lower than 130 ng/mL, i.e., lower than the QT EC50 concentration of 855 ng/mL and lower than the M2 Cmax at steady state (Cmax,ss) of bedaquiline in the recommended MDR-TB regimen, (ie, 361 ng/mL). To follow up on the possible risk of QT prolongation in the Phase 2 study, ECG monitoring is foreseen.

Bedaquiline and M2 are not substrates of P-gp and breast cancer resistance protein (BCRP) in vitro, bedaquiline is a weak substrate of organic anion transporting polypeptide (OATP) 1B1, and OATP1B3. M2 is not a substrate of OATP1B1 and OATP1B3. Bedaquiline and M2 did not inhibit hepatic uptake transporters OATP1B1 and OATP1B3 or efflux transporters P-gp, BCRP.

The effect of repeated dosing of rifampicin QD on the PK of bedaquiline was studied in healthy volunteers. Acknowledging this design is not sensitive to evaluate the OATP1B inhibitory effect by rifampicin specifically, the net effect after repeated rifampicin administration (3A4 induction and inhibitory effects), showing a similar effect on Cmax and AUC suggests no substantial effect on OATP1B-mediated transport of bedaquiline. Cmax is a sensitive PK parameter to detect effect by potential inhibitors on liver uptake.

In addition, there is indirect in vivo evidence confirming that bedaquiline PK is not sensitive to OATP1B transporter inhibitors: as per the FDA, Ritonavir, lopinavir and clarithromycin are OATP1B inhibitors.[38] In vivo drug-drug interaction studies with these perpetrators were conducted and showed no substantial effect on bedaquiline Cmax.

7.1.4 Rationale for combining bedaquiline 800mg and rifampicin 600mg

Synergy between bedaquiline (which targets the mycobacterial respiratory chain) and rifampicin (which inhibits RNA polymerase) was observed in murine studies for *M. tuberculosis*, supporting the use of this combination for leprosy post-exposure prophylaxis.

Bedaquiline has no known drug-drug interaction (DDI) liability as perpetrator. As 3A4 substrate, however, inhibition or induction of CYP3A4 may alter the PK of bedaquiline. Rifampicin is a known CYP3A4 inducer, with maximal inductive effect reached between 1 to 2 weeks. However, a single dose of rifampicin does not induce CYP3A4. The effect of repeated dosing of strong CYP3A4 inhibitors (ketoconazole, lopinavir/ritonavir) on single dose of bedaquiline was evaluated. No clinically relevant effect on Cmax was observed. An increase of the AUC with 22% was observed. In conclusion, there are no significant DDI liabilities anticipated that may require excluding certain concurrent medications when administering BDQ and Rifampin as single dose or two single doses, 4 weeks apart. There is one exception; if strong CYP3A4 inducers would have been administered repeatedly before the single dose of BDQ, that may reduce exposure to BDQ.

The bioavailability of bedaquiline increases with food (2-fold increase in AUC, see label). The exposure of rifampicin, when administered with food, decreases slightly but this is not considered clinically relevant. Therefore, the drugs will be administered under fed conditions.

7.2 Safety monitoring

7.2.1 Safety monitoring for QT prolongation in Phase 2

In March 2022, a training on QT measurement and interpretation on 12-lead ECG and the Kardia 6L will be given to the study team by a referent cardiologist with extensive experience in the use of bedaquiline for tuberculosis treatment, the cardiologist at a specialist cardiology service., and the study investigator. Both dedicated 12 lead ECG instruments with electronic and paper data capture will be used as well as the Kardia app (Alivecor), which was FDA approved for the diagnosis of atrial fibrillation, and has been validated against 12-lead ECG based QTc measurements with a good correlation. [39] The measured QT will be corrected by the Fridericia formula through a dedicated calculator.

In the phase 2 study planned for May 2022, participants will have their QTc interval screened at baseline (within 1 week before PEP administration) by the Kardia app and a 12-lead ECG (reference standard) to validate the accuracy of the Kardia app in this specific setting. If QTc ≥ 450ms is confirmed by qualified readers, the participant will be excluded from the Phase 2 safety study. If the QTc by 12 lead ECG is found to be <450ms, the participant is not excluded. All ECGs will be time stamped and stored as pdf in the RedCap database for blinded review by a referent qualified reader based at the department of Cardiology of the Amsterdam University Medical Center location AMC.

Those participants with QTc <450ms, and whose baseline ALT is <3x ULN, will receive study treatment if eligible. QTc will be measured 24 hours later, and ALT 14 days after administration.

If after single dose administration of bedaquiline and/or rifampicin, and in the unexpected event that QTc prolongation >60ms since baseline or a value of >500ms is seen, the participant will be urgently brought to the cardiology service at a specialist cardiology servicefor further examination, continuous rhythm monitoring, repeated QTc measurements, and measurement of potassium for correction of hypokalemia. The ECG will be reviewed for additional warning signs of impending malignant dysrhythmias and IV magnesium and isoprenaline may be administered, per the cardiologist's management. These procedures will be described in a separate cardiology monitoring SOP.

7.2.2 Safety monitoring for hepatitis in Phase 2

For laboratory measurements see section 5.2.1. In case of ALT or AST values of >3x ULN at baseline, participants will not be eligible for study medication. In case of ALT/AST elevations >3x ULN after study medication, participants will undergo further ALT/AST monitoring with frequency and need for referral depending on the level of elevation.

7.2.3 (S)AE monitoring

The occurrence of any adverse events (AE) will be assessed the day after administration. An AE is any untoward medical occurrence in a clinical investigation subject administered a pharmaceutical product and which does not necessarily have a causal relationship with this product. It can be any unfavorable and unintended sign, symptom, or disease temporally associated with the use of a medicinal (investigational) product, whether or not related to this product. Participants who receive PEP will be instructed to inform the study staff of any adverse events between the administration of the drug and 30 days after the last dose of administration. All relevant study staff will be trained on (S)AE detection, (S)AE monitoring mechanisms and (S)AE reporting on site. During the training, examples of (S)AE reporting cases will be given. SAE checklists and forms will be explained as well.

In the Phase 2 study, a physician from the study team will be available in the village and a cardiologist will be on call at a nearby specialist cardiology service.. In the Phase 3 study, the participants are encouraged to contact the study staff while the study teams are still in the village, or afterwards using the contact details in the information form of which each household will receive a copy. For all participants provided study medication, study teams will- on arrival in the village the next morning- start by visiting their houses to verify for any (S)AEs. They will specifically enquire about gastro-intestinal (nausea, vomiting, diarrhea), nervous system-related (headache, dizziness), cardiac symptoms (syncope) and cutaneous reactions. These reported adverse events will be recorded, together with their severity and relatedness to the study medication.

Medical staff in health facilities in the districts concerned will also be informed about the trial in advance (and at the beginning of every yearly screening round) and will be asked to report any possible adverse reactions (AR) or serious adverse event (SAE) to the study team. An AR is an AE for which there is at least a reasonable possibility that it might be linked to the medicinal product under study. A SAE is an AE that 1) results in death, 2) is life-threatening, 3) requires inpatient hospitalization or prolongation of existing hospitalization, 4) results in persistent or significant disability/incapacity, or 5) is a congenital anomaly/birth defect. ARs will be reported in the same manner as AEs. In case of a SAE however, the SAE report template will be completed by the study team and sent to the sponsor (pharmacovigilance@itg.be) within 24 hours following the notification of the SAE to the study team as per the SAE reporting instructions. Only events (AE, AR and SAE) that occur between the administration of the drug and 30 days after the last dose of administration will be recorded for study purposes. Any additional safety information obtained until 30 days after the last dose of the Janssen Product(s) Under Study will be reported to Janssen Pharmaceutica NV.

All SAE's whether or not deemed drug related or expected must be reported immediately or within 24 hours (one working day) using the SAE Notification Form by email to:

Clinical Trials Unit
Institute of Tropical Medicine
Nationalestraat 155
B-2000 Antwerp – Belgium
Email: pharmacovigilance@itg.be

Line listings of all reported SAE's will be sent to the IRB of the ITM and the independent EC, on a yearly basis.

A study safety checklist SOP will be prepared describing all responsibilities and actions that are required in case of an SAE. Only events (AE, AR and SAE) that occur between the administration of the drug and 30 days after the last dose will be recorded for study purposes. All subsequent AEs and SAEs spontaneously reported beyond 30 days after the last dose of the Janssen Product(s) under study shall be collected/reported if the investigator considers the AE/SAE to be causally related to the use of the Janssen Product(s) under study.

Concomitant medication will be recorded in the REDCap study database and as explained in section 4.2 above, a safe list will be provided that contains all essential medications registered in Comoros that are not considered a risk when administering BE-PEP. For any medication encountered that is not on this safe list and that is also not on the list of forbidden medications, the field teams will consult the local PI (Dr. Younoussa Assoumani) who will decide whether it will be added either to the safe list or to the list of forbidden medication. As part of the baseline survey in the new villages of BE-PEOPLE that

were not part of the PEOPLE trial, we will collect information on medications used to allow us to updated the safe list ahead of the start of the intervention in 2023.

For BE-PEOPLE, an independent Data Safety Monitoring Board (DSMB) will review the (S)AEs and outcome of the Phase 2 study, with pre-specified criteria on stopping (see section 8) versus continuing in the Phase 3 study. During the phase 3 study the DSMB will also review active (S)AE follow-up on the day after study medication administration.

NOTE (1): In general, hospitalization signifies that the participant has been detained (usually involving at least an overnight stay) at the hospital or emergency ward for observation and/or treatment that would not have been appropriate in the physician's office or out-patient setting. Complications that occur during hospitalization are AEs. If a complication prolongs hospitalization or fulfils any other serious criteria, the event is an SAE. When in doubt as to whether "hospitalization" occurred or was necessary, the event should be considered an SAE. Hospitalization for elective treatment of a pre-existing condition that did not worsen from baseline is not considered an SAE, nor hospitalization for non-medical reasons (e.g., the participant stays at the hospital overnight because (s)he lives too far and/or there is not transport).
NOTE (2): The term disability means a substantial disruption of a person's ability to conduct normal life functions. This definition is not intended to include experiences of relatively minor medical significance such as uncomplicated headache, nausea, vomiting, diarrhea, influenza, and accidental trauma (e.g. sprained ankle) which may interfere or prevent everyday life functions but do not constitute a substantial disruption.
NOTE (3): The term 'life-threatening' in the definition of 'serious' refers to an event in which the subject was at risk of death at the time of the event. It does not refer to an event, which hypothetically might have caused death, if it were more severe.

All SAEs must be:
- recorded on the appropriate SAE report form
- followed through resolution by a study clinician
- reviewed and evaluated by a study clinician.
- Follow-up until resolution (by sending updates on the SAE form)

Severity, relationship of event to study drug and outcome
All SAE's will be assessed by the clinician using a predefined grading system:
1. **Mild:** events require minimal or no treatment and do not interfere with the participant's daily activities.
2. **Moderate:** events result in a low level of inconvenience or concern with the therapeutic measures. Moderate events may cause some interference with functioning.
3. **Severe:** events interrupt a participant's usual daily activity and may require systemic drug therapy or other treatment. Severe events are usually incapacitating.
4. **Life-threatening:** Participant at risk for death at the time of the event

Changes in the severity of an SAE will be documented to allow an assessment of the duration of the event at each level of intensity to be performed. Adverse events characterized as intermittent require documentation of onset and duration of each episode.

Assessment of causality
The investigator is obliged to assess the relationship between investigational product and the occurrence of each SAE. The investigator will use clinical judgement to determine the relationship. Alternative causes, such as natural history of the underlying diseases, concomitant therapy, other risk

factors, and the temporal relationship of the event to the IMP will be considered and investigated. The investigator will also consult the drug information and the DSMB as needed in the determination of his/her assessment.

The relationship of an adverse event to study drug is to be assessed according to the following definitions and can only be done by the study physician:

1. **Definitely unrelated**: Reserved for those events which cannot be even remotely related to study participation (e.g. injury caused by a third party).
2. **Unlikely**: There is no reasonable temporal association between the study drug and the AE and the event could have been produced by the participant's clinical state or other modes of therapy administered to the participant.
3. **Possible**: The suspected AE may or may not follow a reasonable temporal sequence from study drug administration but seems to be the type of reaction that cannot be dismissed as unlikely. The event could have been produced or mimicked by the participant's clinical state or by other modes of therapy concomitantly administered to the participant.
4. **Likely**: The suspected adverse event follows a reasonable temporal sequence from study drug administration, abates upon discontinuation of the drug, and cannot be reasonably explained by the known characteristics of the participant's clinical state.
5. **Definitely related**: Reserved for those events which have no uncertainty in their relationship to test drug administration: this means that a re-challenge was positive.

The outcome of each SAE must be assessed at the same visit and according to the following classification:

Recovered: The participant recovered from the event with no residual problems.

Not yet recovered: This outcome can only be used for Serious Adverse Events. The event no longer meets a ‘Serious’ criterion, but medical event is not yet completely cured. The remaining event should be listed as a separate adverse event in the adverse event table, and with its own outcome.

Permanent damage: The event has resulted in permanent impairment.

Ongoing: the participant is continued to be followed for the event.

Death: The participant died. This term should only be used for the event which resulted in death. Any other events which were present at the time of death, but were not the cause of death, should be listed as ‘Ongoing’.

Unknown: The participant cannot be traced and no final outcome for the event could be determined.

There may be situations when an SAE has occurred, and the investigator has minimal information to include in the initial report to the Sponsor. However, it is very important that the investigator always makes an assessment of causality for every event prior to transmission of the report to the Sponsor. The investigator may change his/her opinion of causality in light of follow-up information, amending the SAE-report form accordingly.

7.2.4 Special Reporting Situation

All special reporting situations must be reported within 3 business days using the SAE or pregnancy form by email to:

Clinical Trials Unit
Institute of Tropical Medicine
Nationalestraat 155

B-2000 Antwerp – Belgium
Email: pharmacovigilance@itg.be

A study safety checklist SOP will be prepared describing all responsibilities and actions that are required in case of a special reporting situation.

An overview of all special reporting situations:

- Drug exposure during pregnancy (paternal, maternal)
- Suspected transmission of any infectious agent via administration of a drug under study.
- Overdose of a drug(s) under study*
- Exposure to a drug(s) under study from breastfeeding*
- Suspected abuse/misuse of a study product(s) under study*
- Inadvertent or accidental exposure to a study Product(s) under study*
- Any failure of expected pharmacological action (i.e., lack of effect) of a study Product(s) under study *
- Medication error (includes potential, intercepted or actual) involving a study product (with or without patient exposure to the Janssen Product(s) under study, e.g., name confusion)*
- Unexpected therapeutic or clinical benefit from use of a study Product(s) under study*

*** These special reporting situations only need to be reported to Janssen within 3 business days if the event is associated with an SAE. If the event is not associated with an SAE, the SS should be recorded in the CRF and sent annually to Janssen.**

7.2.5 Product Quality Complaints (PQC)

All PQC's must be reported within 3 business days using the PQC Notification Form by email to:

Clinical Trials Unit
Institute of Tropical Medicine
Nationalestraat 155
B-2000 Antwerp – Belgium
Email: pharmacovigilance@itg.be

Any complaint that indicates a potential quality issue during manufacturing, packaging, release testing, stability monitoring, dose preparation, storage or distribution of the product or delivery system is considered a PQC. Not all PQCs involve a patient.

A study safety checklist SOP will be prepared describing all responsibilities and actions that are required in case of an PQC.

Examples of PQC include but are not limited to:

- Mislabelling or misbranding

- Information concerning microbial contamination, including a suspected transmission of any infectious agent by a product
- Any significant chemical, physical, or other changes that indicate deterioration in the distributed product
- Any foreign matter reported to be in the product
- Mixed product, e.g., two drugs are mixed-up in the packaging process
- Incorrect tablet sequence (e.g., oral contraceptive tablets)
- Insecure closure with serious medical consequences, e.g., cytotoxics, child-resistant containers, potent drugs
- Suspected counterfeit or tampered product

- Adverse Device Effects including any adverse event resulting from insufficiencies or inadequacies in the instructions for use, the deployment, implantation, installation, operation, or any malfunction of a medical device or combination product. This also includes any event that is a result of a use error or intentional misuse and dosing device malfunctions (e.g., auto-injector button not working, needle detaching from syringe, etc.)
- Physical defect (e.g., abnormal product odour, broken or crushed tablets, etc.)

7.3 Surveillance for selection for mycobacterial drug resistance

The risk of inducing rifampicin resistance in (undiagnosed) tuberculosis or leprosy patients with one single dose of the drug is negligible.[40] As was described by Mitcheson for treatment of tuberculosis, resistance emerges as a result of selection of resistant mutants under conditions of monotherapy.[41] This requires high numbers of bacilli as observed in cavitary pulmonary tuberculosis, combined with prolonged exposure, as mycobacteria are slow to replicate. Repeated cycles of starting a treatment regimen, interrupting treatment, and restarting after some days can mimic monotherapy because of differential activity of drugs during the first days of treatment. However a single dose of rifampicin, possibly repeated once after four weeks, will not result in drug resistance. The BE-PEP regimen consists of two drugs, rifampicin and bedaquiline, further reducing the risk of selection of resistant mutants, although the half-life of bedaquiline and its metabolites far exceeds that of rifampicin.

To further decrease any potential risk of acquired drug resistance we will screen for signs and symptoms of pulmonary tuberculosis and exclude those participants from PEP.

We will also monitor molecular resistance for rifampicin and bedaquiline on samples from all leprosy patients, using an expanded Deeplex MycLep assay in which the atpE gene of *M. leprae* is included and using whole genome sequencing. Lastly, we will also recruit presumptive tuberculosis patients in Comoros for analysis of sputum and/or tongue swabs and genotype extracted *M. tuberculosis* DNA with the Deeplex MycTB XL, which includes additional targets potentially associated with bedaquiline resistance. Whole genome sequencing may be used to track the spread of drug resistant TB.

7.4 Other risks

Other risks to the participants are minimal. Leprosy patients identified will have a slit skin smear and a 4mm punch biopsy from the edge of the lesion taken (except if the lesion is in the face). The biopsy will be collected under local anesthesia. These procedures may cause slight pain, and carry a small risk of bleeding, which will be controlled by the person collecting these samples. The staff on the Comoros are already executing these procedures as part of the ongoing PEOPLE study.

8. STUDY MANAGEMENT

In the framework of the PEOPLE study, a bi-weekly meeting has been set-up for the purpose of the day-to-day management of the study and to discuss the progress of the trial. The meeting is attended by representatives of the sponsor and when required by representatives of the sites. These bi-weekly meetings will be continued within the BE-PEOPLE study. They will be organized by the clinical trials unit of ITM.

Also in the PEOPLE trial a Scientific Advisory Committee (SAC) has been established for the purpose of providing independent advice on the preliminary results that have been analyzed during the interim analyses. A charter detailing the modalities for this SAC has been drawn up. Members of the current SAC of the PEOPLE study will be asked to stay on as members of the SAC for BE-PEOPLE, if necessary additional members will be solicited. A new charter will be set-up and signed for BE-PEOPLE purposes.

For BE-PEOPLE, once Phase 2 study is finalized and after statical analysis is done, an independent Data Safety Monitoring Board (DSMB) will review the (S)AEs and outcomes of the Phase 2 study and advice on stopping versus proceeding to phase 3. The sample size chosen will allow for a precise estimate of the mean QTc and its variance. It will be left to the judgement of the DSMB whether any observed prolongation of QTc is clinically relevant and has implications for proceeding to Phase 3. Besides this, the Phase 2 study enrollment will be paused at any moment, if any of the below criteria is met. In this case a DSMB consultation will be held asap to discuss the further conduct of the trial. Enrollment and randomization should be paused until a DSMB decision is made, other follow-up visits can continue.

1. Death of a participant considered related to study drug
2. One or more participants experience an SAE or Gr 4 AE or a persistent (upon repeat testing) Gr 4 laboratory abnormality that is determined to be related to study drug
3. Three or more participants experience a Gr 3 or greater AE of the same type (as per medical judgement) that is determined to be related to study drug
4. Three or more participants experience a persistent (upon repeat testing) Gr 3 laboratory abnormality related to the same laboratory parameter and considered to be related to study drug
5. Two or more participants experience QTc > 500 ms after intake of the study medication
6. One or more participants has AST or ALT > 8x ULN, in absence of causative explanation, after intake of the study medication

During the phase 3 study the DSMB will also review active (S)AE follow-up on the day after study medication administration.

9. STATISTICAL ANALYSIS

The statistical analysis will be described in the statistical analysis plan (SAP), written by the biostatistician, which is binding and will be finalized before database lock or before any other analysis takes place.

9.1 Baseline characteristics:

Both an intention to treat (ITT) and a per-protocol (PP) approach will be used to define the analysis populations in both phase 2 and phase 3 studies. In the Intention-to-Treat analysis, all participants will be analyzed according to their randomized allocation, even in case they do not receive the randomized treatment, show protocol violations prior to or during the study.

In the per-protocol analysis only participants who received PEP as planned, have completed follow-up, and follow the protocol as planned are included.

In the phase 2 study, the PP population will be the primary analysis population as indicated for non-inferiority studies by the ICH-E9 guidelines. For the phase 3 study, the intention to treat will be the primary approach.

The number of participants screened and enrolled or excluded will be summarized according to reason for exclusion. Of the enrollees, the number of patients discontinued or lost to follow-up will be recorded by reason and time of discontinuation. These figures will be summarized in a CONSORT flow diagram.

Subjects in each treatment group will be described according to baseline characteristics. The description will be in terms of medians and interquartile ranges for continuous variables and using counts and percentages for categorical variables. Standard statistical tests of significance of imbalance in baseline characteristics will be performed.

9.2. Phase 2 study:

9.2.1. Primary analysis

The main objective about the difference in QTc between the two arms 24 hours after treatment administration will be assessed using a two-sample t-test.

9.2.2. Secondary analysis

Baseline frequency of ALT/AST elevations and QTc prolongations in the population, before and after study drug administration will be estimated using 95% confidence intervals for each time point and by study arm. No formal comparison will be done between time points or between arms.

Potentially frequent adverse events such as gastro-intestinal (nausea, vomiting), nervous system-related (headache, dizziness) and cutaneous reactions will be tabulated and presented using standard descriptive statistics. A comparison between the two arms will be done using Fisher's exact test.

9.3. Phase3 study

9.3.1. Primary analysis

The main analysis will be to assess the effect of BE-PEP at individual level. Because anyone living within 100 meter of an index case over the period 2018-2023 will be eligible for PEP and if this is more than 50% of the village population the entire village population above 2 years of age will be eligible, we assume that in the first year there will be blanket coverage with either SDR-PEP or BE-PEP. We will fit a Poisson model adjusted for follow-up time as an offset term with village nested in island as random effect and type of PEP (BE-PEP or SDR-PEP), as explanatory variable. Those who did not receive PEP and those below the age or weight limits for BE-PEP will be excluded from this analysis, SDR-PEP will be our reference category. Outcome variable will be incident leprosy. We will consider all cases diagnosed after the first intervention round in 2023, until the final survey in 2026.

9.3.2 Secondary analyses

In a second analysis the we will calculate the incidence rate ratio of leprosy at village level between arms 1 and 2. Here all participants enrolled will be taken into account, even those that did not receive PEP. A similar model as in the primary analysis will be used, including all persons in the villages randomized, even those who were not eligible to receive the study treatment due to age or weight restrictions.

Prespecified adverse events such as gastro-intestinal (nausea, vomiting), nervous system-related (headache, dizziness) and cutaneous reactions will be tabulated and presented using counts and percentages. Comparisons between study arms will be done using Fisher's exact test.

In the villages that were originally allocated to arm 4 of the PEOPLE trial, we will do one final anti-PGL1 sero-survey in 2026. Based on the comparison of age specific anti-PGL-I sero-prevalence rates at the time of the 2019 survey and in the final survey in 2026, we will assess the potential of anti-PGL-I surveys as a tool to monitor leprosy transmission. We will compare seroprevalence rates in different age groups of 2019 to 2026, both overall and by village and island to assess whether a trend can be identified and whether this trend is consistent across villages/islands. This analysis will be primarily descriptive.

Cost data will be gathered throughout the study and used to calculate the average cost per person screened for leprosy by island, average cost per case detected by island and average cost per case of leprosy averted by study arm and by island. Incremental costs will be calculated using the SDR arm (arm 2) as baseline.

Prevalence of bedaquiline and/or rifampicin resistant strains will be calculated per island with in the numerator all leprosy patients with rifampicin or bedaquiline resistant isolates identified and in the denominator all patients tested. Along with the proportions 95% confidence intervals using the Wilson's method will be calculated.

Results of genotyping of *M.leprae* will be analyzed separately in the framework of the project titled: 'Improving leprosy prevention strategies by integrating social network analysis with spatial and molecular epidemiology data of Mycobacterium leprae in the Comoros'.

9.3.3 Interim analyses

An interim analysis will be conducted upon completing the second door-to-door survey after randomization, in early 2025. This analysis will include both the individual effect of PEP and the effect of PEP at village level, as described above.

10. Monitoring And Quality Assurance

The clinical research scientist of CTU will perform on-site monitoring visits, with the purpose of source document verification and follow-up of local study implementation in line with ICH-GCP guidelines (including e.g. investigator file completeness). The monitoring activities and frequency will be described in a monitoring plan. A report detailing the actions taken during the monitoring visit will be written and shared amongst the study team.

The coordinating investigator will also visit the study team at regular intervals for project coordination and follow-up.

A pharmacist in Comoros has been trained on GCP and will serve as local quality responsible IP management such as completing inventory- and temperature log monitoring). The PI and involved site research staff will allocate adequate time and resources for such monitoring activities.

The sponsor will inform the Investigators concerned immediately upon notification of a pending study centers inspection by any regulatory authority or funder. Likewise, the investigator will inform the sponsor of any pending inspection.

11. Data Management

In the BE-PEOPLE study we will continue to use formats developed in the PEOPLE study consisting of a paper survey form, four forms in in Android app and an Access database with barcode, name, age and gender of each participant enrolled. After the baseline survey in 2022, which partly overlaps with the final survey of PEOPLE, we will shift from ODK Collect used in the PEOPLE trial to REDCap, which is GCP compliant. REDCap will also be used in the phase 2 study in 2022.

Data Management will be performed by the CTU data managers in collaboration with the Principal Investigator and with the study staff involved in collecting and handling the study data. For the continued follow-up of the cohort of leprosy patients established during the PEOPLE trial and the preceding ComLep trial, of which drug resistance surveillance is a part, we will continue with the existing system. Drug resistance surveillance will be extended to tuberculosis patients, for this purpose a separate database will be developed.

A separate Data Management Plan will be prepared, with following essential aspects:

11.1 Data collection, handling and retention

The phase 2 study organized in 2022 will consider the collecting and managing of participant-level data comprising medical data and in particular safety data referring to adverse events, medical history, concomitant medication, ECG and liver function tests.

The phase 3 study organized from 2023 till 2026 will consider the collecting and managing of household-level data (Household ID, geographic coordinates) and participant-level data (participant ID, demographic, health and medical data with reference to medical history, concomitant medication, examinations, leprosy and TB, treatments, registration of PEP).

Both studies will have their own database and be based on a system of data collection and management via electronic questionnaires on mobile devices (smartphones, tablets and/or laptops), by using REDCap.

REDCap (Research Electronic Data Capture) is a software widely used in the academic research community for building and managing surveys, databases and research studies. It is a secure web application as well as mobile application that can be used to collect, clean and manage subjects' information in accordance with various standards and applicable regulations (GCP, CFR 21 part 11, HIPAA). Furthermore, the software features amongst others a query management system and capabilities for importing and exporting data in various forms. REDCap allows for offline data collection in remote places. When connecting to the internet or cellular networks, data from the mobile device can be synchronized with the study server where the study data will be securely retained.

Paper survey forms will be used if deemed necessary (e.g. as a backup when having issues with the electronic device). Only data defined by the study protocol will be collected.

Questionnaires will be tested and validated before the household visits start. All study personnel will be trained on informed consent and study procedures, data collection and entry before the study start.

Edit checks and branching logic, programmed onto the electronic forms will validate at the point of data entry and support quality data. In addition, the data will be reviewed and monitored during the course of the study by various study collaborators. Data cleaning and querying of inconsistent, inaccurate and missing data will be done following a Data Validation Plan to achieve a timely database lock.

The following data will be entered in the eCRF via direct data entry:

- AE’s
- Special reporting Situations (except if reported via SAE/pregnancy report)
- Inclusion/exclusion criteria/eligibility
- Clinical examination
- Concomitant medication
- Medical history
- Visit confirmation
- Study medication administration
- End of study

11.2 Data security and confidentiality

The data management/IT system that will be used includes a robust security architecture including encryption, firewalls, antivirus software and controlled user access (smartphone/tablet authentication, username, personal password and authorized user role). A list with all users who have access to the system will be kept updated during the study. A daily backup of data will be provided at the ITM server.

Both the sponsor and the study sites will see to it that the necessary measures are taken to ensure that all confidential documentation and critical devices (study Data Management Plan, procedures, completed questionnaires) and IT devices and equipment (mobile devices, computers, server) are kept secure (closed cupboards, closed and/or badge controlled offices and/or rooms).

Private data of the study participants will be handled confidentially. Household addresses, geographic coordinates of households, participant name and other contact data for each household or participant will be kept separate and limited to authorized staff at the study site(s) only. For analysis purposes household addresses or geographic coordinates, participant names and contact data will be replaced by a specific study household and subject identification code (=pseudonym).

The funder will be provided (i) de-identified study data for the Phase 2 interim analysis, including, but not limited to, interim analysis data outputs as provided to the study’s Data Safety Monitoring Board (DSMB) as well as the interim study report and (ii) de-identified data from the Phase 3 trial, including but not limited to annual reports, final study report, open-access publications, and other de-identified open-access safety and efficacy data suitable for third-party sharing, but will otherwise not have access to the study data.

11.3 Data protection and security

This study will be performed in compliance with the European General Data Protection Regulation. The sponsor will also be the data controller and the lawful ground for the personal data processing of participants will be the public interest, because the study has got a potentially big impact for the public health policies regarding leprosy and TB treatment and strategies. The controller also has got a public mandate, responsibility and expertise to perform scientific research in this area and the personal data will only be shared with involved non-commercial stakeholders such as the MoH, NTLCP and – taking privacy restrictions into account - will be made openly accessible as much as possible.

Considering that the study will collect special categories of personal data such as medical data on a large scale, including data from minors and vulnerable subjects or populations, a Data Protection Impact Assessment will be performed prior to the start of the data processing.

The data uploaded to the server will be barcoded, without personal identifiers. The database containing names and barcodes of participants will remain on the Comoros.

Appropriate organizational and technical measures will be implemented for all parties involved and all applications and software systems used for the study. This will be further elaborated in the Data Management Plan and DPIA. The ITM Data Protection Officer will be involved to perform such proper assessments.

11.3 Filing and archiving

All essential documents for the trial will be kept in paper format in the Investigator Files at the study sites. Each Principal Investigator is responsible for completeness and ensuring a secure and appropriate location for storage of the Investigator Files and any other study related documentation present at sites, as well as for ensuring that only site staff that is competent and delegated to work for the study have got access to the files.

After study completion, all the relevant study documentation should be retained for a period of 20 years or in accordance with the local legislation. ITM should be informed prior to destruction of study files.

The Investigator Files should at all times remain available for internal audits and/or inspections of regulatory authorities, also after completion of the project.

The Trial Master File and any other sponsor related documentation will be archived at ITM at a secure and appropriate location and will only be accessible for competent and delegated sponsor staff. After completion of the study, the Trial Master File will remain available for internal audits and/or inspections of regulatory authorities for a period of 20 years or according to regulatory requirements.

12. ETHICAL ISSUES

12.1 Ethics and regulatory review

The study will be submitted for review and approval to the 'Comité National d'Ethique pour les Sciences de la Vie et de la Santé' (CNESS) as well as to the 'Agence NAtionale des Medicaments et des EVacuations sanitaires' (ANAMEV) in Comoros. As ITM is the sponsor of this project, approval by the Institutional Review Board (IRB) of the ITM will also be requested. In addition, the study will be submitted for approval to the Ethics Committee (EC) of the University of Antwerp Hospital in Antwerp. Potential comments from all these review boards will be addressed, the same applies to comments from the selected collaborating partners. No participants will be enrolled or participant related activities performed before written approval from all these bodies in Belgium and in the study country concerned is obtained. Any substantial amendment to the study (documents) will also require approval from the above mentioned bodies. A yearly update on the status of the study will be provided as required. The study will be carried out according to the principles stated in the Declaration of Helsinki, GCP, GDPR and all applicable regulations and according to established international scientific standards.

Prior to the start, this study will also be included in the Clinicaltrials.gov public registry.

12.2 Protocol amendments

Once the final clinical study protocol has been issued and signed by the authorized signatories, it cannot be informally altered. Protocol amendments have the same legal status and must pass through the appropriate steps before being implemented. Any substantial change must be approved by all the bodies and EC's that have approved the initial protocol, prior to being implemented, unless it is due

to participant's safety concerns (in which case the immediate implementation can be necessary for the sake of participant's protection. In case modifications to the protocol or amendment are requested by any local EC/CA during the review process, these must be discussed and agreed upon with the Sponsor prior to any resubmission incorporating those changes.

12.3 Informed consent

In the villages that are already part of the PEOPLE trial, we will ask verbal informed consent for a revisit in 2023 as well as for use in further research of the data collected during the PEOPLE trial. This information will be entered in the electronic data collection form used in the final screening round of PEOPLE. For baseline screening in the new villages, not included in the PEOPLE trial, we will ask written informed consent. This consent will be collected on a survey form of which there will be one per household. At the start of the intervention trial (phase 3) in 2023 we will again seek informed consent from all study subjects. This time written informed consent will be collected individually on separate ICF forms, for each subject. In Redcap the date of signing consent will be documented via direct data entry.

All participating household members, as well as the legal guardian and witness, as applicable, will then be asked to sign for participation in the study on individual informed consent forms. For the baseline survey we will continue to use a form per household with a separate line for each household member as currently practiced in the PEOPLE trial. The forms will be stored by the study team in a secure manner.

Written informed consent will be obtained before any study specific procedure is performed. For the sample collection for incident leprosy cases identified during the yearly screening, a separate consent will be obtained. Also for (presumptive) TB patients informed consent will be obtained to test sputum samples for resistance to rifampicin and bedaquiline, for this purpose there will be a separate ICF.

Participants who provide samples (i.e. index cases, (presumptive) tuberculosis patients and inhabitants of villages randomized to arm 4 of the original PEOPLE trial) will be informed that their samples will be stored for 20 years and may be used for other research related to leprosy and/or tuberculosis in the future. They will also be informed that these samples can be shipped abroad.

The legal age of consent in in the Comoros is 18. For participants, between 2 and 18, informed consent of the parents or guardian will be sought. However, as of the age of 12, additional signed assent from the minor is needed before he/she can participate in the study. However, for female or male participants under 18 years old who are married (or already divorced), the participant has the right to give consent him/herself without informed consent of a parent or guardian, if the local EC in the Comoros approves.

The informed consent interviews will be conducted in the preferred language of the patients by a qualified person formally delegated by the PI. Informed consents will be available in French. Comorian is a rather spoken language and therefore the ICF will be orally translated by the interviewer on-site. Translations will be performed by a native speaker with thorough knowledge of the French language and checked for consistency by another native speaker. A recorded translation will be provided to ensure consistency and completeness of delivered information across different interviewers.

In case participants are illiterate, an independent witness will be sought to ensure that the participants receive complete information. In such case, the participant will thumbprint the consent form and the witness will add his/her signature.

12.4 Confidentiality

Incident leprosy cases will not be made publicly known outside the household to avoid stigmatization. Data for the study will be collected by the study team via an Android application, in which participants will be identified by their geographical location and a barcode. The data will be uploaded regularly to a secure ITM server. Upon uploading to the ITM server, data will automatically be deleted from the Android device. Names will be recorded on the survey form, as is required for management purposes by the NTLCP which is responsible for follow-up and treatment of all leprosy and TB suspects or cases identified. The names will be linked to the data collected in the Android app through a barcode. For the final analysis a dataset without names will be used.

The Informed Consent Forms and survey forms with the participant names will be stored in a secured location in a lockable cabinet with access limited to country PI's only. The Access database with survey form data will be managed and secured by the NTLCP's.

Any other forms and reports needed for the study will be pseudonymised and can only be linked to the study participant by barcode.

Participant study information will not be released to anybody outside the medical team, except as necessary (and under confidentiality agreement) for the purpose of monitoring, auditing and inspection by competent authorities.

During or after final publication of results, individual level participant data might be shared for secondary research purposes in an anonymized or pseudonymised manner and by means of a controlled data access procedure and after the signing of a data sharing agreement.

12.5 Potential Risks

In this study, participants will (depending on the arm their village is randomized in) receive a single dose of 10mg/kg of rifampicin or a single dose of 10mg/kg rifampicin plus 400 or 800mg of bedaquiline depending on age and weight. Because bedaquiline has not been registered for treatment or prophylaxis of leprosy and because we will be using it in combination with rifampicin, stringent safety procedures have been put in place. *we have asked the advice from the Belgian regulatory authority for pharmaceuticals (FAGG) who have given an advise with different, detailed recommendations*. The study has been split into two phases, a phase 2 study to reconfirm safety of the proposed BE-PEP regimen and a phase 3 study that is conditional upon a positive advice from an independent DSMB, taking into account the results of the phase 2 study.

For rifampicin the dosing and indication are fully adherent to WHO recommendations. Safety of bedaquiline has been established for a single dose of 800 mg as well as in EBA studies in which 700mg on the first day was followed by 500mg on the second day. QT prolongation is not expected after single dose administration.

The Covid-19 pandemic has so far had limited impact in Comoros although a sudden upsurge in early 2021, in particular on the island of Mohéli, caused the cumulative death toll to increase from 10 at the beginning of the year to 144 by early March. Since then there have been few additional cases and only 5 additional deaths to date. So far approximately 30% of the population have been fully vaccinated, health workers have been prioritized. Door-to-door surveys for the PEOPLE study have been interrupted on Mohéli and resumed on April 1st. While in the villages, PEOPLE study teams wear surgical masks and gloves. All survey staff, both from the central team and those recruited at village level, have been fully vaccinated. As long as required during the BE-PEOPLE study wearing of masks and gloves will be continued. An early warning system established as part of the PEOPLE study in

which health workers were tested for antibodies on a regular basis has now been abandoned because almost all health workers are vaccinated, moreover sufficient testing capacity is now in place on all three islands.

The risk of loss of confidentiality is minimized by targeting for screening entire villages/hamlets, thus presence of the team cannot be interpreted as presence of leprosy patients in a household. Screening will be for skin conditions in general and for signs of pulmonary tuberculosis. Distribution of PEP to neighborhood contacts will be done upon completion of the door-to-door survey in the village. However since we are using a 100 meter common perimeter around all leprosy cases identified, the numbers of households included will always be large. If more than 50% of households are to be included, we will include the entire village. Unlike in an approach where PEP would be provided to neighbors, or neighbors of neighbors, in the approach used in this trial it will be almost impossible to deduce where the source case was located. Other potential breaches in confidentiality are minimized by GDPR compliant data management. A Data Protection Impact Assessment will be conducted to assess potential data protection risks and to define actions on how to mitigate those risks.

At a higher level there could be a risk of villages or neighborhoods being stigmatized because of being involved in the study. This risk is not very high because leprosy is widespread in Comoros. In the areas targeted most villages are known to be affected. On Anjouan since 2008 and more recently also on Mohéli, villages are regularly visited by leprosy control program teams for active case finding activities. In these villages such problems have never occurred. The fact that in the BE-PEOPLE trial even more villages will be included further reduces the risk of entire villages being stigmatized.

Capillary blood sampling by finger-prick will be performed on a subset of participants at the time of the final survey round in 2028. No side effects apart from minor discomfort are to be expected from these tests.

Skin biopsy and slit skin smear require making a small incision in the skin and taking a superficial skin sample. Local anesthesia will be applied to reduce the discomfort for the participants to a minimum.

12.6 Benefits

The yearly door-to-door screening as part of the study yields the greatest advantage of the study for the people living in the study target area, as this will guarantee early detection of leprosy and consequently early treatment. For people living in close proximity of a leprosy case, the study is expected to reduce transmission of leprosy due to PEP being offered through the study.

12.7 Compensation for participation

All tests and procedures that are part of this study are free of charge for the participants. Also treatment for leprosy and for minor skin conditions is offered free of charge by the national leprosy control programs, but are not part of this study. On the day of pre-recruitment, day 1 and day 14 (visits at the study house), a compensation will be provided to the participants. In case of a blood draw at the study house during visit D30, an additional compensation will be provided to the participant. This compensation will consist of a food package per visit (5kg rice, 5 milk sachets, 6 boxes of sardines with a total value of 10 euros on average). In case of participant referrals due to potential adverse events, transport costs will be reimbursed by the study.

12.8 Insurance

Prior to the start of the trial, ITM as sponsor of the trial will obtain a no-fault study insurance to cover any injury, damage or loss to study participants which is caused directly or indirectly by their

participation in the study. Participants will be informed of such insurance in the Participant Information Sheet.

13. DISSEMINATION OF RESULTS, INTELLECTUAL PROPERTY

All study documents are provided by the Sponsor to the Investigators and his/her appointed staff in confidence. None of this material may be disclosed to any party not directly involved with the study, without written permission from the Sponsor and other partners as described in the MTA and study agreement.

The results of this study will be presented in a scientific articles submitted to peer- reviewed journals. They will also be presented in other fora such as the Leprosy Research Initiative (LRI) meeting.

14. ARCHIVING

The sponsor and Investigator must maintain adequate and accurate records to enable the conduct of the study to be fully documented and the study data to be verified. The relevant (essential) documents are those documents which individually and collectively permit to assess the conduct of the trial, the quality of the data produced and the compliance with GCP standards and applicable regulatory requirements.

All the relevant study documentation present at all partners involved should be retained for a minimum of twenty years, unless differently requested by national authorities. The Sponsor should be informed prior to destruction of the files. After completion of the study, the IF will remain available for internal audits and/or inspections of regulatory authorities for a period of twenty years, unless differently requested by national authorities.

15. References

1. WHO. Guidelines for the Diagnosis, Treatment and Prevention of Leprosy. 2018.
2. Cartel JL, Chanteau S, Boutin JP, Taylor R, Plichart R, Roux J, et al. Implementation of chemoprophylaxis of leprosy in the Southern Marquesas with a single dose of 25 mg per kg rifampin. Int J Lepr Other Mycobact Dis. 1989;57(4):810-6. Epub 1989/12/01. PubMed PMID: 2809347.
3. Nguyen LN, Cartel JL, Grosset JH. Chemoprophylaxis of leprosy in the southern Marquesas with a single 25 mg/kg dose of rifampicin. Results after 10 years. Lepr Rev. 2000;71 Suppl:S33-5; discussion S5-6. Epub 2001/02/24. doi: 10.5935/0305-7518.20000064. PubMed PMID: 11201884.
4. Pattyn SR, Groenen G, Janssens L, Kuykens L, Mputu LB. A controlled therapeutic trial in paucibacillary leprosy comparing a single dose of rifampicin with a single dose of rifampicin followed by one year of daily dapsone. The Collaborative Study Group for the Treatment of Leprosy in Zaire. Lepr Rev. 1991;62(2):179-85. Epub 1991/06/01. doi: 10.5935/0305-7518.19910021. PubMed PMID: 1870380.
5. Wendt WR. [Recuperation cure for schoolchildren indication and own experience (author's transl)]. Offentl Gesundheitswes. 1978;40(2):79-83. Epub 1978/02/01. PubMed PMID: 147428.
6. WHO. WHO Operational Handbook on Tuberculosis, Module 4: Treatment - Drug-Resistant Tuberculosis Treatment 2020. Available from: https://www.who.int/publications/i/item/9789240006997.
7. Ortuno-Gutierrez N, Mzembaba A, Ramboarina S, Andriamira R, Baco A, Braet S, et al. Exploring clustering of leprosy in the Comoros and Madagascar: a geospatial analysis. Int J Infect Dis. 2021. Epub 2021/05/16. doi: 10.1016/j.ijid.2021.05.014. PubMed PMID: 33991682.
8. Bakker MI, Hatta M, Kwenang A, Van Benthem BH, Van Beers SM, Klatser PR, et al. Prevention of leprosy using rifampicin as chemoprophylaxis. Am J Trop Med Hyg. 2005;72(4):443-8. Epub 2005/04/14. PubMed PMID: 15827283.
9. Leprosy/Hansen disease: Contact tracing and post-exposure prophylaxis. World Health Organization. Delhi2020 [14 October 2021]. Available from: https://apps.who.int/iris/handle/10665/336679.
10. Araujo S, Freitas LO, Goulart LR, Goulart IM. Molecular Evidence for the Aerial Route of Infection of Mycobacterium leprae and the Role of Asymptomatic Carriers in the Persistence of Leprosy. Clin Infect Dis. 2016;63(11):1412-20. Epub 2016/08/26. doi: 10.1093/cid/ciw570. PubMed PMID: 27558564.
11. Hansen G H, A,. Undersøgelser Angående Spedalskhedens Årsager (Investigations concerning the etiology of leprosy in Norwegian). Norsk Mag Laegervidenskaben 1874;(4):88. 1874;4:88.
12. Schuenemann VJ, Singh P, Mendum TA, Krause-Kyora B, Jager G, Bos KI, et al. Genome-wide comparison of medieval and modern Mycobacterium leprae. Science. 2013;341(6142):179-83. Epub 2013/06/15. doi: 10.1126/science.1238286. PubMed PMID: 23765279.
13. Smith CM, Smith WC. Chemoprophylaxis is effective in the prevention of leprosy in endemic countries: a systematic review and meta-analysis. MILEP2 Study Group. Mucosal Immunology of Leprosy. J Infect. 2000;41(2):137-42. Epub 2000/10/12. doi: 10.1053/jinf.2000.0698. PubMed PMID: 11023757.
14. WHO. World Health Assembly 44. Resolutions and decisions. Leprosy: World Health Organization 1991. . World Health Assembly1991.
15. Smith WC, van Brakel W, Gillis T, Saunderson P, Richardus JH. The missing millions: a threat to the elimination of leprosy. PLoS Negl Trop Dis. 2015;9(4):e0003658.

Epub 2015/04/24. doi: 10.1371/journal.pntd.0003658. PubMed PMID: 25905706; PubMed Central PMCID: PMCPMC4408099.
16. WHO. ANNEX 4 TB burden estimates, notifications and treatment outcomes for individual countries and territories, WHO regions and the world. 2018. Available from: https://www.who.int/tb/publications/global_report/gtbr2018_annex4.pdf.
17. Moet FJ, Pahan D, Oskam L, Richardus JH, Group CS. Effectiveness of single dose rifampicin in preventing leprosy in close contacts of patients with newly diagnosed leprosy: cluster randomised controlled trial. BMJ. 2008;336(7647):761-4. Epub 2008/03/12. doi: 10.1136/bmj.39500.885752.BE. PubMed PMID: 18332051; PubMed Central PMCID: PMCPMC2287265.
18. Ortuno-Gutierrez N, Younoussa A, Randrianantoandro A, Braet S, Cauchoix B, Ramboarina S, et al. Protocol, rationale and design of PEOPLE (Post ExpOsure Prophylaxis for LEprosy in the Comoros and Madagascar): a cluster randomized trial on effectiveness of different modalities of implementation of post-exposure prophylaxis of leprosy contacts. BMC Infect Dis. 2019;19(1):1033. Epub 2019/12/07. doi: 10.1186/s12879-019-4649-0. PubMed PMID: 31805862; PubMed Central PMCID: PMCPMC6896699.
19. Boeree MJ, Diacon AH, Dawson R, Narunsky K, du Bois J, Venter A, et al. A dose-ranging trial to optimize the dose of rifampin in the treatment of tuberculosis. Am J Respir Crit Care Med. 2015;191(9):1058-65. Epub 2015/02/06. doi: 10.1164/rccm.201407-1264OC. PubMed PMID: 25654354.
20. Diacon AH, Dawson R, Von Groote-Bidlingmaier F, Symons G, Venter A, Donald PR, et al. Randomized dose-ranging study of the 14-day early bactericidal activity of bedaquiline (TMC207) in patients with sputum microscopy smear-positive pulmonary tuberculosis. Antimicrob Agents Chemother. 2013;57(5):2199-203. Epub 2013/03/06. doi: 10.1128/AAC.02243-12. PubMed PMID: 23459487; PubMed Central PMCID: PMCPMC3632959.
21. Pieterman ED, Sarink MJ, Sala C, Cole ST, de Steenwinkel JEM, Bax HI. Advanced Quantification Methods To Improve the 18b Dormancy Model for Assessing the Activity of Tuberculosis Drugs In Vitro. Antimicrob Agents Chemother. 2020;64(7). Epub 2020/04/29. doi: 10.1128/AAC.00280-20. PubMed PMID: 32340993; PubMed Central PMCID: PMCPMC7318015.
22. Wallis RS, Good CE, O'Riordan MA, Blumer JL, Jacobs MR, Griffiss JM, et al. Mycobactericidal activity of bedaquiline plus rifabutin or rifampin in ex vivo whole blood cultures of healthy volunteers: A randomized controlled trial. PLoS One. 2018;13(5):e0196756. Epub 2018/05/03. doi: 10.1371/journal.pone.0196756. PubMed PMID: 29718967; PubMed Central PMCID: PMCPMC5931679.
23. Winter H, Egizi E, Murray S, Erondu N, Ginsberg A, Rouse DJ, et al. Evaluation of the pharmacokinetic interaction between repeated doses of rifapentine or rifampin and a single dose of bedaquiline in healthy adult subjects. Antimicrob Agents Chemother. 2015;59(2):1219-24. Epub 2014/12/17. doi: 10.1128/AAC.04171-14. PubMed PMID: 25512422; PubMed Central PMCID: PMCPMC4335826.
24. Monitoring and epidemiological assessment of mass drug administration in the global programme to eliminate lymphatic fi lariasis: a manual for national elimination programmes 2011. Available from: https://apps.who.int/iris/handle/10665/44580.
25. Moura RS, Calado KL, Oliveira ML, Buhrer-Sekula S. Leprosy serology using PGL-I: a systematic review. Rev Soc Bras Med Trop. 2008;41 Suppl 2:11-8. Epub 2008/01/01. doi: 10.1590/s0037-86822008000700004. PubMed PMID: 19618069.
26. Braet S, Vandelannoote K, Meehan CJ, Brum Fontes AN, Hasker E, Rosa PS, et al. The Repetitive Element RLEP Is a Highly Specific Target for Detection of Mycobacterium

leprae. J Clin Microbiol. 2018;56(3). Epub 2018/01/07. doi: 10.1128/JCM.01924-17. PubMed PMID: 29305545; PubMed Central PMCID: PMCPMC5824061.
27. Buhrer-Sekula S, van Beers S, Oskam L, Lecco R, Madeira ES, Dutra MA, et al. The relation between seroprevalence of antibodies against phenolic glycolipid-I among school children and leprosy endemicity in Brazil. Rev Soc Bras Med Trop. 2008;41 Suppl 2:81-8. Epub 2008/01/01. doi: 10.1590/s0037-86822008000700017. PubMed PMID: 19618082.
28. van Beers S, Hatta M, Klatser PR. Seroprevalence rates of antibodies to phenolic glycolipid-I among school children as an indicator of leprosy endemicity. Int J Lepr Other Mycobact Dis. 1999;67(3):243-9. Epub 1999/11/27. PubMed PMID: 10575403.
29. Vink AS, Neumann B, Lieve KVV, Sinner MF, Hofman N, El Kadi S, et al. Determination and Interpretation of the QT Interval. Circulation. 2018;138(21):2345-58. Epub 2018/12/21. doi: 10.1161/CIRCULATIONAHA.118.033943. PubMed PMID: 30571576.
30. (2020) RCT. In: R Foundation for Statistical Computing V, Austria, editor. R: A language and environment for statistical computing. 4.0.2 ed.
31. Cohen K, Meintjes G. Management of individuals requiring antiretroviral therapy and TB treatment. Curr Opin HIV AIDS. 2010;5(1):61-9. doi: 10.1097/COH.0b013e3283339309. PubMed PMID: 20046149; PubMed Central PMCID: PMCPMC2936963.
32. Donald PR, Maritz JS, Diacon AH. The pharmacokinetics and pharmacodynamics of rifampicin in adults and children in relation to the dosage recommended for children. Tuberculosis (Edinb). 2011;91(3):196-207. doi: 10.1016/j.tube.2011.02.004. PubMed PMID: 21429802.
33. Shitara Y. Clinical importance of OATP1B1 and OATP1B3 in drug-drug interactions. Drug Metab Pharmacokinet. 2011;26(3):220-7. Epub 2011/02/08. doi: 10.2133/dmpk.DMPK-10-RV-094. PubMed PMID: 21297316.
34. WHO. Guidelines for the diagnosis, treatment and prevention of leprosy. [Document on the internet]. 2018. 28 June, 2018:[Available from: http://www.searo.who.int/entity/global_leprosy_programme/approved-guidelines-leprosy-executives-summary.pdf?ua=1.
35. Borisov SE, Dheda K, Enwerem M, Romero Leyet R, D'Ambrosio L, Centis R, et al. Effectiveness and safety of bedaquiline-containing regimens in the treatment of MDR- and XDR-TB: a multicentre study. Eur Respir J. 2017;49(5). Epub 2017/05/23. doi: 10.1183/13993003.00387-2017. PubMed PMID: 28529205.
36. Schnippel K, Ndjeka N, Maartens G, Meintjes G, Master I, Ismail N, et al. Effect of bedaquiline on mortality in South African patients with drug-resistant tuberculosis: a retrospective cohort study. Lancet Respir Med. 2018;6(9):699-706. Epub 2018/07/14. doi: 10.1016/S2213-2600(18)30235-2. PubMed PMID: 30001994.
37. Tanneau L, Svensson EM, Rossenu S, Karlsson MO. Exposure-safety analysis of QTc interval and transaminase levels following bedaquiline administration in patients with drug-resistant tuberculosis. CPT Pharmacometrics Syst Pharmacol. 2021. Epub 2021/10/10. doi: 10.1002/psp4.12722. PubMed PMID: 34626526.
38. FDA. Drug Development and Drug Interactions | Table of Substrates, Inhibitors and Inducer 2020. Available from: https://www.fda.gov/drugs/drug-interactions-labeling/drug-development-and-drug-interactions-table-substrates-inhibitors-and-inducers#table5-2.
39. Azram A AN, Leese L, Brigham M, Bowes R, Wheatcroft SB, Ngantcha M, Stegemann B, Crowther G, Tayebjee MH. Clinical validation and evaluation of a novel sixlead handheld electrocardiogram recorder compared to the 12-lead electrocardiogram in unselected cardiology patients (EVALECG Cardio). Eur Heart J. 2021. Epub 2021/09/28. doi: 10.1093/ehjdh/ztab083.

40. Mieras L, Anthony R, van BW, Bratschi MW, van den Broek J, Cambau E, et al. Negligible risk of inducing resistance in Mycobacterium tuberculosis with single-dose rifampicin as post-exposure prophylaxis for leprosy. Infect Dis Poverty. 2016;5(1):46. doi: 10.1186/s40249-016-0140-y [doi];10.1186/s40249-016-0140-y [pii].
41. Mitchison DA. How drug resistance emerges as a result of poor compliance during short course chemotherapy for tuberculosis. Int J Tuberc Lung Dis. 1998;2(1):10-5.

16. LIST OF ABBREVIATIONS

AE	Adverse Event
ALLEA	All European Academies
Anti-PGL-I	Anti-phenolic glycolipid-I
AR	Adverse Reaction
BCG	Bacille de Calmette Guérin
bedaquiline	Bedaquiline
BS	Blood Samples
CFZ	Clofazimine
CRF	Case Report Form
CRS	Clinical Research Scientist
CTU	Clinical Trial Unit
DF	Damien Foundation
DSMB	Data and safety monitoring board
EC	Ethics Committee
EDCTP	European and Developing Countries Clinical Trial Partnership
ESF	European Science Foundation
FSN	Form serial number
GC(L)P	Good Clinical (Laboratory) Practice
HGDI	Hunter-Gaston Discriminatory Index
HH	Household
IRB	Institutional Review Board
ITM	Institute of Tropical Medicine
LRI	Leprosy Research Initiative
LUMC	Leidsch Universitair Medisch Centrum
MB	Multi bacillary
NS	Nasal swabs
NTLCP	National Tuberculosis and Leprosy Control Program
ODK	Open Data Kit
PEOPLE	Post ExpOsure Prophylaxis for LEprosy in the Comoros and Madagascar
PEP	Post Exposure Prophylaxis
PI	Principal Investigator
PTB	Pulmonary Tuberculosis
qPCR	Quantitative Polymerase Chain Reaction
RIF	Rifampicin
SAC	Scientific Advisory Committee
SAE	Serious Adverse Event
SB	Skin Biopsy
SDDR-PEP	Single Double Dose Rifampicin Post-Exposure Prophylaxis
SDR	Single Dose Rifampicin
SSS	Slit skin smears
TMG	Trial Management Group
ZN	Ziehl-Neelsen

17. ANNEX 1: FAGG opinion to the protocol
