# Supplementary material for: Safety of single-dose bedaquiline combined with rifampicin for leprosy post-exposure prophylaxis: A Phase 2 randomized non-inferiority trial in the Comoros Islands
Source: PLoS Med. 2024 Oct 21;21(10):e1004453. doi: 10.1371/journal.pmed.1004453 (PMC11534270; doi:10.1371/journal.pmed.1004453)
Supplement: S1 Table — *This p-value tests the (unilateral) hypothesis that BE-PEP causes more QTc prolongation than SDR-PEP; a value below 0.025 means that this hypothesis is rejected. BE-PEP = post-exposure prophylaxis (PEP) with bedaquiline 800 mg + rifampicin 600 mg, SDR-PEP = PEP with rifampicin 600 mg only, ms = milliseconds. Regr coeff = Regression coefficient, Ref = reference. (DOCX) [file pmed.1004453.s004.docx]

**Table S1.** ECG results (QTc) in ms by allocation arm and age-group in Intention to treat analysis.

| **Age-group** | **Day** | **BE-PEP**  Mean (95% CI; min, max) | **SDR-PEP**  Mean (95% CI; min, max) | **Difference**  (BE-PEP – SDR-PEP; 95% CI) | **P-value for non-inferiority*** |
| --- | --- | --- | --- | --- | --- |
| **All ages** | 0 | 393 (390, 395;346, 440) | 392 (389, 395;331, 449) | 0.8 (-2.6, 4.2) | 0.70 |
|  | 1 | 396 (393, 399;345, 450) | 394 (391, 397;353, 440) | 2.4 (-1.1, 5.9) | 0.26 |
| *Regression analysis* | | | | | |
| Effect of BE-PEP on QTc  (unadjusted for baseline QTc values) | Day 1 | 2.39(-1.78 - 6.56) | Day 0 (Ref) |  | < 0.001 |
| Effect of BE-PEP on QTc  (adjusted for baseline QTc values) | Day 1 | 1.81(-1.23 - 4.85) | Day 0 (Ref) |  | < 0.001 |
| **Adults**  **(≥  18)** | 0 | 395 (391, 399;346, 440) | 395 (391, 399;331, 449) | -0.3 (-6, 5.4) | 0.92 |
|  | 1 | 397 (393, 401;345, 440) | 397 (393, 401;353, 440) | 0.5 (-5.3, 6.3) | 0.88 |
| *Regression analysis* | | | | | |
| Effect of BE-PEP on QTc  (unadjusted for baseline QTc values) | Day 1 | 0.45(-5.36 - 6.27) | Day 0 (Ref) |  | < 0.001 |
| Effect of BE-PEP on QTc  (adjusted for baseline QTc values) | Day 1 | 0.67(-3.39 - 4.73) | Day 0 (Ref) |  | < 0.001 |
| **Children** | 0 | 390 ([386, 394; 362, 426) | 387 (383, 391; 356, 426) | 2.4 - (-2.9, 7.7) | 0.36 |
|  | 1 | 395 (391, 399; 351, 450) | 390 (386, 394; 353, 435) | 5.1 (-0.7, 10.9) | 0.086 |
| *Regression analysis* | | | | | |
| Effect of BE-PEP on QTc  (unadjusted for baseline QTc values) | Day 1 | 5.08(-0.73 - 10.88) | Day 0 (Ref) |  | 0.048 |
| Effect of BE-PEP on QTc  (adjusted for baseline QTc values) | Day 1 | 3.53(-1.14 - 8.2) | Day 0 (Ref) |  | 0.004 |

*This p-value tests the (unilateral) hypothesis that BE-PEP causes more QTc prolongation than SDR-PEP; a value below 0.025 means that this hypothesis is rejected. BE-PEP= post-exposure prophylaxis (PEP) with bedaquiline 800mg + rifampicin 600mg, SDR-PEP= PEP with rifampicin 600mg only, ms=milliseconds. Regr coeff= Regression coefficient, Ref= reference.
