# Supplementary material for: Safety of single-dose bedaquiline combined with rifampicin for leprosy post-exposure prophylaxis: A Phase 2 randomized non-inferiority trial in the Comoros Islands
Source: PLoS Med. 2024 Oct 21;21(10):e1004453. doi: 10.1371/journal.pmed.1004453 (PMC11534270; doi:10.1371/journal.pmed.1004453)
Supplement: S2 Table — (DOCX) [file pmed.1004453.s005.docx]

**Table S2**. ALT and AST by allocation arm, time point, and age-group.

| **Strata** | **Population** | **Day** | **Measure** | **Pooled** | **BE-PEP**  **( Bedaquiline + Rifampicine)** | **SDR-PEP (Rifampicine single dose)** | **Difference** | **p-value** |
| --- | --- | --- | --- | --- | --- | --- | --- | --- |
| **Adults (≥ 18)** | All | 0 | ALT (IU/L) | 43.99 (42.02 - 45.96) | 45.11 (42.13 - 48.09) | 42.67 (39.37 - 45.97) | 2.437 (-1.264 - 6.138) | 0.278 |
| **Adults (≥ 18)** | All | 0 | AST (IU/L) | 49.08 (47.05 - 51.11) | 50.43 (47.06 - 53.8) | 48.25 (45.03 - 51.47) | 2.187 (-1.69 - 6.063) | 0.352 |
| **Adults (≥ 18)** | ITT | 14 | ALT (IU/L) | 45.89 (44.14 - 47.64) | 46.3 (43.82 - 48.78) | 45.47 (42.95 - 47.99) | 0.837 (-2.13 - 3.804) | 0.642 |
| **Adults (≥ 18)** | ITT | 14 | AST (IU/L) | 51.68 (49.81 - 53.55) | 52.45 (49.83 - 55.07) | 50.91 (48.2 - 53.62) | 1.539 (-1.624 - 4.702) | 0.422 |
| **Adults (≥ 18)** | PP | 14 | ALT (IU/L) | 46.27 (44.43 - 48.11) | 46.47 (43.9 - 49.04) | 46.07 (43.39 - 48.75) | 0.395 (-2.696 - 3.486) | 0.833 |
| **Adults (≥ 18)** | PP | 14 | AST (IU/L) | 52.09 (50.13 - 54.05) | 52.6 (49.88 - 55.32) | 51.56 (48.69 - 54.43) | 1.04 (-2.252 - 4.332) | 0.602 |
| **Children** | All | 0 | ALT (IU/L) | 18.3 (16.91 - 19.69) | 17.44 (16.06 - 18.82) | 19.05 (16.31 - 21.79) | -1.611 (-4.163 - 0.942) | 0.297 |
| **Children** | All | 0 | AST (IU/L) | 27.99 (27.06 - 28.92) | 27.1 (25.61 - 28.59) | 28.81 (27.46 - 30.16) | -1.716 (-3.381 - -0.051) | 0.09 |
| **Children** | ITT | 14 | ALT (IU/L) | 18.4 (16.01 - 20.79) | 18.55 (15.37 - 21.73) | 18.24 (14.56 - 21.92) | 0.305 (-3.731 - 4.341) | 0.901 |
| **Children** | ITT | 14 | AST (IU/L) | 28.08 (26.57 - 29.59) | 28.25 (25.8 - 30.7) | 27.91 (26.09 - 29.73) | 0.332 (-2.202 - 2.867) | 0.828 |
| **Children** | PP | 14 | ALT (IU/L) | 18.37 (15.93 - 20.81) | 18.71 (15.43 - 21.99) | 18.03 (14.31 - 21.75) | 0.677 (-3.434 - 4.787) | 0.785 |
| **Children** | PP | 14 | AST (IU/L) | 28.09 (26.59 - 29.59) | 28.59 (26.1 - 31.08) | 27.6 (25.87 - 29.33) | 0.99 (-1.525 - 3.505) | 0.515 |
