# Supplementary figures and images for: Safety of single-dose bedaquiline combined with rifampicin for leprosy post-exposure prophylaxis: A Phase 2 randomized non-inferiority trial in the Comoros Islands
Source: PLoS Med. 2024 Oct 21;21(10):e1004453. doi: 10.1371/journal.pmed.1004453 (PMC11534270; doi:10.1371/journal.pmed.1004453)

**Figure S1.** Baseline distribution of ALT in screened individuals


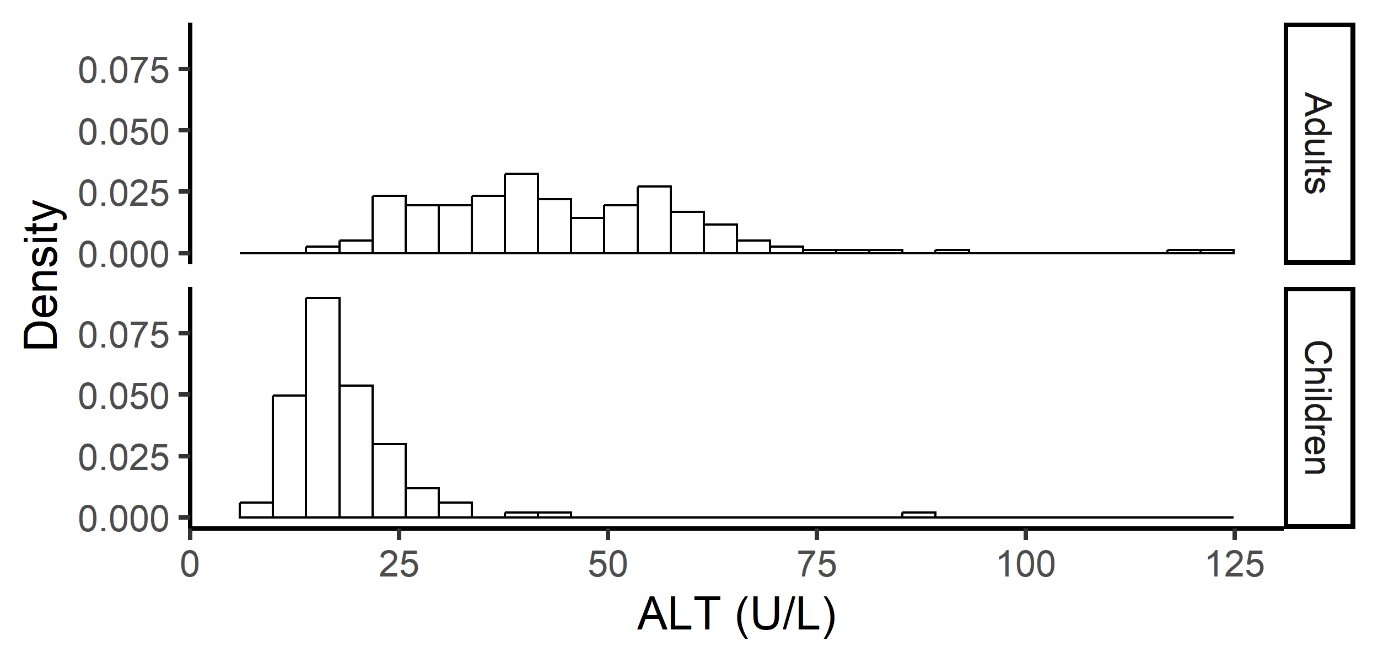

Supplement: S1 Fig — (DOCX) [file pmed.1004453.s006.docx]

**Figure S2.** Baseline distribution of AST in screened individuals


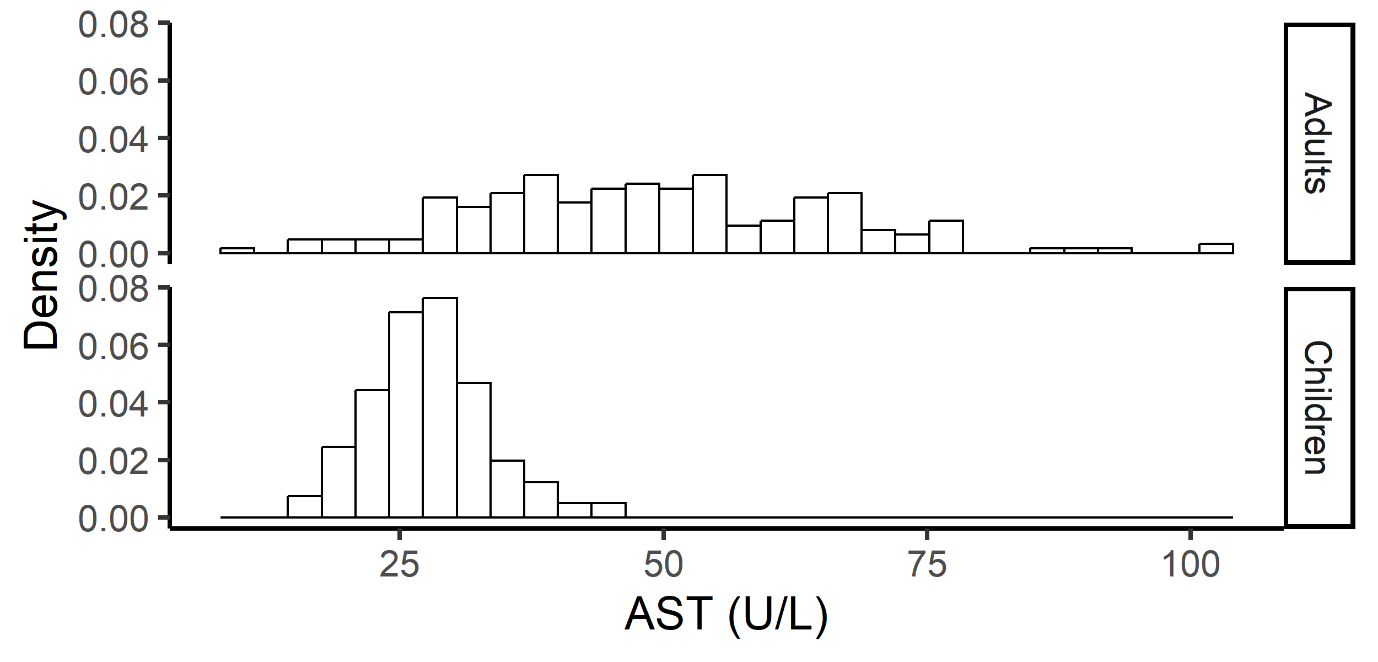

Supplement: S2 Fig — (DOCX) [file pmed.1004453.s007.docx]
